# Dual microglia effects on blood brain barrier permeability induced by systemic inflammation

**DOI:** 10.1038/s41467-019-13812-z

**Published:** 2019-12-20

**Authors:** Koichiro Haruwaka, Ako Ikegami, Yoshihisa Tachibana, Nobuhiko Ohno, Hiroyuki Konishi, Akari Hashimoto, Mami Matsumoto, Daisuke Kato, Riho Ono, Hiroshi Kiyama, Andrew J. Moorhouse, Junichi Nabekura, Hiroaki Wake

**Affiliations:** 1 Division of System Neuroscience, Kobe University Graduate School of Medicine, Kobe, Japan; 2 Division of Homeostatic Development, National Institute for Physiological Sciences, National Institutes of Natural Sciences, Okazaki, Japan; 3 Department of Physiological Sciences, The Graduate School for Advanced Study, Hayama, Japan; 4 Department of Anatomy, Division of Histology and Cell Biology, Jichi Medical University, Tochigi, Japan; 5 Division of Ultrastructural Research, National Institute for Physiological Sciences, Okazaki, Japan; 6 Department of Functional Anatomy and Neuroscience, Nagoya University Graduate School of Medicine, Nagoya, Japan; 7 Section of Electron Microscopy, Supportive Center for Brain Research, National Institute for Physiological Sciences, Okazaki, Japan; 8 Department of Developmental and Regenerative Biology, Nagoya City University Graduate School of Medical Sciences, Nagoya, Japan; 9 Department of Anatomy and Molecular Cell Biology, Nagoya University Graduate School of Medicine, Nagoya, Japan; 10 School of Medical Sciences, The University of New South Wales, Sydney, NSW Australia; 11 Core Research for Evolutional Science and Technology, Japan Science and Technology Agency, Saitama, Japan; 12 Precursory Research for Embryonic Science and Technology, Japan Science and Technology Agency, Saitama, Japan

**Keywords:** Blood-brain barrier, Microglia

## Abstract

Microglia survey brain parenchyma, responding to injury and infections. Microglia also respond to systemic disease, but the role of blood–brain barrier (BBB) integrity in this process remains unclear. Using simultaneous in vivo imaging, we demonstrated that systemic inflammation induces CCR5-dependent migration of brain resident microglia to the cerebral vasculature. Vessel-associated microglia initially maintain BBB integrity via expression of the tight-junction protein Claudin-5 and make physical contact with endothelial cells. During sustained inflammation, microglia phagocytose astrocytic end-feet and impair BBB function. Our results show microglia play a dual role in maintaining BBB integrity with implications for elucidating how systemic immune-activation impacts neural functions.

## Abstract (web summary)

Although it is known that microglia respond to injury and systemic disease in the brain, it is unclear if they modulate blood–brain barrier (BBB) integrity, which is critical for regulating neuroinflammatory responses. Here authors demonstrate that microglia respond to inflammation by migrating towards and accumulating around cerebral vessels, where they initially maintain BBB integrity via expression of the tight-junction protein Claudin-5 before switching, during sustained inflammation, to phagocytically remove astrocytic end-feet resulting in impaired BBB function

## Introduction

Peripheral organ status and systemic circulation are increasingly recognized as factors that impact cognitive performance and neuronal disease^1,2^. This can range from acute severe illness and fever causing malaise and poor cognition, from sepsis causing subsequent neurological disease and cognitive failure^3^, through to chronic systemic inflammation associated with smoking, diabetes, chronic periodontitis and even aging, leading to an increased risk of dementia or neurodegenerative disorders^4,5^. Characterizing the physiological and molecular mechanisms by which systemic inflammation impacts on brain function may improve understanding of brain pathophysiology and open up new therapeutic targets. One important pathway by which systemic inflammation or infection may initiate neuronal consequences is via communication across the blood–brain barrier (BBB) between systemic inflammatory or immune molecules and the neuronal elements involved in maintenance of neural circuits.

The BBB is a tightly regulated syncytium of endothelial cells with low transcellular and paracellular transport properties that surround cerebral vessels and protect the delicate neuronal microenvironment from neurotoxic substances. Endothelial transport is strictly regulated through interactions with astrocytes, pericytes, microglia, and the basement membrane—together forming a neurovascular unit that constitutes the BBB^6^. Astrocytes and pericytes directly encircle endothelial cells and not only help link blood supply to metabolic demand but also secrete a number of molecules that enhance and maintain BBB integrity^7–10^. Microglia are part of the neurovascular unit although their ablation in the mature mouse does not increase BBB permeability^11^. Nevertheless, their ablation can adversely influence BBB integrity via release of cytokines or reactive oxygen species^12,13^. Increases in BBB permeability are seen in many neurological and psychiatric disorders, including stroke, epilepsy, amyotrophic lateral sclerosis, Alzheimer’s disease, Parkinson’s disease, multiple sclerosis, and schizophrenia, suggesting BBB compromise is involved in the pathogenesis and/or severity of brain disease^7,14^. Reactive microglia (and astrocytes) are likely to contribute to the leaky BBB observed in these diseases through downregulation of paracellular tight-junction proteins such as Claudin-5 (CLDN5), occludin, and zonula occludens-1 (refs. ^7,9^). Conversely, microglia can be driven towards reactive phenotypes in these diseases by direct neuronal damage, via systemic factors, or through neutrophils that invade the damaged BBB^15^ making it difficult to correlate specific microglia phenotypes with changes in BBB. Furthermore, as characterized in neuronal diseases and brain trauma, phagocytic microglia may engulf endothelial cells and other components of the neurovascular unit^16,17^.

Systemic inflammation and infection can also result in microglial phenotype changes and disruption of BBB integrity in the absence of any precipitating neuronal damage or neuronal infection^14^. Given the importance of physiological microglia in monitoring and sculpting neural circuits in development and adult learning, the activation of microglia into different phenotypes may potentially disrupt their homeostatic function and contribute to the development of adverse cognitive effects due to systemic inflammation^11,18^. Indeed, we have recently reported that microglia activated by experimentally induced systemic inflammation (using lipopolysaccharide [LPS] injections) lose their capacity to synchronize local neural circuits^19^. Furthermore, in a recent study using a mouse model that replicates the pathogenic inflammation in systemic lupus erythematosus (SLE), reactive microglia phagocytosed cortical synapses which correlated to observed cognitive deficits^20^. An important initial step in identifying how systemic infection or inflammation signals convert resident physiological microglia, and how changes in BBB permeability impact this process, is to correlate the temporal changes in BBB leakage with resulting microglial responses. In this study, we attempted to achieve this goal by simultaneously measuring BBB permeability and microglia dynamics during both acute and chronic systemic infection using in vivo two-photon imaging. We demonstrated that microglia respond to inflammation by migrating towards and accumulating around cerebral vessels, and that this begins before any detectable change in BBB permeability. Surprisingly, our data suggest that the initial microglial contact with cerebral blood vessels actually protect BBB integrity. Further prolonged inflammation results in a dominance of a more activated microglial phenotype, resulting in phagocytosis of astrocytic end-feet and a loss of BBB permeability. Our results implicate microglia as playing a dual role in BBB permeability during systemic infection and inflammation, with important implications for understanding how systemic diseases may adversely impact on neural circuits and brain functions.

## Results

### Microglia-vessel accumulation and BBB leak in MRL/lpr mice

**Fig. 1:**
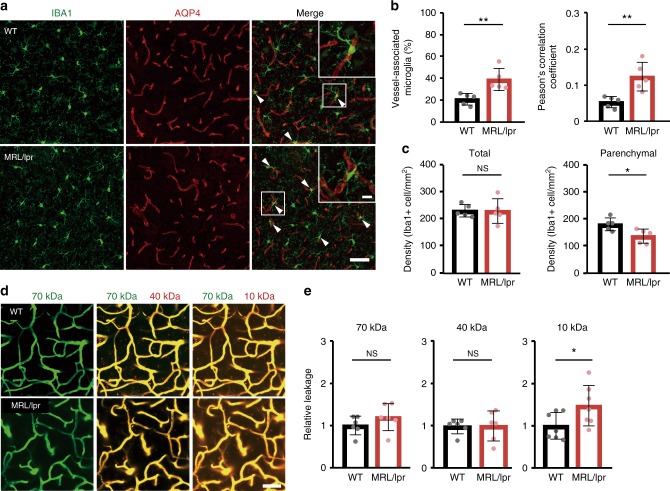
Systemic inflammation is associated with a leaky BBB and a close association between microglia and cerebral blood vessels. **a** Fluorescent images of IBA1 (microglia, green) and AQP4 (astrocyte end-feet marker, red) in WT mice and in mice with chronic systemic inflammation (MRL/lpr mice). Upper right boxes in right panels show magnified images of a typical blood vessel with associated microglia. Scale bar, 50 and 10 μm for magnified image. Arrowheads indicate a contact between microglia and blood vessels. **b** The proportion of microglia in contact with blood vessels and the extent of overlap of IBA1 and AQP4 fluorescence as quantified by a Pearson’s correlation coefficient were both higher in MRL/lpr mice than in WT mice. **c** The density of parenchymal microglia (right panel) is decreased in MRL/lpr mice, whereas the total density of microglia in the whole field of view (left panel) is the same in WT and MRL/lpr mice. **d**, **e** BBB permeability was quantified from the leaking of different molecular size (10, 40, 70 kDa) dextran-conjugated fluorophores into the parenchymal space outside the vessels, with each dextran fluorescing at a different color (and pseudo-colored here as green or red). Vessels were identified by the impermeant 70 kDa fluorophore (green), while leak identified by leakage (red) of fluorescence outside the co-labeled (yellow) vessels. Representative images (left) indicate that blood vessels in WT mice were impermeant to all these fluorophores and only the blood vessels themselves fluoresce (upper panels). Vessels in MRL/lpr mice are permeant to the 10 kDa dextran, which is visible in the parenchymal space adjacent to, and outside, the vessels (lower panels). Relative leakage is quantified by comparing mean parenchymal fluorescence intensity in MRL/lpr mice with that in WT mice (normalized to 1.0). In MRL/lpr mice, vessels leak only the smaller 10 kDa dextran. Scale bars in **d**, 50 μm. Graphs show data from an individual animal (**b**, **c**, **e**), overlaid with mean ± SD. NS not significant. *P < 0.05 and **P < 0.01.

To investigate how microglia and BBB permeability respond to systemic inflammatory and immune challenge, we initially utilized the MRL/lpr (Murphy Roths Large/lymphoproliferation) mouse model of SLE. The MRL/lpr mouse develops well-characterized cognitive and behavioral abnormalities that are associated with elevated neurotoxic inflammatory cytokines auto-antibodies, and which exhibits a leaky BBB that exacerbates the access of these damaging mediators^20,21^. Using immunohistochemical staining with antibodies against IBA1 (Ionized calcium-binding adapter molecule 1: a microglia marker) and AQP4 (aquaporin-4: an astrocyte protein used here to also mark vessels), we visualized microglia and blood vessels within the motor cortex of MRL/lpr mice, observing an increased number of microglia in close association with cerebral vessels (Fig. 1a, b). The proportion of microglia in contact with vessels (vessel-associated microglia/total microglia) increased in MRL/lpr mice compared with WT mice (Fig. 1b). We also quantified the colocalization of vessels and microglia using Pearson’s correlation coefficient, a measure of the extent to which each fluorescent pixel for AQP4 (vessels) and IBA1 (microglia) are co-located. This colocalization coefficient significantly increased in MRL/lpr mice as compared with WT mice (Fig. 1b). Overall microglial density was similar in each image field between WT and MRL/lpr mice (Fig. 1c), but the density of microglia within the brain parenchyma was reduced in MRL/lpr mice (Fig. 1c), suggesting that resident microglia have migrated to cerebral vessels. We next quantified BBB permeability, which is known to be increased in SLE model mice^22^, by measuring the relative fluorescence of different molecular size dextran conjugates in parenchyma following intravenous injection concurrent with in vivo imaging (Fig. 1d; see also Supplementary Fig. 1 for methodology). The averaged relative leakage of different size dextrans from vessels into the parenchymal in WT mice was indexed as 1.00. There were no significant differences in the BBB permeability between WT and MRL/lpr mice for 40 or 70 kDa fluorophores (Fig. 1e). We also did not detect any extravasation of fibrinogen (~340 kDa) in fixed brain sections from either WT or MRL/lpr mice (Supplementary Fig. 2). However, the BBB permeability of the 10 kDa fluorophore was significantly increased in MRL/lpr mice, which is consistent with an increase of cytokines in the SLE model mice^20^. Hence chronic inflammation induces an increase in BBB permeability to smaller compounds.

### Time course of microglia migration and changes in BBB leak

**Fig. 2:**
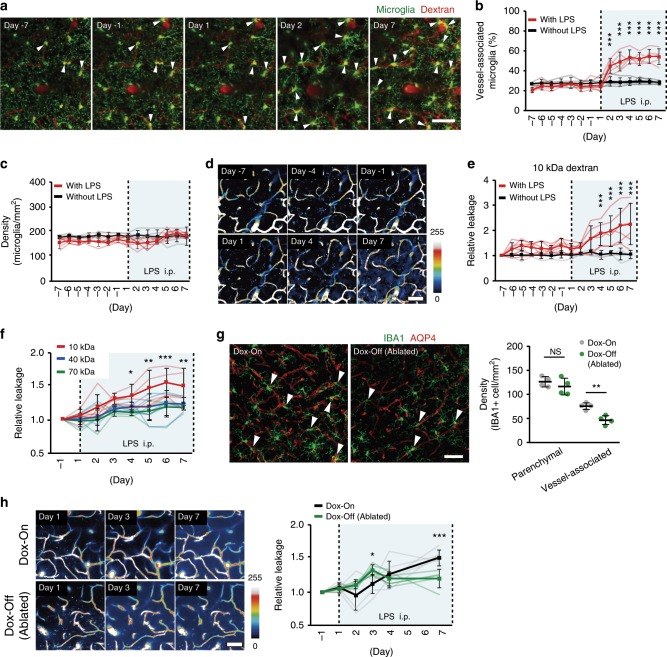
The temporal relationship between microglia-vessel migration and BBB permeability during systemic inflammation. **a** Image series from a single mouse demonstrate the time course of microglia migration to cerebral vessels during the development of systemic inflammation induced by daily LPS injections. **b** Effects of daily LPS injections on the proportion of microglia in contact with cerebral vessels. Graphs show data from control (without LPS) and LPS (with LPS) experiments. Blue shadow indicates LPS injections (i.p.). **c** Effects of LPS on the total number of microglia in each image field on each day. LPS had no significant effect on total microglial density. **d** A series of typical images from a single mouse demonstrates the time course of changes in BBB permeability after systemic LPS injection. **e** Effects of daily LPS injections or control on dextran leakage from blood vessels. The relative leakage of 10 kDa dextran was significantly increased from 4 to 7 days after LPS injection. **f** Quantification of the size selectivity of BBB permeability after systemic LPS injection using different molecular size dextrans. **g** Typical images (left panels) and mean data (right panel) show the effects of partial microglia ablation (Dox-Off) on the density of microglia in the brain parenchyma and associated with vessels. The density of vessel-associated microglia was significantly decreased in Dox-Off mice as compared with Dox-On mice. **h** A series of typical images from a single Dox-On (upper panels) and Dox-Off (lower panels) mouse illustrate the effects of microglia ablation on BBB permeability during systemic inflammation induced by daily LPS injection. Time course of BBB permeability changes during LPS injections Dox-Off and Dox-On mice. Microglia ablation induced a significant increase in BBB permeability during the early phase of systemic inflammation (Day 3) and a significant reduction in the later phase. In all line graphs, light lines indicate data from an individual animal (**b**, **c**, **e**, **f**, **h**), while the dark lines and error bars show mean ± SD. NS not significant. *P < 0.05, **P < 0.01 and *** *P* < 0.001. Scale bars in all panels: 50 μm.

To probe the temporal relationship between microglia accumulation at blood vessels and the increases in BBB permeability, we gave daily LPS injections (1 mg/kg, intraperitoneal; i.p.) to better control the onset and development of the systemic inflammatory response^23,24^. Again, we simultaneously visualized microglia dynamics and BBB permeability, now using the Cx3cr1-GFP^24^ mice, in which microglia express GFP. Mice were injected with the differently sized fluorescent dextrans and in vivo two-photon imaging was performed daily, for 1 week prior to LPS administration to gather baseline data, and then for another week during the daily LPS (or saline) injections (Fig. 2a). During the baseline control week, microglia were relatively stable. Their processes occasionally made brief contact with blood vessels as part of their continued parenchymal surveillance as previously reported^25,26^. About 20% of microglia made contact with blood vessels during this baseline control period, although their soma did not migrate towards vessels and the number of microglia associated with vessels did not increase (Fig. 2a–c). Within 1 day of LPS injections, however, some microglia had migrated towards vessels (Supplementary Video 1, in comparison to Supplementary Video 2) and the proportion in contact with vessels increased significantly during the week of LPS injections, as compared with the absence of LPS (Fig. 2b). There was no increase in total microglia cell numbers during this time (Fig. 2c). We also did not detect any systemic macrophages invading the central nervous system (CNS) during this time nor did we observe perivascular macrophages migrating along vessels (Supplementary Video 1). Permeability of the BBB (to 10 kDa dextran) remained constant during the control week, but increased significantly (Fig. 2d) on days 4 through 7 after starting LPS injections (Fig. 2e, Supplementary Videos 3 and 4). We also examined BBB permeability by testing dextran of different sizes, but increases in parenchymal fluorescence were only seen with the 10 kDa dextran (Fig. 2f). Again, we also failed to detect any leakage of fibrinogen in fixed brain after 7 days LPS injection (Supplementary Fig. 2). Pericytes are an important component of the neurovascular unit and have been reported to detach from vessels in response to systemic inflammation induced by high (20 mg/kg) doses of LPS^27^. We did not observe any changes in pericyte immunofluorescence or cell density (quantified as the number of PDGFRβ+’ve DAPI+’ve double-positive cells), nor in blood vessels coverage by pericytes, in either the MRL/lpr mice nor after LPS injections (Supplementary Fig. 3). Even a single LPS injection (1 mg/kg, i.p.) was enough to elicit an increase in vessel-associated microglia, although this did not cause an increase in BBB permeability to 10 kDa fluorescent dextran (Supplementary Fig. 4a, b). In summary, the systemic inflammation induced by intraperitoneal injection of LPS caused microglia to migrate towards cerebral vessels, which was followed by an increased BBB permeability.

### Depletion of microglia has dual effects on BBB permeability

To clarify the consequences of microglia-vessel accumulation on BBB permeability, we partially ablated microglia using the Iba1-tetracycline transactivator (Iba1-tTA)::tetracycline operator-diphtheria toxin A (tetO-DTA) mouse (Iba1-tTA::tetO-DTA) mouse. Upon withdrawal of doxycycline from the feed (Dox-Off) of these mice, lethal diphtheria toxin A was expressed in microglia^28^. In our conditions, Dox withdrawal (Dox-Off) predominantly reduced vessel-associated microglia (Fig. 2g), as measured on LPS injection Day 7, after 14 days of Dox-Off diet. The density of microglia in the parenchyma was not significantly different at this time (Fig. 2g). A slight increase in GFAP immunofluorescence was also seen, suggesting some possible astrocyte activation or proliferation associated with the loss of microglia and/or systemic inflammation and BBB leak (Fig. 2g, Supplementary Fig. 5a–c).

This fortuitous selective loss of vessel-associated microglia in the Dox-Off mice is likely to arise due to the higher IBA1 expression in the more reactive vessel-associated microglia. Regardless, it enabled us to examine how ablation of vessel-associated microglia affected BBB permeability during 7 consecutive days of LPS injection Parenchymal fluorescence (BBB leak) was expressed relative to that at Day −1 of LPS (Fig. 2h; cf. Fig. 2e). Unexpectedly, the loss of microglia actually increased BBB permeability during the early phase (Day 3) of LPS-induced inflammation (Fig. 2h). Dox withdrawal, in the absence of LPS, did not change BBB permeability (Supplementary Fig. 5d). As LPS injections continued and BBB permeability further increased, the protective effect of microglia was reversed, and partial ablation caused a reduced BBB permeability at Day 7 (Fig. 2h).

### Characterizing vessel-associated microglia

**Fig. 3:**
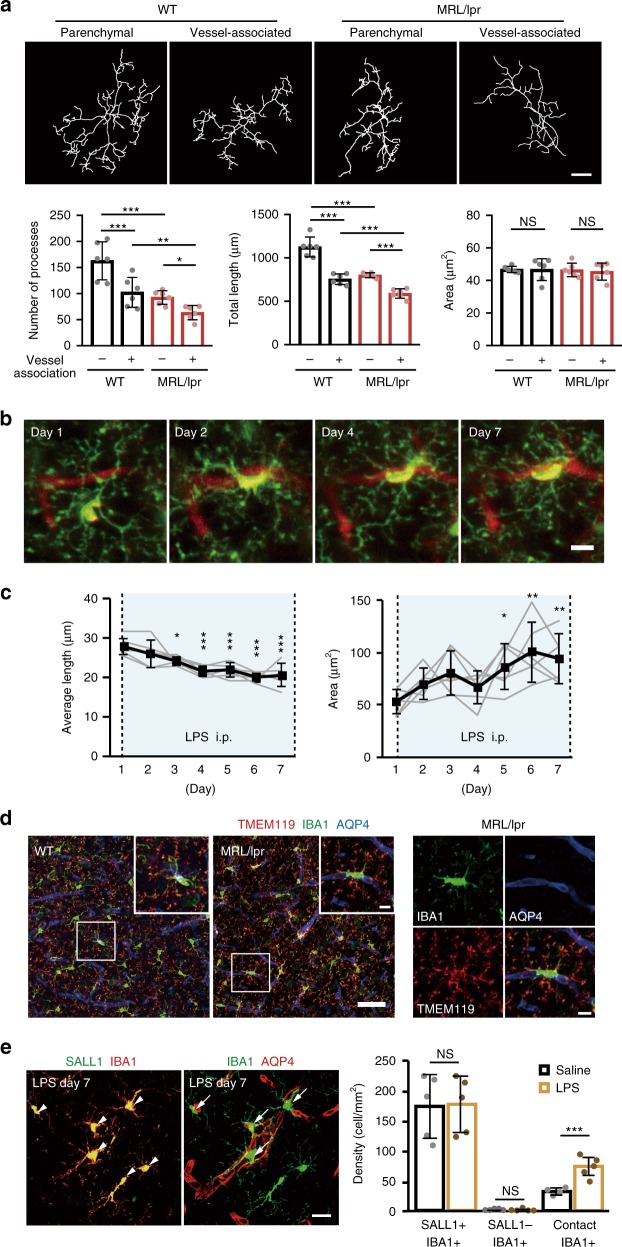
Changes in microglia morphology and dynamics during systemic inflammation. **a** Different properties of microglia process in vessel-associated and parenchymal microglia in MRL/lpr mice. Upper panels show topological skeletonized images of microglia from WT (left) and MRL/lpr mice (right) in parenchyma or associated with vessels. Microglia were visualized by IBA1 immunostaining and the processes were traced by ImageJ. Scale bar, 10 μm. Bottom panels quantify the number of process (left panel) and the mean cumulative length (center panel) of microglia processes in each mouse and the mean area of the cell soma. **b** A series of typical images from a mouse during LPS injections showed microglial (green) migration to a vessel (red) and the associated changes in microglia morphology. Scale bar, 10 μm. **c** Quantitative analysis of the mean length of microglial processes (left panel) and the mean microglial soma area (right panel) during 7 days of LPS injections. **d** Typical images of microglia stained positive for both IBA1 and TMEM119 in WT (left) and in MRL/lpr (middle) mice. Boxes at the top right show magnified images of the region indicated in the main panel. The right-hand panel shows the individual fluorescent channels (and merged channel) for the magnified image from the MRL/lpr mouse. **e** Typical images of microglia positive for both IBA1 and SALL1 (left) or IBA1 and AQP4 (right) after 7 days of LPS injections to quantify the progeny of vessel-associated microglia. The right panel shows mean data for the density of cells identified by IBA1 or SALL1. The total number of resident brain microglia counts (SALL1+’ve) or infiltrating macrophages/microglia (SALL1−’ve, IBA1+’ve) were not altered by LPS, while the number of resident microglia associated with vessels (contact IBA1+’ve, colocalized with AQP4) was increased by LPS. Scale bar, 20 μm. In all graphs, each point indicates data from an individual animal (**a, c, e**), while columns and error bars show mean ± SD. NS not significant. *P < 0.05, **P < 0.01, and ***P < 0.001.

In the SLE model mice (MRL/lpr), both parenchymal and vessel-associated microglia had significantly shorter processes and reduced branches as compared with those of WT mice, although the soma area was similar (Fig. 3a). These morphological changes suggest a different microglia phenotype is present in the SLE model mice. For both WT and the SLE model mice, microglia associated with vessels showed a distinct, more reactive, phenotype than those in the parenchyma (Fig. 3a). This prompted us to examine changes in microglia morphology during the 7-day consecutive LPS injection schedule. As microglia migrated to vessels, they adopted a different morphological phenotype (Fig. 3b). Quantification of these parameters revealed a gradual decrease in process length and an increase in the soma area (Fig. 3c). The mean process length got significantly shorter from Day 3 onwards, while cell soma areas significantly increased from Day 5 onwards. Hence the more reactive characteristics of microglial morphology correlates to the timing when the BBB becomes leaky, but not to the period when microglia first migrate to vessels and have a protective effect on BBB permeability.

Finally, we examined the lineage phenotype of vessel-associated microglia, to test if this population includes cells derived from systemic or perivascular macrophages, as has been suggested^29,30^. We first immunostained the cells with TMEM119, a selective microglia marker that does not stain macrophages. All the IBA1 positive cells in a field of view were also TMEM119 positive (e.g., Fig. 3d). We next used the Sall1-GFP mouse, where GFP is only expressed in brain resident microglia that derive from the yolk sac^31^. Virtually all microglia expressed both SALL1 and IBA1, and the proportion of double-labeled microglia did not change after 7 days of LPS (Fig. 3e). Similarly, the very small numbers of IBA1+’ve and SALL1−’ve microglia did not change. Taken together, this suggests that macrophages do not contribute significantly to the vessel-associated microglia population. Based on the increased spatial correlation of IBA1 and AQP4 immunohistochemistry, we confirmed that LPS injections in Sall1-GFP mice induced a significant increase in the microglia population associated with vessels (Fig. 3e).

### Inflammation induces expression of Claudin-5 in microglia

**Fig. 4:**
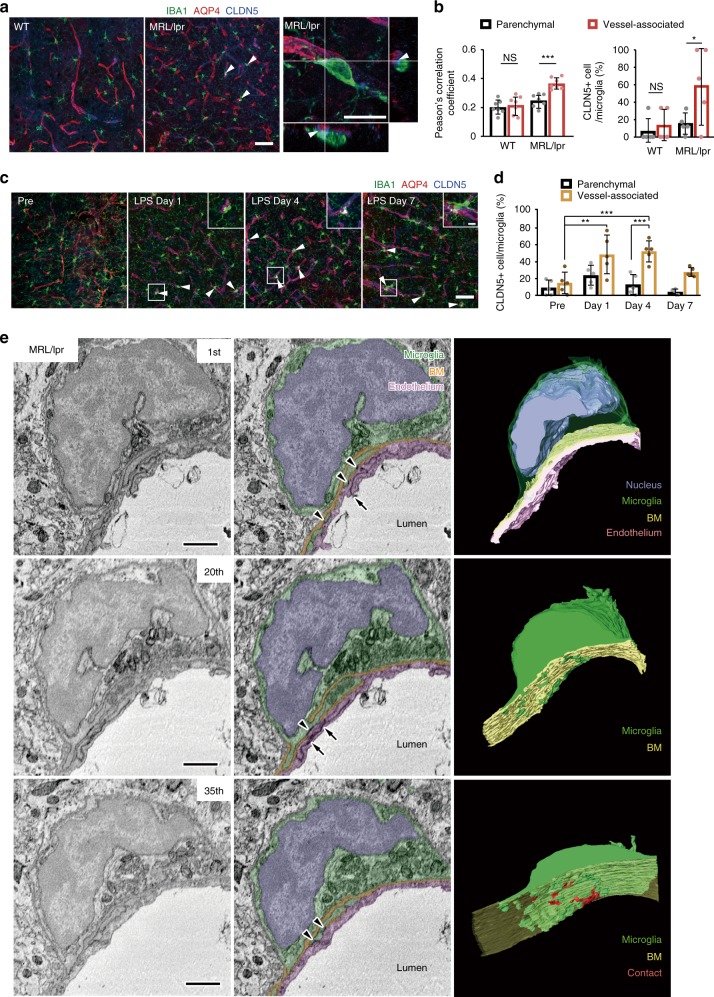
Biochemical and structural characterization of microglia associated with BBB protection during early systemic inflammation. **a** Typical immunohistochemical images show colocalization of immunofluorescence for IBA1 (green), the tight-junction marker Claudin-5 (CLDN5, blue), and the astrocytic end-feet marker aquaporin-4 (AQP4, red) in WT and in MRL/lpr mice, with magnified images at the different fluorescent channels shown adjacent. Arrowheads indicate vessel-associated microglia expressing CLDN5 around its perimeter (expanded in the right-most panel) while the arrowhead shows a CLDN5 in microglia. The right-most panel shows an orthogonal view of a microglia (green) associated with astrocytic end-feet (red) expressing CLDN5 (blue) in the contacting microglia. Scale bar, 50 μm, or 10 μm (insets). **b** The proportion of microglia that are positive for CLDN5 among vessel-associated microglia was higher in MRL/lpr mice. **c** A series of immunohistochemistry images show the effects of LPS on the colocalization of IBA1, AQP4, and CLDN5. Arrowheads indicate microglia showing colocalization with CLDN5. Scale bar, 50 or 10 μm (inset). **d** Graph showing the proportion of parenchymal (black) and vessel-associated microglia (ocher) that express CLDN5 on different days during the progression of systemic inflammation induced by daily LPS from Day 1. **e** Representative CLDN5 immuno-electron microscopic images of a blood vessel in an MRL/lpr mouse and the surrounding neurovascular unit. Left column shows raw images, center column shows identified components, and the right column shows 3D reconstruction, of the 1st, 20th, and 35th serial sections. A microglial cell (green) surrounding the surface of the basement membrane (BM, orange-yellow) has small processes that infiltrate the BM to form immunoreactive contacts (arrowheads) with endothelial cells (pink), which resemble the immune-reactive contact between endothelial cells(arrows, tight junctions). The 3D reconstruction illustrates protrusions through the BM (center) and the endothelial cell contacts (lower panel, red). Scale bars: 1 μm. In all graphs, each point indicates data from an individual animal (**b, d**), while columns and error bars show mean ± SD. NS not significant. *P < 0.05, **P < 0.01, and ***P < 0.001.

**Fig. 5:**
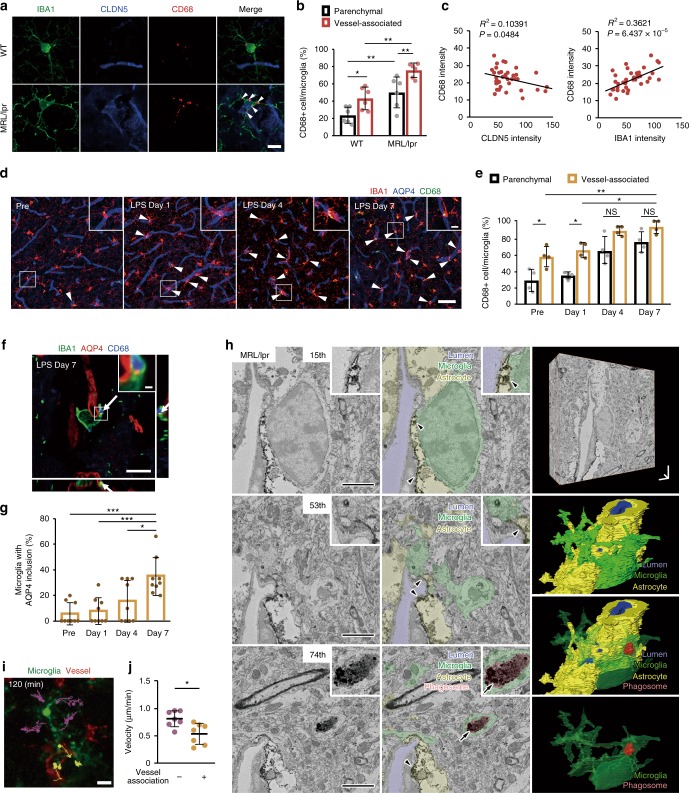
Characterization of phagocytic microglia during late systemic inflammation. **a** Typical immunohistochemistry images showing colocalization of IBA1, CLDN5, and CD68 immunofluorescence. Arrowheads in the merged image indicates CD68+’ve puncta in microglia. **b** Quantification of the proportion of parenchymal and vessel-associated microglia that express CD68 was significantly increased in MRL/lpr mice and in MRL/lpr vessel-associated microglia. **c** Correlations between the expression intensities of CLDN5 and CD68 (left), and IBA1 and CD68 (right), for vessel-associated microglia in MRL/lpr mice. **d** A series of immunohistochemistry images showing the effects of LPS on the colocalization of IBA1, AQP4, and CD68. Arrowheads indicate microglia with triple colocalization for these markers. Scale bar, 50 μm. **e** Graph shows the proportion of parenchymal and vessel-associated microglia that express CD68 with systemic inflammation. **f** Orthogonal view of a microglia associated with astrocytic end-feet (AQP4) showing AQP4 and CD68 double-positive vesicles in a contact microglia (as indicated by arrow) suggesting phagocytosis of astrocyte end-feet components. Scale bar, 3 μm (inset). **g** Plots show the proportion of vessel-associated microglia in which such AQP4 puncta were observed with systemic inflammation. **h** Sample AQP4 immuno-electron microscopic images of neurovascular unit in an MRL/lpr mouse. Left column shows raw images (insets show magnified immunopositive segments), and the center column shows identified components while the right column shows 3D reconstruction. A microglial cell is shown directly adjacent to the surface of the capillary (lumen), which is surrounded by astrocyte end-feet (arrowheads). The lower panel shows an immunopositive microglial phagosome (red, arrow), demonstrating astrocyte fragments as microglial inclusions. Scale bars: 2 μm. **i** Typical images to quantify the effects of vessel contact on microglial process motility. Yellow and purple dots and line trajectories indicate the process movements in two different microglia. **j** The graph shows the microglial process motility. In all graphs, each point indicates averaged data from an individual animal (**b**, **c**, **e**, **g**, **j**), while columns and error bars show mean ± SD. NS not significant. *P < 0.05, **P < 0.01 and ***P < 0.001. Scale bar in **a**; **d**, inset; **f**; **i** are 10 μm.

To probe possible interactions between microglia and the BBB during inflammation, we compared the genetic profiles of astrocytes and microglia in WT and MRL/lpr mice (Supplementary Fig. 6). Numerous (>200) genes were upregulated in MRL/lpr mice, including those involved in immune responses, cell adhesion, and phagocytosis. We identified two genes and products for further study: CLDN5 (Claudin-5) and CD68. CLDN5 contributes to the tight junctions between adjacent endothelial cells in the BBB^32,33^, while CD68 is a lysosome marker expressed in phagocytic macrophages and activated microglia^34^. We immunostained CLDN5 and CD68 in WT and MRL/lpr mice and during LPS injections in WT mice (Figs. 4 and 5). CLDN5 fluorescence showed colocalization with AQP4 expression, as expected given its association with the neurovascular unit, but also showed co-localization with microglia in MRL/lpr mice (Fig. 4a). The colocalization of CLDN5 and IBA1 immunoreactivity and the proportion of CLDN5+’ve microglia were higher in vessel-associated microglia in MRL/lpr mice, as compared with parenchymal microglia (Fig. 4b, Supplementary Fig. 7). We next examined the temporal patterns of CLDN5 expression during 7 days of LPS injections (Fig. 4c, d). Prior to LPS, the population of CLDN5+’ve IBA1+’ve microglia was minimal among both parenchymal and vessel-associated microglia (Fig. 4c, d). However, at 1 day after LPS injection, a significant but transient increase in the population of CLDN5+’ve IBA1+’ve microglia was seen in vessel-associated microglia (Fig. 4d, Supplementary Fig. 7). To verify that the CLDN5 and IBA1 colocalization represented CLDN5 expression within microglial cells, we fluorescence activated cell sorted (FACS) microglial and endothelial cells using Cx3cr1-GFP and CD31 fluorescent antibodies, respectively, and probed these two populations for CLDN5 expression (Supplementary Fig. 8). The number of CD31 and CLDN5 positive cells did not differ between MRL/lpr and WT mice, or between control and LPS (single injection) mice (Supplementary Fig. 8). However, Cx3cr1-GFP and CLDN5 positive cells were increased in MRL/lpr mice and in response to single LPS injection (Supplementary Fig. 8b). This indicates CLDN5 expression is specifically induced in microglia, which was further supported by in vitro results (see below). Finally, we performed immuno-electron microscope (immuno-EM) analysis to determine if microglia expressing CLDN5 formed tight junctions with the vessel endothelium. The high-resolution analysis in MRL/lpr mice vessels indicated that microglial processes could infiltrate the basal membrane and contact endothelial cells (Fig. 4e). Hence, in the early phase of inflammation, microglia express CLDN5 and can send processes through the basal membranes to contact endothelial cells, strongly suggesting tight junctions are formed to protect the integrity of the BBB.

### Microglia express phagocytic proteins in late inflammation

As indicated above, the gene analysis had also suggested expression of the phagocytic marker CD68 in MRL/lpr mice microglia (Supplementary Fig. 6), so we next focused on the characteristics and functional consequences of this. Immunohistochemistry demonstrated increased CD68+’ve and IBA1+’ve cells, both in parenchymal and vessel-associated microglia of MRL/lpr mice, although to a greater extent in vessel-associated microglia (Fig. 5a, b). This CD68 expression pattern resembled the pattern of changes in morphological phenotype in MRL/lpr microglia, but contrasted with the pattern of increased CLDN5 colocalization, which was restricted to vessel-associated microglia. Indeed, the expression intensity of the two markers was negatively correlated (Fig. 5c) while the expression intensity of CD68 was positively correlated to that of IBA1 (Fig. 5c), suggesting CD68 expression depends on the microglia activation state. Expression of CD68 was preferentially seen in vessel-associated microglia, and after LPS administration, expression increased across both parenchymal and vessel-associated microglia, masking any differences by Day 4 and Day 7 (Fig. 5d, e). Again, a different pattern of expression was seen with CLDN5 and CD68, suggesting different roles of microglia during the progression of inflammation. As increased CD68 expression and the morphological changes implicated a phagocytic phenotype, we looked for AQP4-positive puncta in microglia as a marker for possible astrocyte end-feet phagocytosis (Fig. 5f–h). We observed some AQP4+’ve puncta colocalized with IBA1+’ve microglia, and the proportion of these microglia that contained colocalized puncta of AQP4 fluorescence increased significantly after 7 days of LPS injections (Fig. 5f). We again used the higher resolution immune-EM to determine if these AQP4 puncta were phagocytosed microglia inclusions. We identified microglial phagosomes, and frequently observed AQP4 immunoreactivity in these phagosomes (Fig. 5h). If microglia are phagocytosing astrocyte end-feet in advanced LPS-induced inflammation, one may predict a concurrent decrease in microglia-process motility when they are associated with vessels. After 7 days LPS, motility was indeed reduced for microglia associated with vessels as compared with parenchymal microglia (Fig. 5i, j).

### Chemokine CCL5 induces microglial migration to vessels

**Fig. 6:**
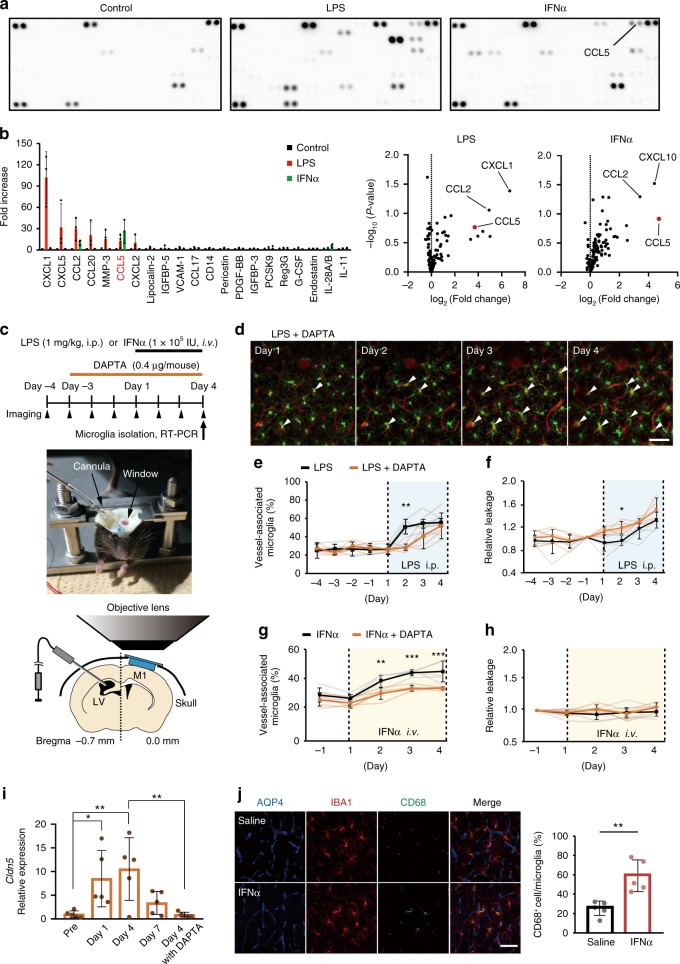
Signaling pathways mediating microglia migration to vessels and subsequent BBB integrity regulation. **a** Sample cytokine array data from control cultured endothelial cells, and cultured endothelial cells treated with LPS or IFNα. Each column represents immunoblots for 111 different cytokines. **b** The top 21 cytokines that changed with the presence of LPS or IFNα compared to control cells. CCL5 increased with both treatments. **c** Schematic experimental paradigm and photos and schematics showing the intraventricular DAPTA injection protocol that accompanied the daily in vivo imaging and intraperitoneal LPS injection paradigm. **d** Typical in vivo images of microglia migration to vessels at different stages of LPS injection protocol in a mouse treated with intraventricular DAPTA injection. **e**, **f** Plot of the proportion of microglia in contact with cerebral vessels (**e**) and relative change in BBB permeability (**f**), LPS injection protocol (indicated by blue shading) in a mouse treated with the CCR5 antagonist, DAPTA, and compared to the mean values for control mice (LPS only, dashed lines). DAPTA delayed microglia migration and caused earlier loss of BBB integrity. **g**, **h** As in **e**, **f**, the effects of DAPTA on the proportions of vessel-associated microglia (**g**) and leaks across the BBB (**h**) in response to intravenous injection of IFNα. DAPTA reduced vessel migration, although no effect was observed on BBB permeability. **i** The effect of LPS injections on microglial CLDN5 mRNA expression levels after daily injections of LPS compared with the presence of DAPTA. DAPTA treatment significantly suppressed CLDN5 expression in microglia. **j** Typical immunohistochemistry of CD68 and colocalization with vessels (AQP4) and microglia after intraparenchymal saline or IFNα injection. The right panel graph quantifies the proportion of microglia colocalized with CD68, showing microglial CD68 increased significantly after intraventricular IFNα injection. Each point in graphs (**i**, **j**), and the faint lines in graph (**e, f, g, h**), indicate data from a single field, while the columns (**i, j**) or dark lines (**e, f, g, h**), and error bars show mean ± SD. NS not significant. *P < 0.05, **P < 0.01, and *** *P* < 0.001. Scale bars in all panels: 50 μm.

We speculated that endothelial cells may respond to systemic inflammation and release molecules to trigger microglia migration and phenotype changes. To identify a potential signaling mechanism, we treated cultured endothelial cells with LPS or interferon (IFN)α and analyzed the cytokines that were released into the culture media. We found that CCL5 was significantly increased in both conditions (Fig. 6a, b) and moved to in vivo experiments to examine signaling via this chemokine pathway. We administered the CCR5 antagonist (DAPTA, 0.4 μg/mice, 1 μl) via intraventricular injection for 3 days prior to, and concurrently with 4 days of LPS (1 mg/kg, i.p.) or IFNα injection (1 × 10^5^ IU/mouse, i.v.) (Fig. 6c). DAPTA treatment delayed the migration of microglia to vessels, resulting in a significant decrease in the number of vessel-associated microglia compared to LPS alone after 2 days of LPS (Fig. 6d, e). DAPTA also caused an earlier onset of LPS-induced BBB leak (observed at day 2; Fig. 6f), together suggesting DAPTA inhibits both microglial migration and the resulting initial BBB protection. Intravenous injection of IFNα also promoted microglia migration to vessels, which was again inhibited by intraventricular DAPTA injection (Fig. 6g). Although systemic IFNα induced microglia migration to vessels, it did not induce any change in BBB integrity (Fig. 6h). In contrast, intraparenchymal injection of IFNα increased the proportion of microglia expressing CD68 (Fig. 6j). CCR5 inhibition with DAPTA also completely prevented the LPS-induced increases in microglia CLDN5 expression in vivo (Fig. 6i).

We further validated and extended our proposed signaling mechanisms using cultured microglia. We detected both basal Cldn5 and Ccr5 mRNA expression in microglial cultures from adult mouse brains, but not in microglial cultures from neonatal brains (Supplementary Fig. 8d). Incubation of these cultures with CCL5 (100 ng/ml) significantly increased Cldn5 mRNA expression in adult cultures, but not in neonatal cultures (Supplementary Fig. 8d). Consistently, neonatal cultures did not express the Ccr5. Furthermore, Ccr5 expression in adult cultures was time-dependent, gradually decreasing to negligible levels within 6 h of isolation of microglia from the brain (see Methods) (Supplementary Fig. 8e). The culture approach we used has been reported to produce a reasonably pure (95–98%) microglial population^35,36^, and we confirmed this using flow cytometry (Supplementary Fig. 8f). Notably the CD31+’ve population was negligible. Taken together, these in vivo and in vitro results suggest CCL5-CCR5 signaling contributes to attracting microglia to vessels and inducing microglial CLDN5 expression and subsequent BBB protection in the early stages of inflammation. Once BBB integrity is lost as inflammation progresses, IFNα may be able to invade the brain and trigger microglia to adopt a phagocytic phenotype.

### Inhibiting microglial activation maintains BBB integrity

**Fig. 7:**
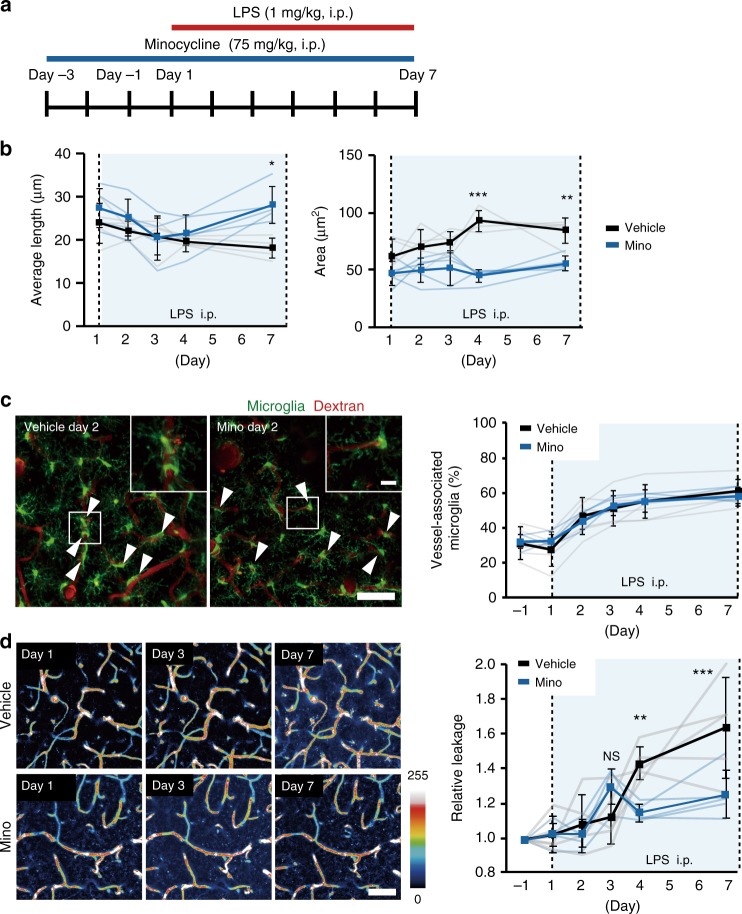
Inhibition of microglial activation improves BBB integrity during the later phase of systemic inflammation. **a** Schematic diagram of the experimental protocol. Minocycline (Mino) (or vehicle) was injected 3 days before LPS injections. From Day 1, LPS and Mino (or vehicle) was co-injected daily for 7 days. Imaging was performed before (Day −1) and on days 1, 2, 3, 4, and 7 days after LPS injections. **b** Graph plotting the effects of LPS on the mean length of microglial processes and on the mean area of microglia cell soma in vehicle and Mino-treated mice. **c** Typical in vivo two-photon images (left) and mean data (right) showed the effects on LPS on the proportion of microglia-associated with vessels. Mino did not affect the migration and accumulation of microglia at vessels. Scale bar, 50 or 10 μm (inset). **d** (Left) A series of typical in vivo two-photon images from a single vehicle- (upper) or Mino- (lower) treated mouse demonstrated the changes in BBB permeability at different days after systemic LPS injection, measured by leakage of 10 kDa dextran from vessels. Scale bar, 50 μm. (right) Plot of the effects of daily LPS injections on the dextran leakage from vessels. The relative leakage of 10 kDa dextran was significantly increased at 7 days after LPS injection in vehicle mice compared to Mino-treated mice. In each graph the faint lines indicate data from an individual animal (**b, c, d**) while the dark lines and error bars indicate the mean ± SD. NS not significant. *P < 0.05, **P < 0.01, and ***P < 0.001.

Our results suggest that microglia have dual effects on BBB integrity—initial protection of BBB integrity but then as inflammation progresses microglia develop a phagocytic phenotype to increase BBB permeability. To further test this conclusion, we inhibited reactive microglia using Minocycline (Mino)^37,38^. Mino (75 mg/kg, i.p.) or vehicle was administrated before, and during, the LPS injection schedule (Fig. 7a). Mino prevented the decrease in process length and the increase in soma area seen during LPS injections (Fig. 7b), confirming an inhibition of microglial reactivity. The mean length of microglia processes in vehicle mice was longer at the end of the 7 days LPS compared with the same time in the Mino group (Fig. 7b). The mean cell body area was also significantly larger in the control group as compared to the Mino group, on both 4 and 7 days. Mino itself (without LPS) did not affect microglial process lengths or soma areas (Supplementary Fig. 9). Although activation of microglial were blocked by Mino, as expected, the microglia still migrated towards blood vessels and the proportion of microglial associated with vessels did not differ significantly between the vehicle and Mino groups (Fig. 7c). As we observed previously, continued LPS injections gradually increased the leakage of 10 kDa dextran fluorophores from vessels into the parenchyma, and this leakage was significantly reduced by Mino (Fig. 5d). After 4 and 7 days of LPS injection, the relative leakage was higher in control mice compared with that of in Mino mice. Again Mino by itself (without LPS) did not affect the microglia migration (Supplementary Fig. 9) or BBB leak (Supplementary Fig. 9). Finally, we examined if motor learning was affected in MRL/lpr mice using a self-initiated lever-pull forelimb task^39,40^ (see also the Methods). Mice performed this task for 60-min per session (one session per day) over 12 days. There were no differences between WT littermates and MRL/lpr mice in either the success rates or the number of successful lever pulls during the early training stages (Supplementary Fig. 10). However, in the late training stage (days 11–14), the success rate and number of successful lever pulls were lower in MRL/lpr mice than in WT mice (Supplementary Fig. 10), indicating impaired motor learning.

## Discussion

Microglia are active surveyors of brain parenchyma with important roles in sculpting and coordinating neural circuits in healthy brains that respond rapidly to form a range of reactive phenotypes in brain infection and damage^19,25,26,41^. Activated microglia play roles in a range of acute and neurodegenerative diseases, where they can help clear neuronal damage by phagocytosis, but can also contribute to disease progression by releasing molecules that can initiate a neuroinflammatory states^12,42,43^. Microglia can also respond to peripheral inflammatory diseases. A key question is how microglia change phenotypes when the primary pathological insult resides in the peripheral organs and systemic circulation. Determining how these systemic and neuronal inflammatory responses are linked may help reduce the deleterious impact of systemic immune activation and inflammation on cognitive function and susceptibility to brain disease. The BBB represents a major pathway by which systemic inflammation and immune responses potentially interact with the brain microenvironment. The goal of this study was to examine the role of microglia in responding to systemic infection and inflammation, and its contribution to BBB integrity. Using two different models of peripheral inflammation, MRL/lpr mice and mice treated for 7 days with LPS, we demonstrated that resident brain microglia migrate to cerebral vessels during systemic inflammation in response to the release of the chemokine CCL5 from endothelial cells. This triggers microglial cells to express CLDN5 and to infiltrate through the neurovascular unit, thus contacting endothelial cells and forming tight junctions to maintain BBB integrity. Consistently, partial microglial ablation or blocking CCL5 signaling, actually increased BBB permeability during the early stages of inflammation. Additional factors may also trigger microglial migration to blood vessels and phenotype changes under different conditions. Fibrinogen, for example, can attract microglia to vessels in murine models of experimental autoimmune encephalomyelitis^44^. Sustained inflammation causes microglia to further transform into a phagocytic phenotype associated with morphological changes, engulfment of astrocytic fragments, and leakage across the BBB. Partial ablation of these vessel-associated microglia reduced the BBB leakage, as did inhibition of reactive microglia with minocycline. Minocycline reduces BBB leak associated with rodent models of brain diseases, such as hypoxia, ischemia, and Alzheimer’s disease^45–47^ and our results extend this to BBB leak resulting from systemic inflammation. Minocycline can also improve cognitive function in both mouse models of ischemia and human stroke^48^. Protection from microglia-induced initiation or exacerbation of BBB leak may contribute to such therapeutic effects^38,49^. Indeed, limiting BBB leakage can reduce access to the brain microenvironment for a range of toxic circulating molecules, such as inflammatory cytokines, ions, and immune cells. These mediators will further (directly and indirectly via neuronal damage) activate microglia and astrocytes and exacerbate neuroinflammatory damage and BBB integrity^50^.

Our data indicate opposing actions of microglia in regulating BBB integrity with distinct time courses and underlying signaling pathways. The CCL5-CCR5-mediated increase in microglial CLDN5 expression, and the evidence of microglia infiltrating through the neurovascular unit, is consistent with a novel tight-junction connection between endothelial cells and microglia to mediate protective sealing of the BBB. CLDN5 forms tight junctions between adjacent endothelial cells in a complex of other claudin proteins and occludins^32^. Upregulation of members of the CLDN family (Cldn4, Cldn1) in reactive astrocytes has also been shown to contribute to new tight junctions in inflammatory diseases, and furthermore to reduce the extent of neuropathology^51^. Indeed, our microarray data also suggest that astrocytes adopt a more reactive phenotype, which may also contribute to regulating the permeability of the BBB, either independently or in concert with microglia. Astrocytes, BBB integrity, and blood flow regulation are all closely linked, and astrocyte reactivity may be a key component in both acute and chronic brain diseases associated with altered BBB permeability^52^. Altered CLDN5 expression also regulates BBB integrity, where a decrease in CLDN5 increases BBB leakage. For example, microglia activated in response to epileptic seizures release interleukin-1β (IL-1β) which downregulates the tight-junction protein CLDN5 in endothelial cells that then disrupts BBB function^53^. Our results suggest novel CLDN5 upregulation in microglia maintains BBB integrity via proposed microglial tight junctions with endothelial cells. Our results contradict the notion that microglia do not express this tight-junction protein. For example, whole-brain RNA seq data suggest microglial expression is almost negligible^54^ (see http://www.brainrnaseq.org/). However, CCL5-CCR5-dependent Cldn5 expression is likely to be restricted to a small subset of vessel-associated microglia in response to specific and possibly transient inflammatory signaling pathways, and hence challenging to detect in whole brain samples. Indeed, more recent single-cell RNA sequencing clearly shows Cldn5 expression in some genotypically characterized microglial subsets, even in healthy brain^55^ (see also https://myeloidsc.appspot.com/).

The second aspect of the microglia–BBB interaction we observed was transformation of vessel-associated microglia to a CD68-expressing phagocytic phenotype with loss of BBB integrity. Activated microglia are known to contribute to BBB leakage in neuroinflammatory diseases, via cytokine release or by upregulating adhesion molecules to facilitate invasion of circulating immune cells^15,56,57^. For example, systemic inflammation induced by bacterial infection and stress activate microglia which then release cytokines tumor necrosis factor-α and IL-1β^15,58^. These trigger endothelial cells into expressing adhesion molecules that facilitate invasion of systemic immune cells and subsequently enable more pronounced neuroinflammation. Our results add an additional facet in explaining how reactive microglia can cause neuroinflammation. By phagocytizing the astrocytic end-feet during more sustained systemic inflammation, the integrity of the BBB is compromised. Disruption of astrocytes in Alzheimer’s disease and Parkinson’s disease also results in a leaky BBB^59^. However, the increased permeability of the BBB in our LPS and MRL/lpr models is more subtle than seen in some Alzheimer’s and hypoxia or ischemia models, where much larger molecules like albumin-conjugated Evan’s Blue dye (~66 kDa) and IgGs (~150 kDa) can permeate the BBB, and the coverage of vessels by pericytes can also be substantially decreased in these diseases^45–47^. Type I interferon (IFN) has been suggested to mediate the link between systemic inflammation in SLE mice, and the conversion of resident parenchymal microglia to a phagocytic phenotype^20^, and IFN may have also contributed to the activation of microglia we observed in our study. A small cytokine (such as IFNα) is consistent with the modest leakiness of the BBB we observed. We did not detect peripheral macrophage accumulation around blood vessels. Indeed, our EM analysis showed that microglial processes protruded through the basement membrane and even occasionally appeared to obtrude through to the vessel wall where IFNα or another systemic cytokine may directly initiate microglia transformation into a reactive phenotype that then amplifies the inflammatory response. A cellular communication pathway involving endothelial cells may also be involved.

In conclusion, our study sheds light on how brain microglia respond to systemic inflammation and interact with the BBB. They initially migrate to the BBB and protect its integrity, before then transforming into a reactive phenotype that phagocytose BBB components to initiate leakage of systemic substances into the parenchyma and cause widespread neuroinflammation. We also identified some key molecules and signaling pathways involved in instigating the opposing microglial responses. This may lead to strategies that will maintain the BBB integrity during systemic disease, and thereby reducing susceptibility to cognitive disorders and/or the adverse neural effects associated with peripheral infection, and systemic stress and inflammation.

## Methods

### Strains and animal care

Experimental protocols were approved by the Animal Care and Use Committees of Kobe University Graduate School of Medicine and the National Institutes of Natural Sciences. Experiments were conducted according to the guidelines of the National Institutes of Health *Guide for the Care and Use of Laboratory Animals*. We used male mice for all experiments to avoid potential variations during estrus cycles. All of the animals in this study were given free access to food and water and housed under 12 h light/dark cycle conditions. We used ICR (WT) mice and MRL/MpJJmsSlc-lpr/lpr (MRL/lpr) mice as a model of autoimmune disorders, such as SLE and Sjogren’s syndrome, which were purchased from Japan SLC (Shizuoka, Japan). To visualize the morphology and motility of microglia, we used Cx3cr1-EGFP transgenic mice expressing enhanced green fluorescent protein (EGFP) controlled by of Cx3cr1 promoter which is specific for microglia, macrophages, and monocytes^60^. We then backcrossed Cx3cr1-EGFP mice onto MRL/lpr mice for more than six generations to generate MRL/lpr::Cx3cr1-EGFP mice, which developed lymphadenopathy and splenomegaly at the age of 9 weeks. To further visualize the resident microglia, we used Sall1 reporter mice (Sall1-GFP)^31^. For microglia ablation experiments, we crossed Iba1-tetracycline transactivator (Iba1-tTA) mice^28^ with tetracycline operator-diphtheria toxin A (tetO-DTA) mice^61^. All transgenic mice were derived from the C57BL/6J strain.

### Surgery

We performed the first surgery in 6–8-week-old mice. Following anesthesia with ketamine (74 mg/kg, i.p.) and xylazine (10 mg/kg, i.p.) the skull was exposed and cleaned, and a custom-made head plate was firmly attached to the skull with dental cement (G-CEM ONE; GC, Tokyo, Japan). This head plate allowed us to securely attach the mouse to a stainless frame for two-photon imaging in the awake state or perform the circular craniotomy. One or two days after plate attachment, we performed a circular craniotomy (2 mm diameter) over the left primary motor cortex (M1, centered at 1 mm lateral from the bregma) under isoflurane (1%) anesthesia^40^. After the craniotomy, 2% (w/v) agarose L dissolved (Nippon Gene, Tokyo, Japan) in saline was applied, and a glass window comprising two coverslips (2 and 4 mm each; Matsunami Glass, Osaka, Japan) were placed over the brain surface with ultraviolet curable adhesive (NOR-61, Norland). The edges of the cranial window were sealed with a combination of dental cement and dental adhesive resin cement (Super Bond; Sun Medical, Shiga, Japan). Mice were housed individually and imaging experiments started around 3 weeks after the surgical treatments (i.e., 9–11-week-old).

### Two-photon imaging

Two-photon images were acquired from the left M1 using a laser scanning system (LSM 7 MP system; Carl Zeiss, Oberkochen, Germany) with two types of water-immersion objective lenses (×10, numerical aperture (NA) 0.5; ×20, NA 1.0; Carl Zeiss) and a Ti:sapphire laser (Mai Tai HP; Spectra-Physics, Santa Clara, CA) operating at a 950-nm wavelength. Fluorescence was separated by a 570-nm dichroic mirror with 495–550 nm (green channel: for EGFP fluorescence detection) and 570–630 nm (red channel: for Texas Red fluorescence detection) emission filters and collected using GaAsP photomultiplier tubes (Hamamatsu Photonics, Shizuoka, Japan). To assess BBB permeability, we used a laser scanning system (NIS-Elements; Nikon Instech Co., Ltd, Tokyo, Japan) with a water-immersion objective lens (×25, NA 1.10; Nikon Instech Co., Ltd) and a mode-locked Ti:sapphire Chameleon Ultra II laser (Coherent, Santa Clara, CA) set at 950 nm. Fluorescence was separated with two dichroic mirrors (560-nm dichroic mirror with 500–550 nm [green channel: for fluorescein fluorescence detection] and 563–588 nm [red channel: for tetramethylrhodamine (TMR) fluorescence detection] emission filters; 593-nm dichroic mirror with 601–657 nm [magenta channel: for Texas Red fluorescence detection] emission filters).

### In vivo quantification of BBB permeability

Cerebral blood vessels were visualized using dextran-conjugated fluorophores. To estimate disruption of the BBB caused by systemic inflammation (see below), we injected fluorescence-tagged dextrans into the tail vein during imaging. Three different dextran beads of increasing size: 10 kDa dextran-Texas Red, 40 kDa dextran-tetramethylrhodamine (TMR), and 70 kDa dextran-fluorescein (all at concentrations of 2 mg/ml; Thermo Fisher Scientific, Waltham, MA). None of these dextrans would cross an intact BBB so the presence of fluorescence in the brain parenchyma in response to a dextran injection indicated BBB disruption. To quantify the degree of disruption, we reconstructed 23 stacks of individual *Z*-plane images acquired after each dextran injection with each focal plane separated by 2 μm where each frame was a resolution of 1024 × 1024 pixels. The borders of the vessels in the *Z*-stacks were mapped out using the Maximum Entropy Threshold function in ImageJ (National Institutes of Health, Bethesda, MD). This was combined with the Analyze Particles function to delineate the vessel borders (using settings of size = 40-Infinity (μm^2^), and include holes). This allowed quantification of the mean fluorescence intensity outside the defined vessels. These values were expressed relative to WT in the original *Z*-projected image. Supplemental Figure 1 schematically illustrates the approach to quantify BBB leakage.

### Systemic drug administration

LPS (Funakoshi, Tokyo, Japan) was administered to induce systemic inflammation. Single daily injections of LPS (1.0 mg/kg, i.p.) were repeated for 7 days after two-photon imaging under control conditions (7 consecutive days before LPS injections). Control mice received i.p. saline injections under the same dosing schedule. To produce a milder LPS model, we also prepared a single LPS injection model (1.0 mg/kg, i.p., one shot throughout the experiment).

The Iba1-tTA::tetO-DTA mice were reared with chow containing Dox 100 mg/kg. Withdrawal of the doxycycline (Dox-Off) in the feed induced selective expression of the diphtheria toxin A (DTA) in microglia, resulting in cell death. The Dox-containing chow was replaced by Dox-free standard chow 7 days before starting two-photon imaging during the consecutive 7-day LPS injections.

Minocycline hydrochloride (Mino; M9511-1G; Sigma-Aldrich, St. Louis, MO) was administered to inhibit microglial activation. Single daily injections of Mino (75 mg/kg, i.p.) were started 3 days prior to LPS injections and continued for 10 consecutive days. Control mice received i.p. vehicle (5% dimethyl sulfoxide in saline) injections under the same LPS dosing schedule. We also created mice that had received the 10-day Mino injections but not the LPS injections.

Systemic interferon-alpha (IFNα) was also injected to trigger the release of CCL5, which could regulate microglial motility (see the main text). A 1 × 10^5^ IU dose of recombinant mouse IFNα (12100-1; R&D systems, Minneapolis, MN) dissolved in 100 μl of sterile saline was intravenously injected.

### Intraparenchymal drug injection

To identify what triggers expression of CLDN5 and CD68 in microglia (also see the main text), we injected intraparenchymal IFNα. Under isoflurane (1%) anesthesia, a glass pipette (GDC-1; Narishige, Tokyo, Japan) was stereotaxically inserted into the left primary motor cortex. A 600 IU dose of recombinant mouse IFNα (12100-1; R&D system) dissolved in 600 nl of sterile saline was injected. As the control, 600 nl of sterile saline was injected in the right primary motor cortex.

### Intraventricular drug injection

To identify which factor attracted microglia to blood vessels, we performed daily intraventricular injections of an inhibitor of the most likely signaling molecule. For the intraventricular injection, we implanted a combination of guide (CXG-8; Eicom, Kyoto, Japan) and dummy cannula (CXD-8; Eicom) in the right lateral ventricle after creating the glass window. D-Ala-peptide T-amide (DAPTA; R&D systems, Minneapolis, MN), a selective antagonist for CCL5 receptor CCR5 (refs. ^62,63^), was injected into the ventricle through an injection canula (CXMI-8; Eicom) attached to an electrically driven injection pump (UMP3; WPI, Sarasota, FL). Single daily injections of DAPTA (1 μl; 0.4 μg/μl in saline) were started 3 days prior to LPS injections and continued for 7 consecutive days.

### Immunohistochemistry

Animals were deeply anesthetized with ketamine and xylazine, and transcardially perfused with 4% paraformaldehyde solution in phosphate-buffered saline (PBS0. Fixed brains were extracted from the skull and post-fixed overnight in the same solution followed by 30% sucrose. The brains were microtomed (Leica Microsystems, Wetzlar, Germany) into 30 μm slices. After blocking and permeabilization for 1 h in 5% bovine serum albumin (BSA) and 0.5% Triton X-100 in PBS, the slices were incubated at 4 °C overnight with primary antibodies diluted in PBS. After PBS wash, slices were subsequently incubated with secondary antibodies in PBS at room temperature (RT) for 3 h. Slices were then mounted on glass slides in Fluoromount-G (Southern Biotech, Birmingham, AL). Fixed tissue was imaged using a Zeiss LSM510 Meta confocal microscope (Carl Zeiss) with a ×20 objective (NA 1.0; Carl Zeiss) or a ×63 oil-immersion objective (NA 1.4). The following antibodies were used for staining: anti-IBA1 (019-19741, Wako, Osaka, Japan; ab5076, Abcam, London, UK; 1:400), anti-AQP4 (AB3594, Merck KGaA, Darmstadt, Germany; 1:500), anti-CLDN5 (35-2500, Thermo Fisher Scientific; 1:100), anti-CD68 (MCA1957GA, Bio-Rad, Hercules, CA; 1:400), anti-PDGFRβ (14-1402-82, Thermo Fisher Scientific; 1:100), anti-TMEM119 (400-011, Synaptic Systems, Göttingen, Germany; 1:100), anti-CD31 (14-0311-81, Thermo Fisher Scientific; 1:100), anti-GFAP (AB53554-100, Abcam; 1:400), anti-Fibrin (A0080, DAKO, Santa Clara, CA; 1:250), anti-rabbit Alexa 488, anti-rabbit Alexa 594, anti-goat Alexa 488, anti-goat Alexa 594, anti-mouse Alexa 594, anti-rat Alexa 555 (Molecular Probes, Eugene, OR; 1:500 for each), anti-mouse Alexa 405 and anti-rat Alexa 405 (Abcam; 1:500 for each). To visualize the cerebral vasculature, the brain slices were incubated with DyLight 488-labeled *Lycopersicon esculentum* lectin (DL1174, Vector laboratories, Burlingame, CA, USA; 1:200) at RT for 1 h^64^.

### Image analysis

Images were analyzed using ImageJ (National Institutes of Health) and MATLAB (MathWorks, Natick, MA) software packages. Movies and 3D images were corrected for focal plane displacement using ImageJ plug-in TurboReg and StackReg^65^. Microglia in the superficial layer of the motor cortex (100–200 μm from the pia, corresponding to layer II/III) were analyzed in *Z*-projected images (confocal image: 1024 × 321 pixels, 0.312 μm/pixel, 2 μm *Z*-step [rostrocaudal direction], 10 slices, maximum intensity projection; two-photon image: 1024 × 1024 pixels, 0.254 μm/pixel, 2 μm *Z*-step [dorsoventral direction], 50 slices). The imaging depth was determined to satisfy the following criteria: (1) a depth (i.e., 200 μm) at which clear two-photon images of microglia and vessels could be monitored with moderate laser power (low enough to avoid brain tissue damage); (2) we divided the captured images into four sections (each section, 50 μm), and estimated the microglia in each section; (3) the two most superficial layers (0–50 μm and 50–100 μm from the surface) were excluded to avoid the possible surgical damage, and (4) the 100–150 μm section and 150–200 μm section contained equal numbers of microglia, and so the data from these two sections (100–200 μm) were combined. Using the ImageJ plug-in, Simple Neurite Tracer, we then quantified microglial morphology based on the reconstructed *Z*-stacked confocal images. The number of microglial processes and total lengths were determined from confocal 3D image data (1024 × 1024 pixels, 0.139 μm/pixel, 0.5 μm *Z*-step, 30 slices). Cell body areas were quantified using the segmented line tool in ImageJ. To define vessel-associated microglia, (1) a vertical line was drawn from the presumed center of the microglia the surface of a blood vessel, (2) signal intensities of microglia-associated green fluorescence (EGFP) and blood vessel-associated red fluorescence (Texas Red) were calculated along the ROI line using the Multiplot function in ImageJ, (3) the standard deviation of fluorescent intensity was subtracted from the original signal for each channel, and (4) if the distance along the microglia–blood vessel axis was below 4 pixels (1 μm) between the point the green fluorescence decreased to zero and the point the red fluorescence increased from zero, the microglia was defined to have contacted the blood vessel (vessel-associated microglia). We then calculated what percentage of microglia in the captured image were vessel-associated microglia. Using the ImageJ plug-in Coloc2, Pearson’s correlation coefficient was calculated to assess colocalization of IBA1 and AQP4, IBA1 and CLDN5, and CD68 and AQP4. To define CD68+’ve microglia, the number of CD68 puncta were counted in the microglia. More than two of the CD68 puncta in microglia were defined as CD68+’ve microglia.

Colocalization of PDGFRβ and lectin fluorescent signals were used to determine pericyte coverage, defined as a percentage (%) of the PDGFRβ+’ve area overlapping with lectin+’ve vessel surface area in each image frame^64,66^. Pericyte density was quantified as PDGFRβ+’ve and DAPI+’ve cells.

### Isolation of microglia and astrocytes

After euthanasia with ketamine and xylazine, animals were transcardially perfused with PBS to remove circulating blood cells in the CNS. The cortex was cut off and dissociated using Neural Tissue Dissociation Kits (130-093-231; Milteny Biotec, Bergisch Gladbach, Germany). Debris was filtered through a cell strainer (100 μl) with 5% BSA-PBS followed by treatment with debris removal solution (130-109-398; Milteny Biotec). We then used the magnetic-activated cell sorting (MACS) system with CD11b magnetic beads (for microglia, 130-093-634; Milteny Biotec) or anti-GLAST magnetic beads (for astrocyte, 130-095-826; Milteny Biotec) and an MS column (130-042-201; Milteny Biotec). RNA was extracted using RNeasy Plus Mini kit (74134; Qiagen, Hilden, Germany).

### Gene expression analysis

The RNA in microglia and astrocyte was precipitated in the presence of isopropanol and sodium acetate at −80 °C. The RNA pellet was washed with 70% ethanol on ice, air-dried, and resuspended in RNase-free water. Total RNA (1000 ng) from each sample was further quantified by Qubit fluorometric quantitation and analyzed by an Agilent 2100 Bioanalyzer (Agilent Technologies, Santa Clara, CA). The samples were then hybridized to microarray slides with Whole Mouse Genome 4×44K v2 Microarray Kit (G4846A; Agilent Technologies, Santa Clara, CA). Microarray data were analyzed using R-Bioconductor package (open source software). Gene ontology (GO) terms were referenced from online tools in Metascape (http://metascape.org/)^67^.

### Electron microscopy with immunohistochemistry

To examine the 3D structure of microglia and vessels during immunostaining for CLDN5 and AQP4, serial images were acquired with 3D reconstruction using serial block-face scanning electron microscopy (SBEM)^68^. Briefly, the tissues were fixed in 4% paraformaldehyde solution in PBS and post-fixed overnight in the same solution. Brains were cut with a vibratome into 200-μm-thick sections, and freeze-thawed after infiltration of PBS containing 30% sucrose^69^. After blocking for 1 h in 5% BSA in PBS, slices were incubated overnight at 4 °C with primary antibodies against CLDN5 (35-2500, Thermo Fisher Scientific; 1:100) or AQP4 (AB3594, Merck KGaA; 1:500). The slices for CLDN5 immunostaining were heated at 95 °C for 1 min. After PBS wash, slices were incubated in HRP-conjugated secondary antibodies at 4 °C for 3 h in PBS. Immunoreactions were visualized using 0.5 mg/ml diaminobenzidine substrate (Sigma-Aldrich) with 10 mM H_2_O_2_ and enhanced with ice cold 0.04% OsO_4_ in PBS. After additional fixation with 2.5% glutaraldehyde in 0.1 M PB (pH 7.4) at 4 °C for 2 h and washing with ice cold PBS, the slices were further incubated with 2% OsO_4_ in 1.5% potassium ferrocyanide in PBS for 1 h on ice. After washing with double distilled water (dDW), the tissues were then incubated in filtered 1% thiocarbohydrazide solution for 20 min at RT. After washing again with dDW, the tissues were incubated in a newly prepared 2% OsO_4_ for 30 min at RT. The tissues were placed in 2% uranyl acetate at 4 °C overnight and incubated in lead aspartate solution at 65 °C for 30 min after washing with dDW. The tissues were dehydrated in a graded series of ethanol (60%, 80%, 90%, and 99.5% for 5 min each), followed by infiltration with acetone that was dehydrated with a molecular sieve in a 1:1 mixture of resin and acetone and 100% resin. The resin was prepared with a Durcupan kit according to the manufacturer’s instructions (Sigma-Aldrich) and 7% Ketjen black was added to increase Durcupan conductivity^70^. Trimmed sample surfaces were gold sputtered to increase conductivity and imaged on field emission-SEM (Merlin or Sigma, Carl Zeiss AG.) equipped with 3View (Gatan, Inc., Pleasanton, CA). The resulting serial images were handled by ImageJ with Fiji plugins (http://fiji.sc/). Segmentation and image analyses were performed with Microscopy Image Browser (http://mib.helsinki.fi.) and Amira (FEI Visualization Science Group, Hillsboro, OR, USA) software.

Microglial soma were identified using established criteria, which include relatively small and elongated nuclei, with clumped chromatin beneath the nuclear envelope and irregular contours of cytoplasm and processes with lysosomes and long endoplasmic reticulum^71^. The processes of these microglia were then tracked in these 3D images to identify process terminations associated with specific elements of the neurovascular unit.

### Endothelial cell culture

Primary endothelial cell cultures were prepared by the puromycin treatment method^72,73^. The cerebral cortices of mice were minced with collagenase-based digestion medium and cultured with Dulbecco’s modified Eagle’s medium (DMEM) containing 10% fetal bovine serum (FBS) and 30 μg/ml of Endothelial Cell Growth Supplement (E2759; Sigma-Aldrich) in a 24-well plate coated with collagen IV for 5 days. Endothelial cells were selected through puromycin treatment (P8833; Sigma-Aldrich, 8 μg/ml, 3 days). Endothelial cells were washed twice with media before stimulation, and then stimulated (immersed) in a solution containing LPS (100 ng/ml) or IFNα (1000 IU/ml) for 24 h. Culture supernatants were collected after stimulation. The collected supernatant was stored at −80 °C. Cytokine production was measured by Proteome Profiler, Mouse XL Cytokine Array Kit (ARY028; R&D systems).

### Microglial culture

Isolation of primary adult microglial cultures was adapted from Singh et al.^35^. Briefly, the whole-brain tissues of adult mice (C57BL/6, 9 weeks old) were dispersed with papain-based digestion medium and the microglia-rich fraction was isolated using Percoll (17-0891-02, GE Healthcare, Uppsala, Sweden) density gradient centrifugation. The microglial pellet was washed and cells were seeded on to 24- or 96-well plates in DMEM containing 10% FBS and incubated at 37 °C for 10 min, and then the plate was washed with culture medium to remove non-adherent cells. The purity of CD11b+’ve microglia was confirmed by flow cytometry. The adherent microglia were maintained in DMEM containing 10% FBS for 0–6 h, or stimulated for 1 h with 100 ng/ml CCL5 (594202, BioLegend, San Diego, CA, USA) before being collected for real-time PCR analysis. Primary neonatal microglia were prepared from a primary mix-culture^36^. The cerebral cortices of neonatal mice (C57BL/6, 0 days old) were minced and treated with trypsin-based digestion medium, and then dispersed cells were cultured in DMEM containing 10% FBS in culture flask (75 cm^2^; 658170, Greiner Bio-One, Kremsmünster, Austria) in a 5% CO_2_ humidified incubator at 37 °C for 10–12 days. Microglia were detached by gently shaking the flask and these floating cells were collected and plated on 24- or 96-well plates in DMEM containing 10% FBS. Non-adherent cells were removed after 20–30 min leaving adherent microglial cells that were incubated for 1 h with 100 ng/ml CCL5 before being collected for real-time PCR analysis.

### Flow cytometry

Microglia and endothelial cells were isolated from LPS-injected Cx3cr1-GFP mice and MRL/lpr::Cx3cr1-GFP mice using a collagenase-based digestion medium. The isolated cells were treated with anti-CD16/32 antibody to block Fc receptors (70-0161-U100, TONBO Biosciences, San Diego, CA) before staining. The samples were then processed in PBS solution containing 5% FBS and 2 mM EDTA. The first antibodies against CD31 (14-0311-81, Thermo Fisher Scientific; 1:100) and CLDN5 (35-2500; Thermo Fisher Scientific; 1:100) were incubated for 1 h at 4 °C. Secondary anti-mouse Alexa 405 and anti-rat Alexa 647 (Abcam; 1:500 for each) antibodies were incubated for 30 min at 4 °C. To evaluate the purity of microglial cultures, primary antibodies against CD31 (PE (phycoerythrin) conjugated, 102507, BioLegend; 1:100) and CD11b (APC (allophycocyanin) conjugated, 101211, BioLegend; 1:100) were used. Stained cells were acquired using FACS Aria III or FACS CantoII (BD Biosciences, San Jose, CA), and the data were analyzed using FlowJo software (BD Biosciences). Compensation for spectral overlap between Alexa 405, GFP, PE, Alexa 647, APC was calculated with FACSDiva software using single-stained samples.

### Real-time PCR

To quantify the expression of Cldn5 in microglia, total RNA was extracted from MACS-isolated microglia. First-strand complementary DNA (cDNA) was synthesized from total RNA using Transcriptor First-Strand cDNA Synthesis Kit (04896866001, Roche Diagnostics, Mannheim, Germany) or SuperScript III (18080093, Thermo Fisher Scientific). Amplification reactions were performed on LightCycler 96 System (Roche Diagnostics) using the FastStart Essential DNA Green Master (06402712001, Roche Diagnostics). Amplification results were analyzed with the LightCycler or StepOnePlus (Thermo Fisher Scientific) using the FastStart Essential DNA Green Master (06402712001, Roche Diagnostics) or Fast SYBR Green Master Mix (Thermo Fisher Scientific). Amplification results were analyzed with the LightCycler or StepOnePlus software and then normalized on the basis of the *Gapdh* mRNA levels in each sample. The primer sequences used for the real-time PCR of target gene (*Cldn5*) were forward: 5′-GCTGGCGCTGGTGGCACTCTTTGT-3′ and reverse: 5′-GGCGAACCAGCAGAGCGGCAC-3′, (*Ccr5*) were forward: 5′-AGACATCCGTTCCCCCTACA-3′ and reverse: 5′-GCAGGGTGCTGACATACCAT-3′ and for *Gapdh* were forward: 5′-AATGCATCCTGCACCACCAAC-3′ and reverse: 5′-TGGATGCAGGGATGATGTTCTG-3′.

### Motor learning task

A modified version of the voluntary forelimb movement task^40^ was performed. Briefly, after 2 days of water restriction, head-restrained mice were trained to pull and hold a lever for 600 ms which would be rewarded by a 4 μl drop of water for 14 consecutive days. Behavioral parameters (such as lever position and reward timing) were recorded using a custom program in LabVIEW (National Instruments, TX, USA). Success rates were calculated in both the early phase (1–4 experimental day) and late phase (11–14 experimental day) of the behavioral task in ICR (WT) and MRL/lpr mice.

### Data analysis and statistics

Data were analyzed using GraphPad Prism 8 statistical software (GraphPad Software Inc., La Jolla, CA). All data are presented as means ± SD. Unpaired *t*-test, ANOVA followed by Tukey’s post-hoc test or Holm–Sidak post-hoc test, and Pearson’s correlation tests were used to test for statistical significance.

### Reporting summary

Further information on research design is available in the Nature Research Reporting Summary linked to this article.

## Supplementary information

Supplementary information

Peer Review

Reporting Summary

Description of Additional Supplementary Files

Supplementary Video 1

Supplementary Video 2

Supplementary Video 3

Supplementary Video 4

## Data Availability

The authors declare that the data supporting the findings of this study are available within the article and its Supplementary Information files.

## Source data

Source Data File

## References

[1] D’Mello C, Swain MG (2014). Liver-brain interactions in inflammatory liver diseases: implications for fatigue and mood disorders. Brain. Behav. Immun..

[2] Yirmiya R, Goshen I (2011). Immune modulation of learning, memory, neural plasticity and neurogenesis. Brain. Behav. Immun..

[3] Annane D, Sharshar T (2015). Cognitive decline after sepsis. Lancet Respir. Med.

[4] Dumitrescu AL (2016). Depression and inflammatory periodontal disease considerations-an interdisciplinary approach. Front. Psychol..

[5] Barnes DE, Yaffe K (2011). The projected effect of risk factor reduction on Alzheimer’s disease prevalence. Lancet Neurol..

[6] Abbott NJ, Patabendige AA, Dolman DE, Yusof SR, Begley DJ (2010). Structure and function of the blood-brain barrier. Neurobiol. Dis..

[7] Obermeier B, Daneman R, Ransohoff RM (2013). Development, maintenance and disruption of the blood-brain barrier. Nat. Med..

[8] Liebner S (2018). Functional morphology of the blood-brain barrier in health and disease. Acta Neuropathol..

[9] Keaney J, Campbell M (2015). The dynamic blood-brain barrier. FEBS J..

[10] Kleinberger G (2017). The FTD-like syndrome causing TREM2 T66M mutation impairs microglia function, brain perfusion, and glucose metabolism. EMBO J..

[11] Parkhurst CN (2013). Microglia promote learning-dependent synapse formation through brain-derived neurotrophic factor. Cell.

[12] Dudvarski Stankovic N, Teodorczyk M, Ploen R, Zipp F, Schmidt MHH (2016). Microglia-blood vessel interactions: a double-edged sword in brain pathologies. Acta Neuropathol..

[13] Varatharaj A, Galea I (2017). The blood-brain barrier in systemic inflammation. Brain. Behav. Immun..

[14] Morris G (2018). Leaky brain in neurological and psychiatric disorders: drivers and consequences. Aust. N.Z. J. Psychiatry.

[15] Zhou H, Lapointe BM, Clark SR, Zbytnuik L, Kubes P (2006). A requirement for microglial TLR4 in leukocyte recruitment into brain in response to lipopolysaccharide. J. Immunol..

[16] Jolivel V (2015). Perivascular microglia promote blood vessel disintegration in the ischemic penumbra. Acta Neuropathol..

[17] Kettenmann H, Hanisch UK, Noda M, Verkhratsky A (2011). Physiology of microglia. Physiol. Rev..

[18] Schafer DP (2012). Microglia sculpt postnatal neural circuits in an activity and complement-dependent manner. Neuron.

[19] Akiyoshi R (2018). Microglia enhance synapse activity to promote local network synchronization. eNeuro.

[20] Bialas AR (2017). Microglia-dependent synapse loss in type I interferon-mediated lupus. Nature.

[21] Bendorius M, Po C, Muller S, Jeltsch-David H (2018). From systemic inflammation to neuroinflammation: the case of neurolupus. Int. J. Mol. Sci..

[22] Brey RL, Sakic B, Szechtman H, Denburg JA (1997). Animal models for nervous system disease in systemic lupus erythematosus. Ann. N. Y. Acad. Sci..

[23] Chen Z (2014). Microglial displacement of inhibitory synapses provides neuroprotection in the adult brain. Nat. Commun..

[24] Kondo S, Kohsaka S, Okabe S (2011). Long-term changes of spine dynamics and microglia after transient peripheral immune response triggered by LPS in vivo. Mol. Brain.

[25] Nimmerjahn A, Kirchhoff F, Helmchen F (2005). Resting microglial cells are highly dynamic surveillants of brain parenchyma in vivo. Science.

[26] Wake H, Moorhouse AJ, Jinno S, Kohsaka S, Nabekura J (2009). Resting microglia directly monitor the functional state of synapses in vivo and determine the fate of ischemic terminals. J. Neurosci..

[27] Nishioku T (2009). Detachment of brain pericytes from the basal lamina is involved in disruption of the blood-brain barrier caused by lipopolysaccharide-induced sepsis in mice. Cell. Mol. Neurobiol..

[28] Tanaka KF (2012). Expanding the repertoire of optogenetically targeted cells with an enhanced gene expression system. Cell Rep..

[29] Guillemin GJ, Brew BJ (2004). Microglia, macrophages, perivascular macrophages, and pericytes: a review of function and identification. J. Leukoc. Biol..

[30] Bechmann I (2001). Immune surveillance of mouse brain perivascular spaces by blood-borne macrophages. Eur. J. Neurosci..

[31] Buttgereit A (2016). Sall1 is a transcriptional regulator defining microglia identity and function. Nat. Immunol..

[32] Jiao H, Wang Z, Liu Y, Wang P, Xue Y (2011). Specific role of tight junction proteins claudin-5, occludin, and ZO-1 of the blood-brain barrier in a focal cerebral ischemic insult. J. Mol. Neurosci..

[33] Nitta T (2003). Size-selective loosening of the blood-brain barrier in claudin-5-deficient mice. J. Cell Biol..

[34] Zotova E (2011). Microglial alterations in human Alzheimer’s disease following Abeta42 immunization. Neuropathol. Appl. Neurobiol..

[35] Singh, V., Mitra, S., Sharma, A. K., Gera, R. & Ghosh, D. Isolation and characterization of microglia from adult mouse brain: selected applications for ex vivo evaluation of immunotoxicological alterations following in vivo xenobiotic exposure. *Chem. Res. Toxicol.* **27**, 895–903 (2014). 10.1021/tx500046k 24754514

[36] Tokizane Kyohei, Konishi Hiroyuki, Makide Kumiko, Kawana Hiroki, Nakamuta Shinichi, Kaibuchi Kozo, Ohwada Tomohiko, Aoki Junken, Kiyama Hiroshi (2017). Phospholipid localization implies microglial morphology and function via Cdc42in vitro. Glia.

[37] Wang AL (2005). Minocycline inhibits LPS-induced retinal microglia activation. Neurochem. Int..

[38] Henry CJ (2008). Minocycline attenuates lipopolysaccharide (LPS)-induced neuroinflammation, sickness behavior, and anhedonia. J. Neuroinflammation.

[39] Hira R (2013). Spatiotemporal dynamics of functional clusters of neurons in the mouse motor cortex during a voluntary movement. J. Neurosci..

[40] Masamizu Y (2014). Two distinct layer-specific dynamics of cortical ensembles during learning of a motor task. Nat. Neurosci..

[41] Davalos D (2005). ATP mediates rapid microglial response to local brain injury in vivo. Nat. Neurosci..

[42] Wyss-Coray T, Mucke L (2002). Inflammation in neurodegenerative disease—a double-edged sword. Neuron.

[43] Neumann H, Kotter MR, Franklin RJ (2009). Debris clearance by microglia: an essential link between degeneration and regeneration. Brain.

[44] Davalos D (2012). Fibrinogen-induced perivascular microglial clustering is required for the development of axonal damage in neuroinflammation. Nat. Commun..

[45] Ryu JK, McLarnon JG (2006). Minocycline or iNOS inhibition block 3-nitrotyrosine increases and blood–brain barrier leakiness in amyloid beta-peptide-injected rat hippocampus. Exp. Neurol..

[46] Yenari MA, Xu L, Tang XN, Qiao Y, Giffard RG (2006). Microglia potentiate damage to blood-brain barrier constituents. Stroke.

[47] Yang Y (2015). Attenuation of acute stroke injury in rat brain by minocycline promotes blood–brain barrier remodeling and alternative microglia/macrophage activation during recovery. J. Neuroinflammation.

[48] Yrjänheikki J (1999). A tetracycline derivative, minocycline, reduces inflammation and protects against focal cerebral ischemia with a wide therapeutic window. Proc. Natl Acad. Sci. USA.

[49] Yang Y (2015). Attenuation of acute stroke injury in rat brain by minocycline promotes blood-brain barrier remodeling and alternative microglia/macrophage activation during recovery. J. Neuroinflammation.

[50] Hanisch UK, Kettenmann H (2007). Microglia: active sensor and versatile effector cells in the normal and pathologic brain. Nat. Neurosci..

[51] Horng S (2017). Astrocytic tight junctions control inflammatory CNS lesion pathogenesis. J. Clin. Invest..

[52] McConnell HL, Li Z, Woltjer RL, Mishra A (2019). Astrocyte dysfunction and neurovascular impairment in neurological disorders: correlation or causation?. Neurochem. Int..

[53] da Fonseca AC (2014). The impact of microglial activation on blood-brain barrier in brain diseases. Front. Cell Neurosci..

[54] Zhang Y (2014). An RNA-sequencing transcriptome and splicing database of glia, neurons, and vascular cells of the cerebral cortex. J. Neurosci..

[55] Li Q (2019). Developmental heterogeneity of microglia and brain myeloid cells revealed by deep single-cell RNA sequencing. Neuron.

[56] McKim DB (2017). Microglial recruitment of IL-1β-producing monocytes to brain endothelium causes stress-induced anxiety. Mol. Psychiatry.

[57] Zenaro E, Piacentino G, Constantin G (2017). The blood-brain barrier in Alzheimer’s disease. Neurobiol. Dis..

[58] McKim DB (2018). Microglial recruitment of IL-1beta-producing monocytes to brain endothelium causes stress-induced anxiety. Mol. Psychiatry.

[59] Abbott NJ, Ronnback L, Hansson E (2006). Astrocyte-endothelial interactions at the blood-brain barrier. Nat. Rev. Neurosci..

[60] Jung S (2000). Analysis of fractalkine receptor CX3CR1 function by targeted deletion and green fluorescent protein reporter gene insertion. Mol. Cell. Biol..

[61] Stanger BZ, Tanaka AJ, Melton DA (2007). Organ size is limited by the number of embryonic progenitor cells in the pancreas but not the liver. Nature.

[62] Di Prisco S, Summa M, Chellakudam V, Rossi PIA, Pittaluga A (2012). RANTES-mediated control of excitatory amino acid release in mouse spinal cord. J. Neurochem..

[63] Rosi S, Pert CB, Ruff MR, McGann-Gramling K, Wenk GL (2005). Chemokine receptor 5 antagonist d-Ala-peptide T-amide reduces microglia and astrocyte activation within the hippocampus in a neuroinflammatory rat model of Alzheimer’s disease. Neuroscience.

[64] Nikolakopoulou AM, Zhao Z, Montagne A, Zlokovic BV (2017). Regional early and progressive loss of brain pericytes but not vascular smooth muscle cells in adult mice with disrupted platelet-derived growth factor receptor-β signaling. PLoS One.

[65] Thevenaz P, Ruttimann UE, Unser M (1998). A pyramid approach to subpixel registration based on intensity. IEEE Trans. Image Process.

[66] Bell RD (2010). Pericytes control key neurovascular functions and neuronal phenotype in the adult brain and during brain aging. Neuron.

[67] Zhou Y (2019). Metascape provides a biologist-oriented resource for the analysis of systems-level datasets. Nat. Commun..

[68] Katoh M (2017). Polymorphic regulation of mitochondrial fission and fusion modifies phenotypes of microglia in neuroinflammation. Sci. Rep..

[69] Parajuli LK (2012). Quantitative regional and ultrastructural localization of the Ca(v)2.3 subunit of R-type calcium channel in mouse brain. J. Neurosci..

[70] Nguyen HB (2016). Conductive resins improve charging and resolution of acquired images in electron microscopic volume imaging. Sci. Rep..

[71] Alan, P., Sanford, L. P. & Henry, deF. The Fine Structure of the Nervous System: *The Neurons and Supporting Cells.* (Oxford University Press, 1991).

[72] Ruck, T., Bittner, S., Epping, L., Herrmann, A. M. & Meuth, S. G. Isolation of primary murine brain microvascular endothelial cells. *J. Vis. Exp*. e52204–e52204 (2014). 10.3791/52204 PMC4354020 25489873

[73] Assmann, J. C. et al. Isolation and cultivation of primary brain endothelial cells from adult mice. *Bio-Protocol* **7**, e2294 (2017). 10.21769/BioProtoc.2294 PMC5464392 28603749

