# Supplementary material for: Dual microglia effects on blood brain barrier permeability induced by systemic inflammation
Source: Nat Commun. 2019 Dec 20;10:5816. doi: 10.1038/s41467-019-13812-z (PMC6925219; doi:10.1038/s41467-019-13812-z)
Supplement: Supplementary file 1 — Supplementary information [file 41467_2019_13812_MOESM1_ESM.pdf]

Supplementary Information

Dual Microglia Effects on Blood Brain Barrier Permeability Induced by Systemic Inflammation

Haruwaka et al.

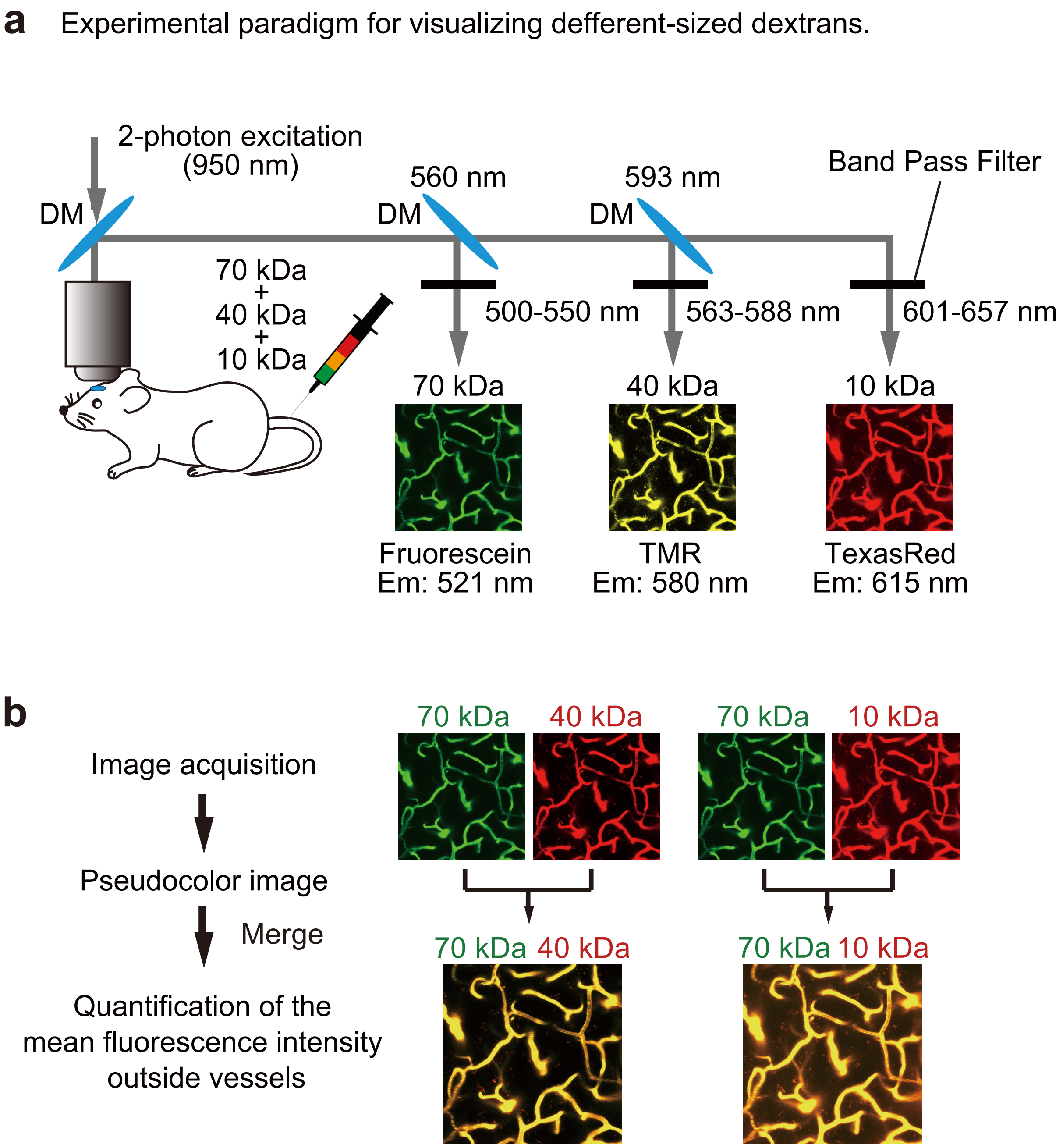

Supplementary Figure 1

Experimental protocol for the quantification of BBB permeability. (a) Visualization of three different-sized dextrans. We intravenously injected a cocktail of the following dextrans: 70 kDa (Fluorescein, green), 40 kDa (Tetramethylrhodamine [TMR], yellow), and 10 kDa (TexasRed, red). The fluorescence signals were split into green, yellow, and red channels using dichroic mirrors (560 nm and 593 nm) and bandpass filtered (green, 500-550 nm; yellow, 563-588 nm; red, 601-657 nm). (b) Image processing method for the quantification of dextran leakage, linked to Fig. 1d. The original images of 70 kDa and 40 kDa dextrans were pseudo-colored with green and red, and then merged to delineate vessels and parenchymal fluorescence arising from BBB leak. The same procedure was applied to the images of 70 kDa and 10 kDa dextrans pair.

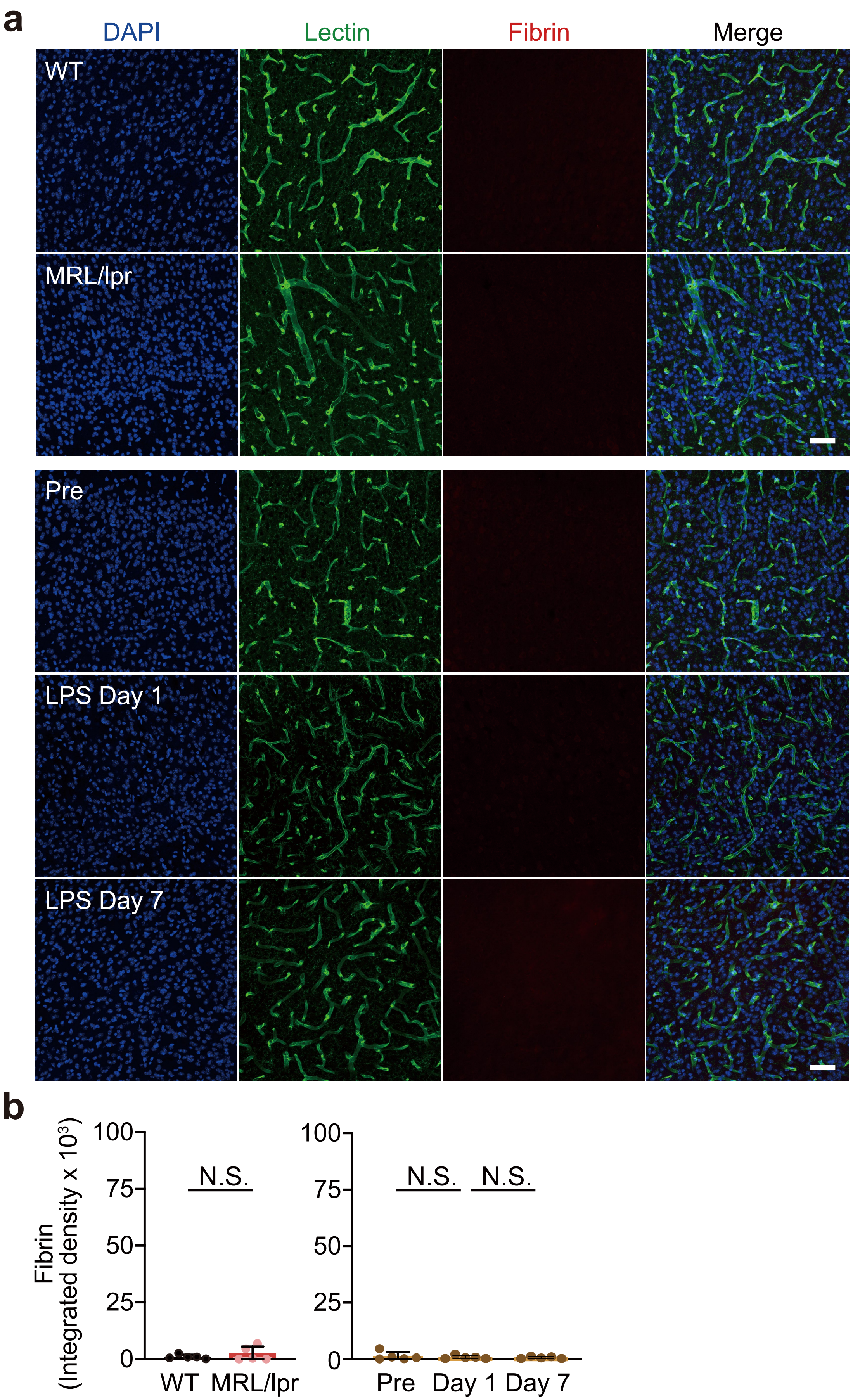
a
DAPI
Lectin
Fibrin
Merge
WT
MRL/lpr
Pre
LPS Day 1
LPS Day 7
b
Fibrin
(Integrated density x 10³)
100
75
50
25
0
N.S.
WT
MRL/lpr
N.S.
N.S.
Pre
Day 1
Day 7

Supplementary Figure 2

Immunostaining of fibrin to quantify BBB leakage. (a) Typical immunohistochemical images showing lectin (green), fibrin (red), and DAPI (blue). Scale bar 50 µm. (b) Quantification of fibrinogen extravascular leakage. Each point represents data from a single animal, while columns and error bars show mean ± SD. N.S., not significant.

Supplementary Figure 3

Haruwaka et al.

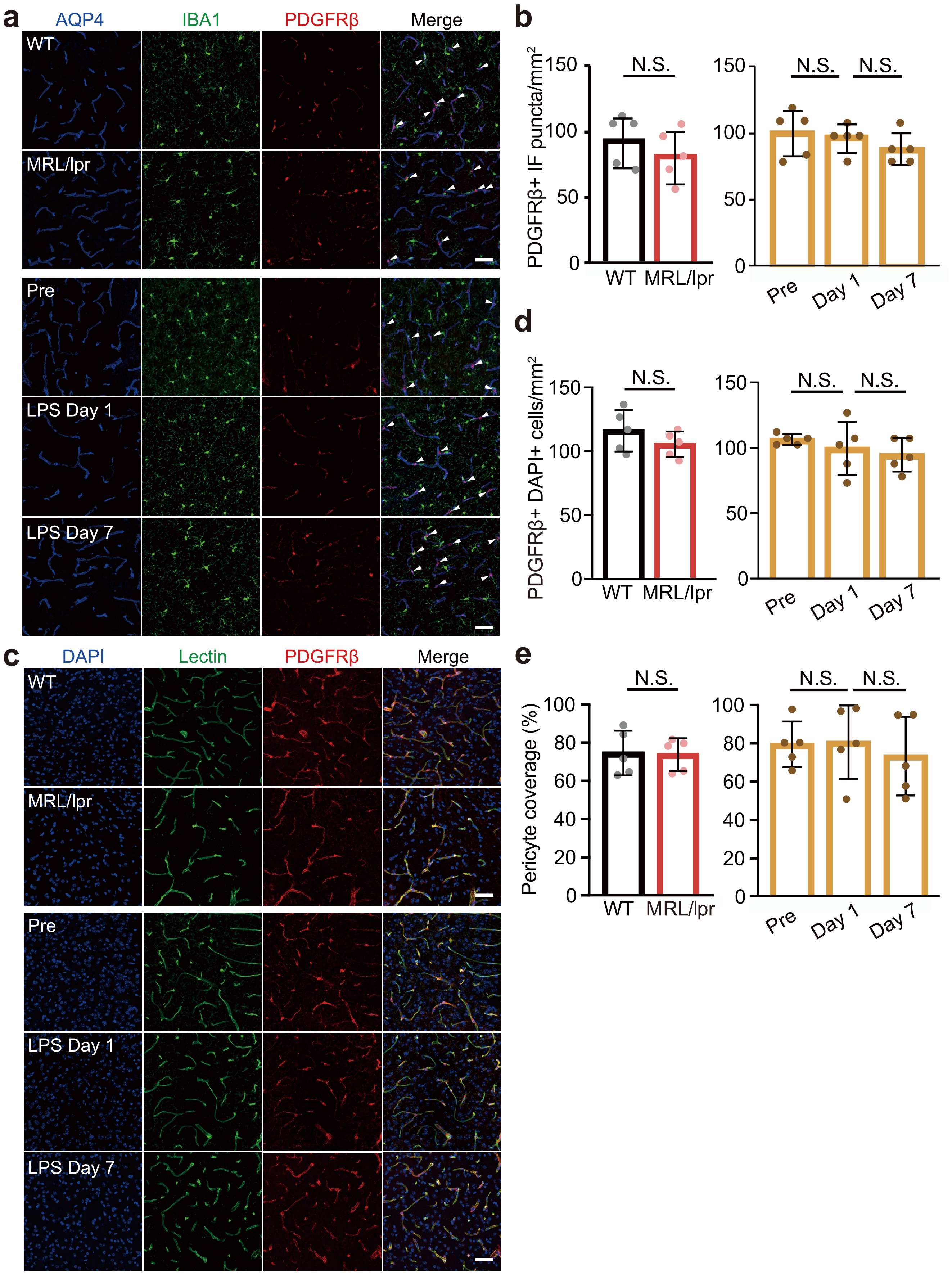

Supplementary Figure 3

Pericyte number and pericyte coverage in MRL/lpr and LPS injection model. Pericytes were identified by PDGFRβ fluorescence (red). (a) Typical immunohistochemical images showing cells or processes (defined here as puncta) positive for PDGFRβ (red, pericyte marker), AQP4 (astrocytic end-feet marker, used to identify vessels; blue) and IBA1 (microglia, green). Arrowheads in the merged fluorescence image indicate pericytes associated with vessels, as used to quantify pericyte density as shown in (b) Scale bar 50 μm. (b) The graph showed no significant difference in pericyte immunofluorescence (IF) puncta density between WT and MRL/lpr mice (n = 5 mice in each group), nor between pre and post LPS injected mice (Day 1 and Day 7; n = 5 mice in each group). (c) Typical immunohistochemical images showing cells positive for PDGFRβ (red, pericyte marker), Lectin (used to identify vessels; green) and DAPI (nuclei, blue) as used to quantify PDGFRβ positive and DAPI positive cell and pericyte coverage as shown in (d) and (e). (d) The graph showed no significant difference in the density of PDGFRβ positive and DAPI positive cell between WT and MRL/lpr mice (n = 5 mice in each group), nor between pre and post LPS injected mice (Day 1 and Day 7; n = 5 mice in each group). (e) The graph showed no significant difference in the pericyte coverage between WT and MRL/lpr mice (n = 5 mice in each group), nor between pre and post LPS injected mice (Day 1 and Day 7; n = 5 mice in each group). Each point represents data from a single field (b, c), while columns and error bars show mean ± SD. N.S., not significant.

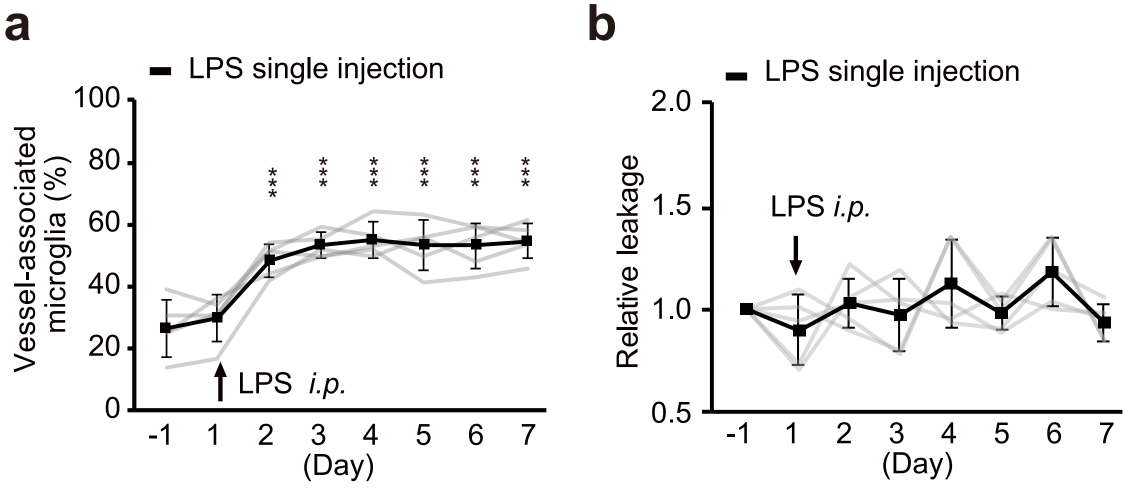

Supplementary Figure 4

The effect of a single LPS injection. The effect of a single LPS injection on (a) The percentage of vessel-associated microglia and (b) The permeability of the BBB, as measured by Dextran (10 kDa) leak into the brain parenchyma. A single LPS injection was sufficient to induce a sustained increase in microglia migration to vessels, but did not affect BBB permeability. In each graph the faint lines indicate data from an individual animal (a, b) while the dark lines and error bars indicate the mean ± SD. ***$P < 0.001$.

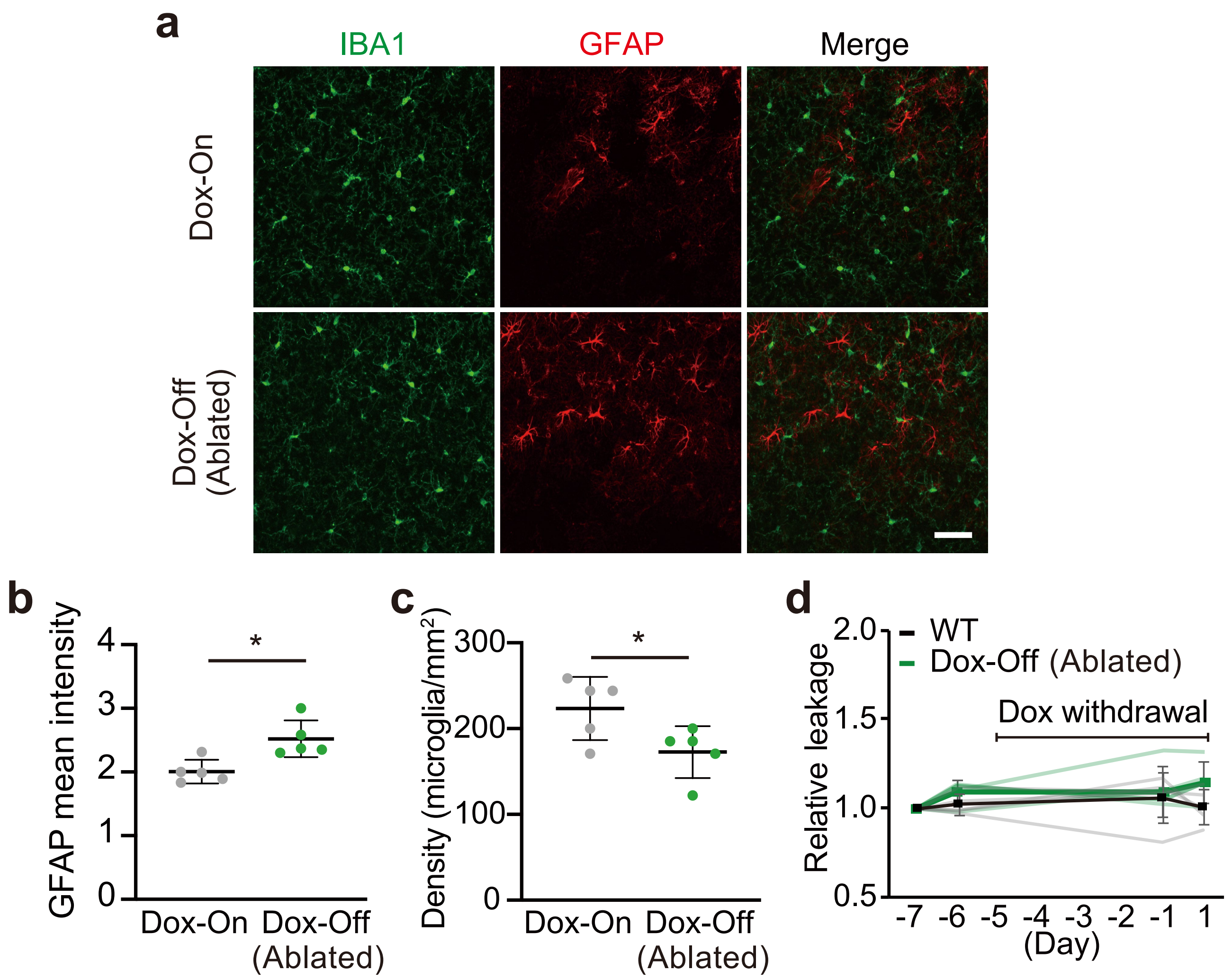

Supplementary Figure 5

Microglia ablation stimulates modest astrocyte activation or proliferation. (a) Typical immunohistochemical images of microglia (IBA1, left, green in merged image), astrocytes (GFAP, center, red in merged image). Partial microglia ablation was achieved by withdrawing Doxycycline from the diet of Iba1-tTA::tetO-DTA mice (Dox-Off). Scale bar, 50 µm. (b) The mean GFAP intensity across an image field was increased in microglia ablated mice, suggesting slight activation of astrocytes. (c) The density of microglia was modestly reduced in microglia ablated mice (Dox-Off) as compared to control (Dox-On) mice. (d) A plot of the relative permeability of the BBB, as measured by Dextran (10 kDa) leak into the brain parenchyma. There was no significant effect of Dox withdrawal on BBB permeability. Each point in graphs (b) and (c), and the faint lines in graph (d), indicate data from a single field, while the columns (b, c) or dark lines (d), and error bars show mean ± SD. *$P < 0.05$.

Supplementary Figure 6

Haruwaka et al.

a

Microglia

Astrocyte

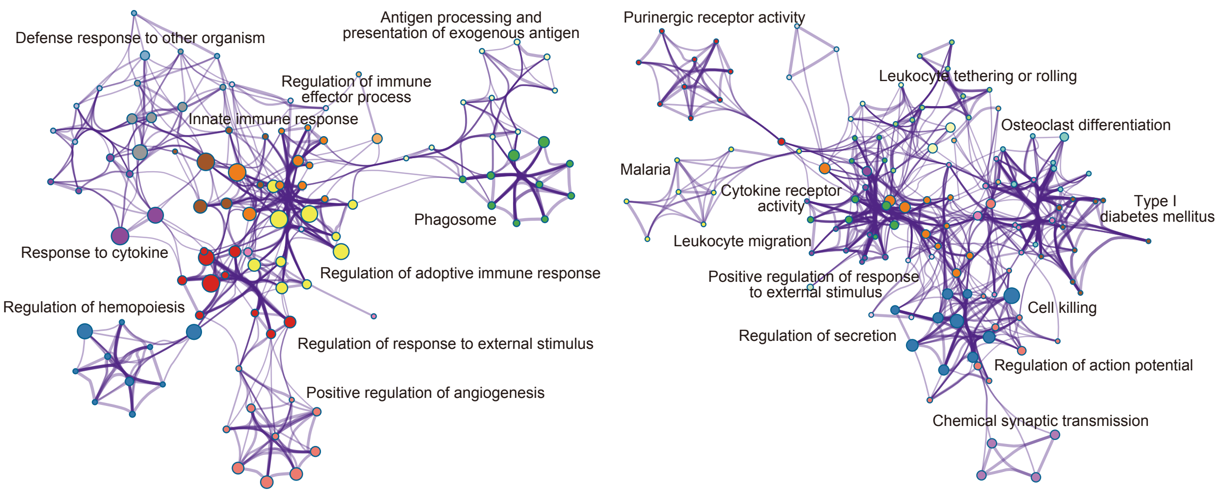

b

Microglia

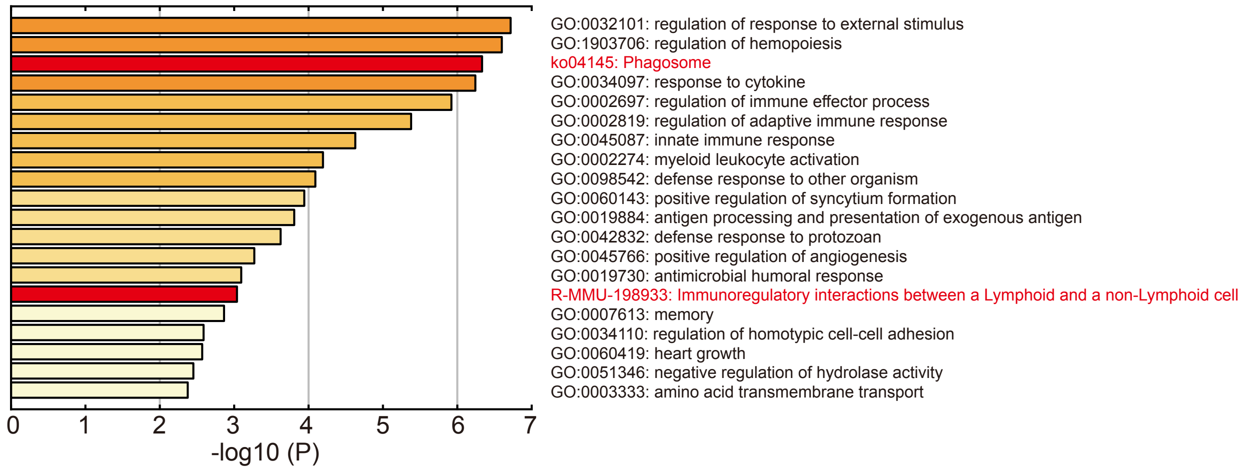

c

Astrocyte

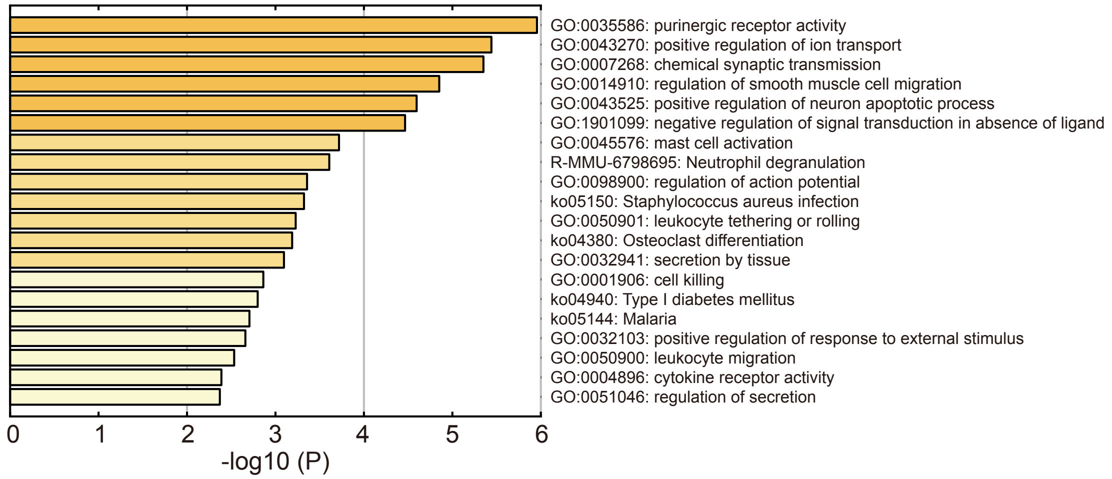

Supplementary Figure 6

Microglia and astrocyte RNA gene microarrays in MRL/lpr mice. (a) Map of functional clusters of genes upregulated in microglia and astrocyte from MRL/lpr mice. (b, c) Graph of functional gene clusters showing the extent of upregulation in functional families from microglia (b) and astrocytes (c) from MRL/lpr mice, as compared to WT mice.

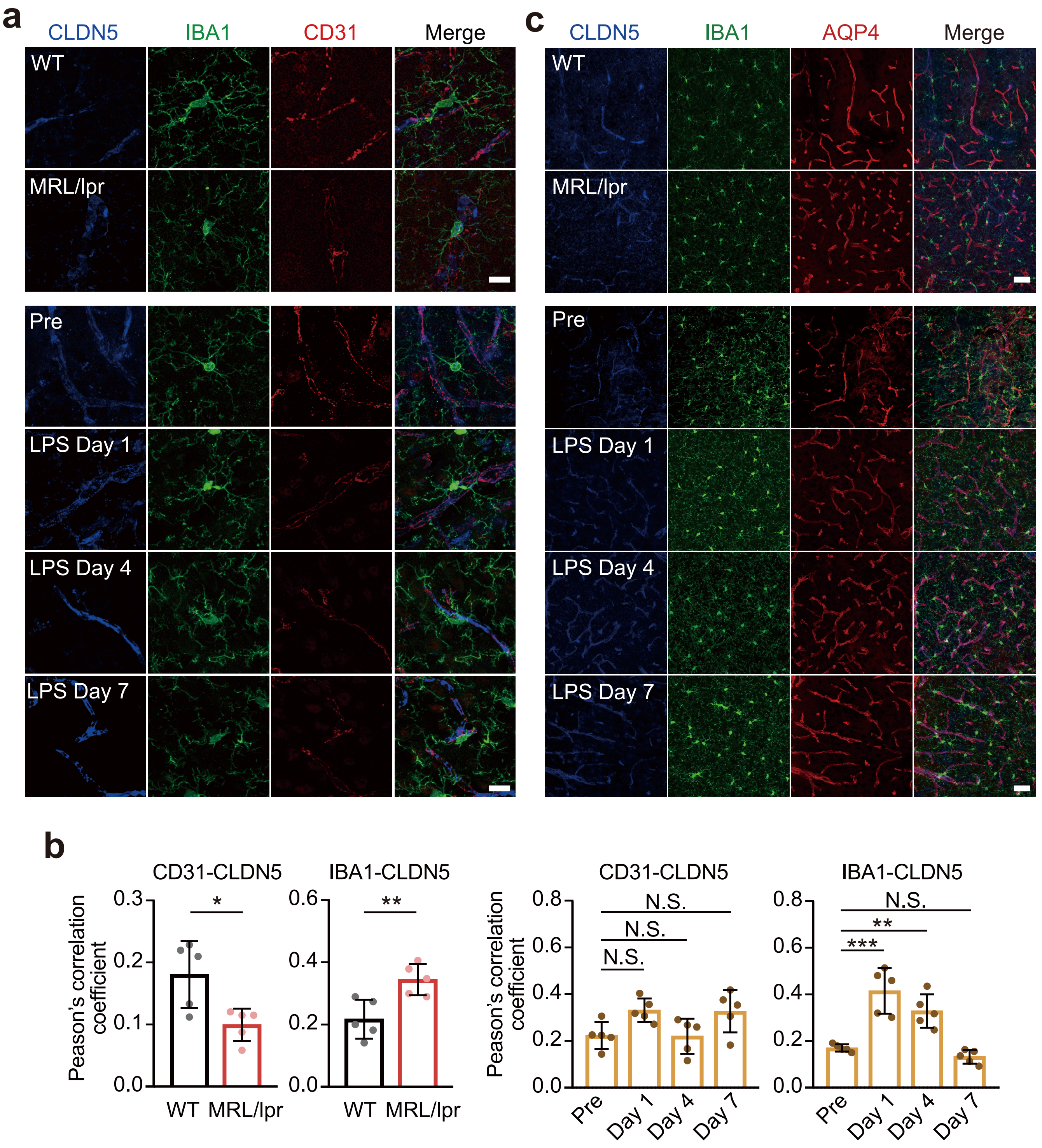
a
CLDN5
IBA1
CD31
Merge
WT
MRL/lpr
Pre
LPS Day 1
LPS Day 4
LPS Day 7
c
CLDN5
IBA1
AQP4
Merge
WT
MRL/lpr
Pre
LPS Day 1
LPS Day 4
LPS Day 7
b
CD31-CLDN5
IBA1-CLDN5
Peason's correlation coefficient
0.3
0.2
0.1
0.0
0.6
0.4
0.2
0.0
*
**
WT MRL/lpr
WT MRL/lpr
CD31-CLDN5
IBA1-CLDN5
0.8
0.6
0.4
0.2
0.0
N.S.
N.S.
N.S.
***
**
Pre
Day 1
Day 4
Day 7

Supplementary Figure 7

CLDN5 expression in microglia and endothelial cells. (a) Typical immunohistochemical images of microglia (IBA1, green) in WT, MRL/lpr and LPS injected mice, co-stained with fluorescent markers for endothelial cells (CD31, red) and the tight junction protein (CLDN5, blue). (b) The extent of colocalization of CLDN5 and CD31 decreased in MRL/lpr mice, but IBA1 and CLDN5 co-localization increased (left panels). The extent of colocalization of CLDN5 and CD31 was not affected by LPS injection, but IBA1 and CLDN5 co-localization increased at 1 and 4 days after LPS injection (right panels). (c) Typical immunohistochemical images of microglia (IBA1, green) in WT, MRL/lpr and LPS injected mice, co-stained with fluorescent markers for astrocytic end-feet (AQP4, red), and the tight junction protein (CLDN5, blue). The column of panels shown in (c) represent the individual fluorescent channels that are merged in Figure 4a, c. Scale bar, 50 μm. N.S., not significant, *, $P<0.05$, ** $P<0.01$ and *** $P<0.001$.

Supplementary Figure 8

Haruwaka et al.

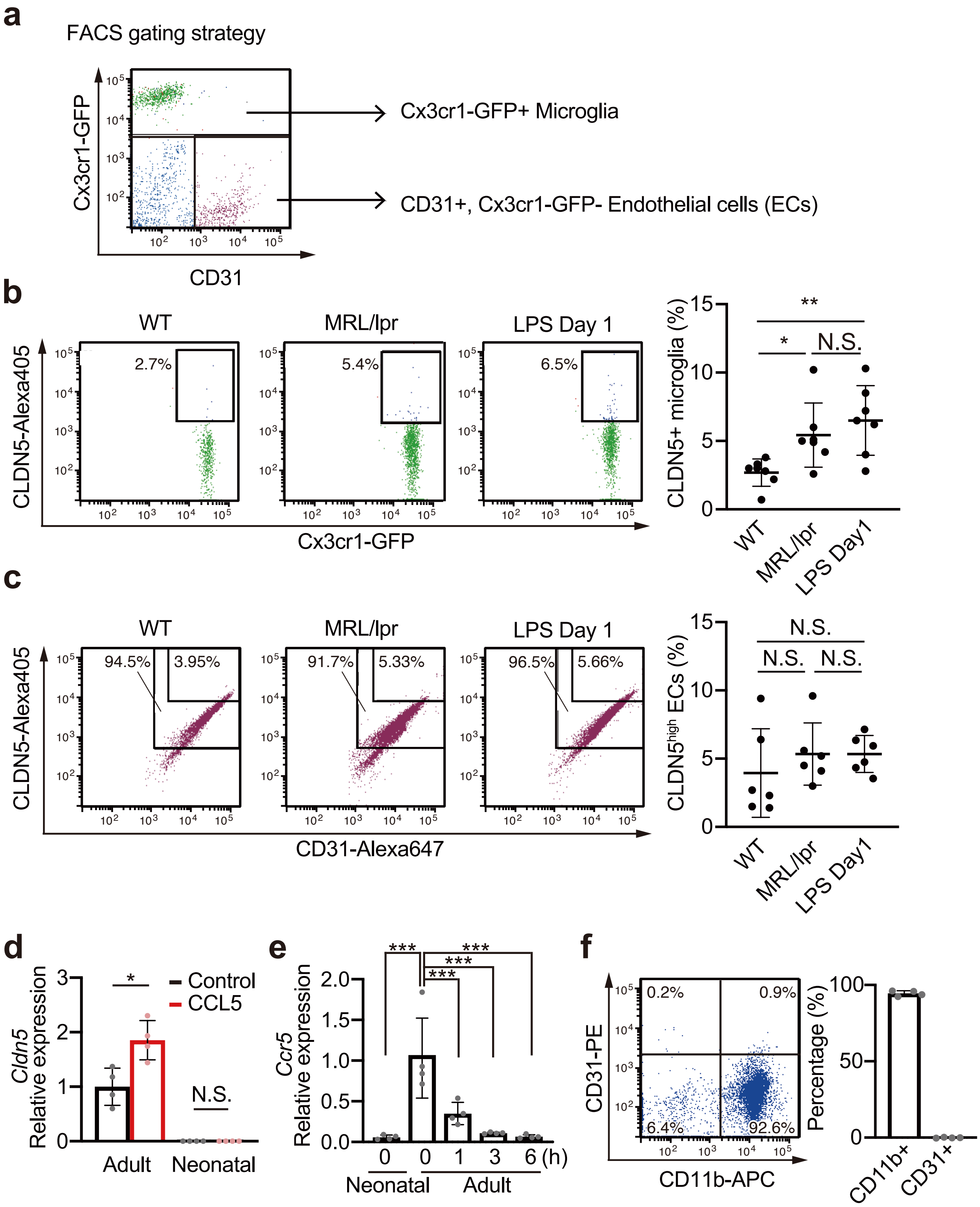

Supplementary Figure 8

FACS separation of microglia and astrocytes to quantify CLDN5 expression. (a) FACS gating strategy to separate endothelial cells by high relative CD31 +' ve fluorescence, and microglia by high relative CX3CR1 +' ve fluorescence. (b, c) Representative data images for FACS analysis of CLDN5 +' ve immune fluorescence in microglia (b) and endothelial cell (c) populations. The accompanying graphs showed that CLDN5 expression is increased in microglia from MRL/lpr mice and following a single LPS injection (b), but is not significantly different in endothelial cells (c). (d) CCL5 stimulation on adult and neonatal microglial culture. Cldn5 mRNA expression levels increased in adult microglial culture, while that increase was absent in neonatal mice. (e) Ccr5 mRNA expression in in vitro culture with the time course 0, 1, 3, 6 hours after microglial culture. 0 h adult culture showed higher expression of Ccr5 that is consistent with the increase Cldn5 expression. (f) Purity of CD11b +' ve microglia verified by flow cytometric analysis. Each point represents data from a single analysis experiment (b, c, d, e), while columns and error bars show mean ± SD. N.S., not significant, *, $P<0.05$, ** $P<0.01$ and *** $P<0.001$.

Supplementary Figure 9 Haruwaka et al.

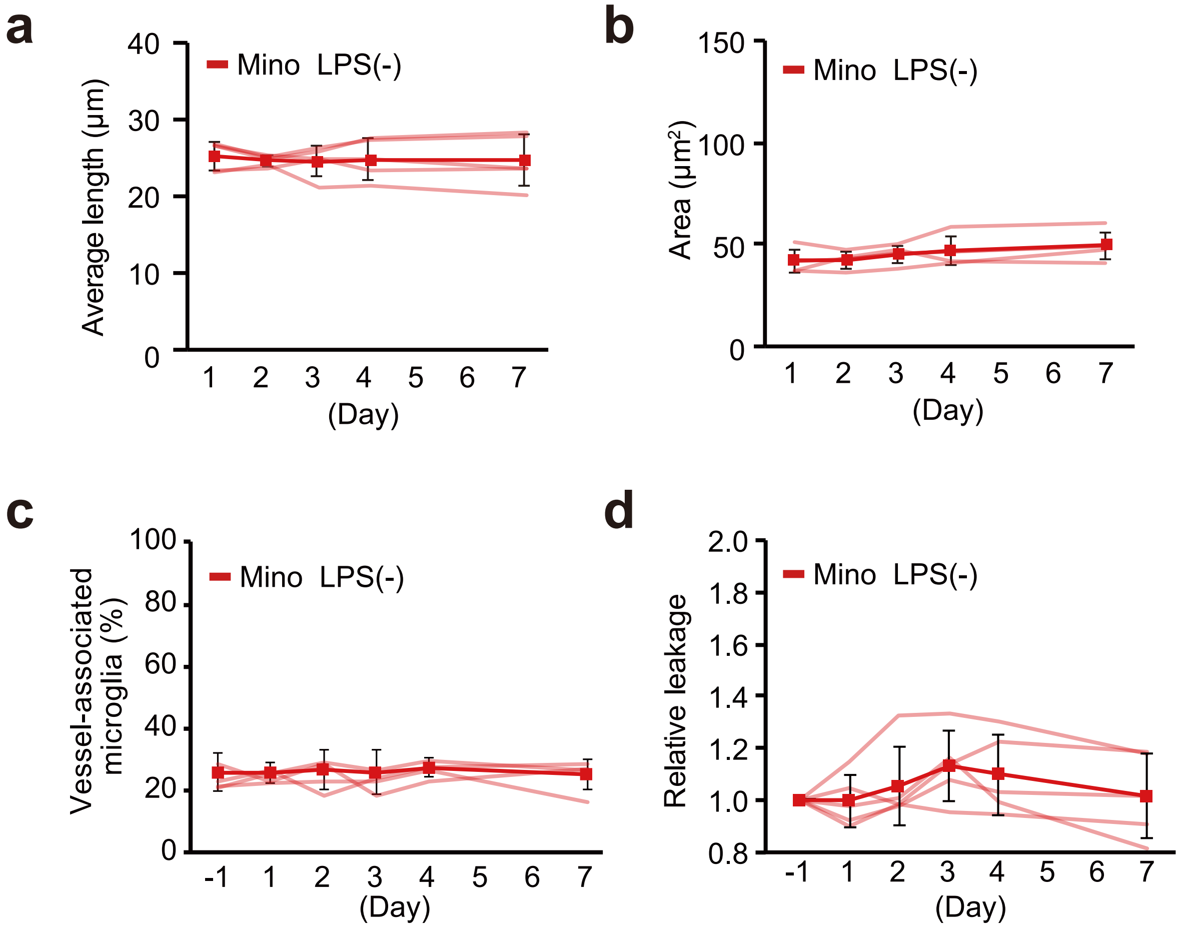

Supplementary Figure 9

Minocycline by itself does not affect microglia migration and BBB permeability but reduces microglia phenotypic and BBB permeability associated with LPS. (a-b) Graphs plotting the change in microglia average process length (a) and soma area (b) in mice treated with minocycline (75 mg/kg, i.p.), and with or without daily LPS injections from Day 1. (c-d) Graphs plotting the proportion of microglia associated with vessels (c) and the relative leak of 10 kDa dextran across the BBB (d) in mice treated with minocycline as above, and with or without daily LPS injections from Day 1.

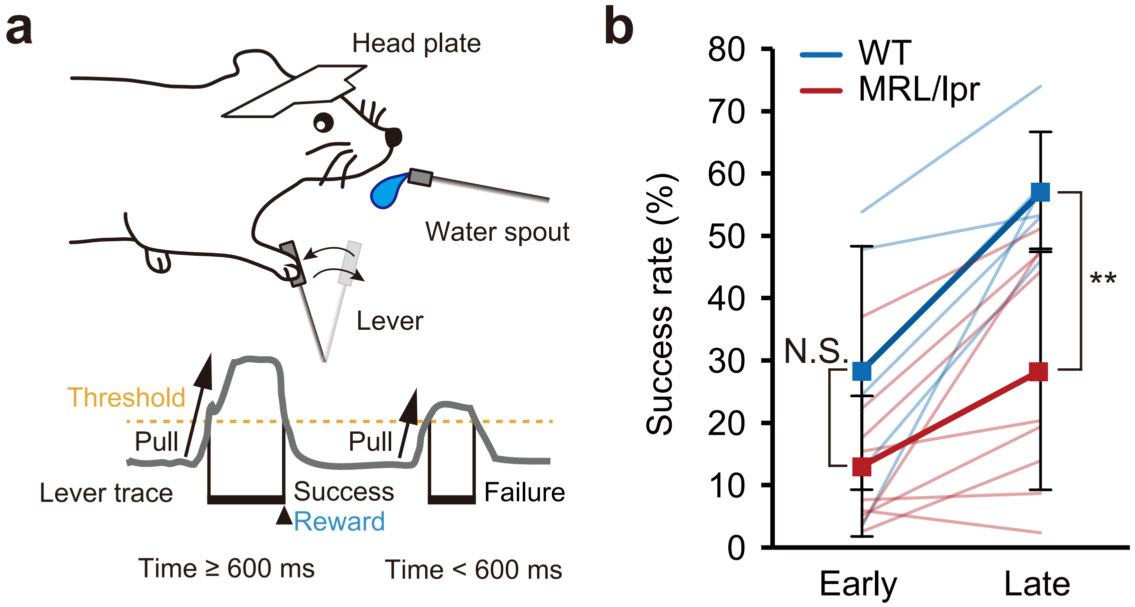

Supplementary Figure 10

Motor learning is impaired in MRL/lpr mice. Graph plotting the proportion of lever pulling tasks that successfully triggered a water delivery response to thirsty mice, during the initial daily training period (1-4 days, Early) and once mice have been further trained (11-14 days, Late). The proportion of successful attempts in the late phase was significantly lower in MRL/lpr mice as compared with that of the control mice. Each connected faint line represents data from a single mouse, while the dark lines and error bars show mean ± SD. N.S. not significant, ** $P < 0.01$.
