# Supplementary material for: Dual microglia effects on blood brain barrier permeability induced by systemic inflammation
Source: Nat Commun. 2019 Dec 20;10:5816. doi: 10.1038/s41467-019-13812-z (PMC6925219; doi:10.1038/s41467-019-13812-z)
Supplement: Supplementary file 3 — Reporting Summary [file 41467_2019_13812_MOESM3_ESM.pdf]

nature research

Corresponding author(s): Hiroaki Wake

Last updated by author(s): Nov 19, 2019

# Reporting Summary

Nature Research wishes to improve the reproducibility of the work that we publish. This form provides structure for consistency and transparency in reporting. For further information on Nature Research policies, see Authors & Referees and the Editorial Policy Checklist.

## Statistics

For all statistical analyses, confirm that the following items are present in the figure legend, table legend, main text, or Methods section.

| n/a | Confirmed | |
|---|---|---|
| ☐ | ☒ | The exact sample size ($n$) for each experimental group/condition, given as a discrete number and unit of measurement |
| ☐ | ☒ | A statement on whether measurements were taken from distinct samples or whether the same sample was measured repeatedly |
| ☐ | ☒ | The statistical test(s) used AND whether they are one- or two-sided<br>*Only common tests should be described solely by name; describe more complex techniques in the Methods section.* |
| ☒ | ☐ | A description of all covariates tested |
| ☐ | ☒ | A description of any assumptions or corrections, such as tests of normality and adjustment for multiple comparisons |
| ☐ | ☒ | A full description of the statistical parameters including central tendency (e.g. means) or other basic estimates (e.g. regression coefficient) AND variation (e.g. standard deviation) or associated estimates of uncertainty (e.g. confidence intervals) |
| ☐ | ☒ | For null hypothesis testing, the test statistic (e.g. $F$, $t$, $r$) with confidence intervals, effect sizes, degrees of freedom and $P$ value noted<br>*Give P values as exact values whenever suitable.* |
| ☒ | ☐ | For Bayesian analysis, information on the choice of priors and Markov chain Monte Carlo settings |
| ☒ | ☐ | For hierarchical and complex designs, identification of the appropriate level for tests and full reporting of outcomes |
| ☐ | ☒ | Estimates of effect sizes (e.g. Cohen's $d$, Pearson's $r$), indicating how they were calculated |

*Our web collection on statistics for biologists contains articles on many of the points above.*

## Software and code

Policy information about availability of computer code

| | |
|---|---|
| Data collection | For two-photon imaging and confocal imaging analysis ZEN (Carl Zeiss) and NIS-Elements (Nikon Instech Co., Ltd.) was used. |
| Data analysis | Image J (Fiji version 1.51n), MATLAB R2017b, GraphPad Prism 8.01 |

For manuscripts utilizing custom algorithms or software that are central to the research but not yet described in published literature, software must be made available to editors/reviewers. We strongly encourage code deposition in a community repository (e.g. GitHub). See the Nature Research guidelines for submitting code & software for further information.

## Data

Policy information about availability of data

All manuscripts must include a data availability statement. This statement should provide the following information, where applicable:
- Accession codes, unique identifiers, or web links for publicly available datasets
- A list of figures that have associated raw data
- A description of any restrictions on data availability

No restrictions for data availability. The associated raw data are available for all figures: Figure 1-7, Supplementary Figure 10 and Supplementary Video 1-4.

# Field-specific reporting

Please select the one below that is the best fit for your research. If you are not sure, read the appropriate sections before making your selection.

☒ Life sciences ☐ Behavioural & social sciences ☐ Ecological, evolutionary & environmental sciences

For a reference copy of the document with all sections, see nature.com/documents/nr-reporting-summary-flat.pdf

# Life sciences study design

All studies must disclose on these points even when the disclosure is negative.

| | |
|---|---|
| Sample size | No calculation was performed to pre-determine sample sizes. The sample size was estimated based on previous reports.<br><br>For in vivo imaging, sample size was n = 5 mice [control, LPS injection model (single injection), LPS injection + DAPTA model, IFNa injection model, IFNa + DAPTA injection model, Minocycline with LPS, vehicle with LPS, and Minocycline without LPS], n = 6 mice [LPS injection model (multiple daily injection)], n = 6 mice [Dox-On] and n = 6 mice [Dox-Off].<br><br>For immunohistochemistry, the sample size was more than n = 4 mice per condition.<br>At least five microglia were analyzed in each animal. |
| Data exclusions | There was no data exclusion. |
| Replication | Experimental data in vivo imaging was confirmed/supported by the validation of immunohistochemistry data. All replications were successful. |
| Randomization | Randomization was not used. |
| Blinding | Blinding was not used. |

# Reporting for specific materials, systems and methods

We require information from authors about some types of materials, experimental systems and methods used in many studies. Here, indicate whether each material, system or method listed is relevant to your study. If you are not sure if a list item applies to your research, read the appropriate section before selecting a response.

## Materials & experimental systems

| n/a | Involved in the study | |
|---|---|---|
| ☐ | ☒ | Antibodies |
| ☒ | ☐ | Eukaryotic cell lines |
| ☒ | ☐ | Palaeontology |
| ☐ | ☒ | Animals and other organisms |
| ☒ | ☐ | Human research participants |
| ☒ | ☐ | Clinical data |

## Methods

| n/a | Involved in the study | |
|---|---|---|
| ☒ | ☐ | ChIP-seq |
| ☐ | ☒ | Flow cytometry |
| ☒ | ☐ | MRI-based neuroimaging |

## Antibodies

| | |
|---|---|
| Antibodies used | For immunohistochemistry, the antibodies description is the following:<br>- anti-Iba1 from Abcam, ab5076 and Lot number GR3178788-2; dilution 1:400<br>- anti-Aqp4 from Merck KGaA, 3072342 and Lot number GR3178788-2; dilution 1:500<br>- anti-Cldn5 from Thermo Fisher Scientific, 35-2500 and Lot number TD259088; dilution 1:100<br>- anti-CD68 from Bio-Rad, MCA1957GA and Lot number 1708; dilution dilution 1:400<br>- anti-PDGFRb from Thermo Fisher Scientific, 14-1402-82 and Lot number 1928809,dilution 1:100<br>- anti-TMEM119 from Synaptic Systems, 400-011 and Lot number 1-2; dilution1:100<br>- anti-CD31 from Abcam, ab28364 and Lot number GR3247742-8; dilution 1:100)<br>- anti-GFAP from Abcam, ab53554-100 and Lot number GR3221771-3, dilution, 1:400<br>- anti-Fibrin from DAKO, A0080 and Lot number 20061286, dilution, 1:250 |
| Validation | All antibodies used in this study have been tested by the company and have been cited by other authors. The related references are available on the webpage of the provider company. In addition, regarding antibodies used in immunohistochemistry, we have further evaluated the specificity of the antibodies in our tissue by analyzing the presence of the antibody signals in regions where the protein should be expressed and its absence in regions where the protein shouldn't be expressed (for instance Cldn5 cannot be expressed in controls parenchymal microglia and indeed it was not). We have further evaluated the location/morphology of the signal within the cells. For Aqp4 and Cldn5, the antibody signal was indeed detected on vessels as expected. |

## Animals and other organisms

Policy information about studies involving animals; ARRIVE guidelines recommended for reporting animal research

| | |
|---|---|
| Laboratory animals | Mouse lines used in this study included ICR wild type (WT) mice, MRL/MpJJmsSlc-lpr/lpr (MRL/lpr) mice, C57BL/6J mice, Cx3cr1-EGFP mice, Sall1-GFP mice and Iba1-tTA::tetO-DTA mice (Iba1-tetracycline transactivator tetracycline operator::diphtheria toxin A mice). Iba1-tTA::tetO-DTA mice are a strain of mice obtained originally by crossing mice with tTA under the control of a Iba1 promoter (a C57BL/6J genetic background) with tetO-DTA mice (a C57BL/6J genetic background) to ablate microglia. All male mice were used for experiments between 7-12 weeks of age. |
| Wild animals | The study did not involve wild animals. |
| Field-collected samples | The study did not involve samples collected from the field. |
| Ethics oversight | The experimental protocols were approved by the Animal Care and Use Committees of Kobe University Graduate School of Medicine and National Institutes of Natural Sciences, and were conducted according to the guidelines of the National Institutes of Health Guide for the Care and Use of Laboratory Animals. |

Note that full information on the approval of the study protocol must also be provided in the manuscript.

## Flow Cytometry

### Plots

Confirm that:

- [x] The axis labels state the marker and fluorochrome used (e.g. CD4-FITC).
- [x] The axis scales are clearly visible. Include numbers along axes only for bottom left plot of group (a 'group' is an analysis of identical markers).
- [ ] All plots are contour plots with outliers or pseudocolor plots.
- [x] A numerical value for number of cells or percentage (with statistics) is provided.

### Methodology

| | |
|---|---|
| Sample preparation | This information is included in the Methods section page. |
| Instrument | BD FACSAria III, FACS CantoII |
| Software | BD FACSDiva v8.0.1 and FlowJo |
| Cell population abundance | Cell sorting not employed |
| Gating strategy | Fig. S6a-c: Gates were made for live cells (FSC-A by SSC-A), then singlets (FSC-W by FSC-A) for all samples. Debris was removed by gating on the main cell population. Microglia were gated by high GFP signal from Cx3cr1-GFP mice. For endothelial cells, high CD31 signal were quantified to measure percentage of Cldn5 positive cells.<br>Fig. S6f: Gates were made for live cells (FSC-A by SSC-A), then singlets (FSC-W by FSC-A) for all samples. Debris was removed by gating on the main cell population. Microglia were gated by CD11b signal. Endothelial cells were gated by CD31 signal. |

- [x] Tick this box to confirm that a figure exemplifying the gating strategy is provided in the Supplementary Information.
