# Supplementary material for: Dual microglia effects on blood brain barrier permeability induced by systemic inflammation
Source: Nat Commun. 2019 Dec 20;10:5816. doi: 10.1038/s41467-019-13812-z (PMC6925219; doi:10.1038/s41467-019-13812-z)
Supplement: Supplementary file 4 — Description of Additional Supplementary Files [file 41467_2019_13812_MOESM4_ESM.docx]

**Description of Additional Supplementary Files**

File name: Supplementary Movie 1

Description: Sample movie montage compiled from daily imaging (1 frame/day) of microglia (green) and blood vessels (red) shows the typical time course of microglia migration *in vivo* before and after induction of systemic inflammation. LPS injection triggered microglia migration to the vessels.

Mice were injected intraperitoneally once daily for 7 days with a dose of 1 mg/kg *i.p*. Scale bar, 50

μm.

File name: Supplementary Movie 2

Description: Sample movie montage of microglia and blood vessels with 7-day continuous saline injection, providing a control for Supplementary Movie 1. All other conventions are as in Supplementary Movie 1.

File name: Supplementary Movie 3

Description: Sample movie montage compiled from daily imaging (1 frame/day) of microglia (green) and blood vessels (red) showing the leakage of 10 kDa fluorescent dextran after LPS injection. The fluorescent dextran was injected intravenously before each imaging session. LPS was injected intraperitoneally once daily for 7 days at a dose of 1 mg/kg *i.p*. The same video images were used to simultaneously quantify BBB permeability and to determine microglia dynamics and morphology. Scale bar, 50 μm.

File name: Supplementary Movie 4

Description: Sample movie in which 10 kDa dextran was pseudocolored with gray to clearly visualize its leakage from blood vessels. The original images were from Supplementary Movie 3.
