# Supplementary material for: Dual microglia effects on blood brain barrier permeability induced by systemic inflammation
Source: Nat Commun. 2019 Dec 20;10:5816. doi: 10.1038/s41467-019-13812-z (PMC6925219; doi:10.1038/s41467-019-13812-z)
Supplement: Supplementary file 2 — Peer Review [file 41467_2019_13812_MOESM2_ESM.pdf]

Reviewers' comments:

Reviewer #1 (Remarks to the Author):

Dual microglia effects on blood brain barrier permeability induced by systemic inflammation
By Koichiro Haruwaka, Yoshihisa Tachibana, Ako Ikegami, Daisuke Kato, Riho Ono, Andrew J Moorhouse, Junichi Nabekura, Hiroaki Wake

Here the authors study the microglia contribution to blood-brain barrier (BBB) breakdown in the systemic models of inflammation. They simultaneously followed dynamic changes of microglia activation and localization and BBB leakage in longitudinal two-photon microscopy experiments.

First, in the MRL/lpr mouse model, they show increase in number of vessel-associated microglia compared with control animals, while the total number of microglia remained unchanged. They also confirmed that systemic inflammation leads to opening of BBB and leakage of 10 kDa dextran into the brain parenchyma. Next, they induced systemic inflammation with LPS in naive mice to study microglia activation and BBB breakdown simultaneously and they found that vessel-associated microglia accumulation precedes BBB breakdown. Interestingly, when they ablated microglia during LPS injections, they found increased BBB permeability during the early inflammatory phase. Besides the migration of microglia towards the vessels in systemic inflammation, they also studied morphological changes of microglia as they migrate and showed that the migrating microglia has shorter and fewer processes and is becoming activated. The two-photon in vivo imaging experiments are beautifully executed. Unfortunately, it is disappointing that the experimental attempt to determine some kind of mechanistic explanation, was lacking. This is just left to speculation in the Discussion on possible pathways instead, with no sense of whether one pathway may be more likely then another. Additional experiments to gain some mechanistic insight into microglial function in this context, and clarification of the Cldn5 data, along with other points detailed below would greatly strengthen the manuscript.

Major:
1. LPS is known to cause pericyte loss and thereby BBB leakage (Sci Rep. 2016 Feb 12;6:20931. doi: 10.1038/srep20931; Cell Mol Neurobiol. 2009 May;29(3):309-16. doi: 10.1007/s10571-008-9322-x). Pericytes are gate keepers of the BBB and are essential for BBB integrity (Neuron. 2010 Nov 4;68(3):409-27. doi: 10.1016/j.neuron.2010.09.043, Nat Med. 2019 Feb;25(2):270-276. doi: 10.1038/s41591-018-0297-y, J Cereb Blood Flow Metab. 2016 Jan;36(1):216-27, Neuron. 2015 Jan 21;85(2):296-302. doi: 10.1016/j.neuron.2014.12.032). Interestingly, pericytes are pluripotent cells (Curr Pharm Des. 2008;14(16):1581-93) that acquire multipotent activity under pathological conditions, such as stroke, and may thus be a novel source of microglia (J Neuroinflammation. 2016 Mar 7;13(1):57. doi: 10.1186/s12974-016-0523-9). The authors should investigate whether or not pericytes are altered in their model.

2. A major weakness of this manuscript is the lack of attempt to determine mechanism. We are left wondering how microglia are attracted to the vasculature, and how they cause BBB changes? The authors should at least study some of the potential players (IFN and others) involved in this opposing microglial response. Furthermore, minocycline is rather general and established. The authors could have tested other drugs to narrow down the potential mechanism as well. This would greatly strengthen the paper.

3. Authors should determine whether microglia can directly form junctions with ECs to seal the leaky BBB because authors speculate that microglial processes may directly interface with systemic circulation and that’s what initiates microglial phenotypic changes. EM is one option to push forward this hypothesis. This would strengthen the manuscript as well.

4. The authors state on page 15 that they analyzed the gene profile of astrocytes and microglia,

comparing WT and MLR/lpr mice, but no gene data seems to be presented. What were the results of the gene profile analyses? How was Cldn5 identified as potential gene of interest when Cldn5 is not expressed in microglia (see the next comment below)? The implication is that the gene data led to choosing Cldn5 and CD68 for further study. The data and/or literature behind this rationale is missing. On a technical level, how were microglia and astrocytes isolated for these experiments?
Cldn5 expression in microglia is per se nonexistent based on substantial literature, and confirmed recently with RNA seq data published from the Betsholtz lab database (Nature, 554, 475-480 2018 and http://betsholtzlab.org/VascularSingleCells/database.html) and Ben Barres lab database (https://web.stanford.edu/group/barres_lab/brain_rnaseq.html) and recently reported in PMID: 30188322. It is well known that Cldn5 is only expressed in endothelial cells (ECs). The authors' second hypothesis is that Cldn5 may be upregulated in ECs to strengthen tight junctions in response to a signal from microglia. The authors should try to design another set of experiments to eventually show (1) Cldn5 upregulation in ECs (FISH or FACS) after LPS and in their SLE model, and (2) What is the microglial "signal" that can trigger this Cldn5 upregulation in both models? Is IFN one candidate? Intraparenchymal injection of recombinant IFN would tell whether microglia can turn into a phagocytic phenotype and whether this leads to an increased expression of TJs and Cldn5 in ECs, for example.

5. The sepsis LPS model is quite severe (1 mg/kg ip daily for 7 days). Mice are probably close to being lethargic. What is the rational to use such a high dose and so many days? Systemic inflammation is sufficient with a single dose as it sustains over several days.

6. It is unclear what "leakage" is shown in the examples of dextran leakage. The color scheme used in figures such as 1d make it appear that mainly the vessels themselves are getting brighter. Furthermore, the methodology of how this was done should be more detailed. For instance, was any normalization to fluorescence intensity at time = 0/time of injection performed? Were only parenchymal areas evaluated, or intensity of vasculature included? It is difficult to evaluate this data without these details, but critical to the author's claims since this is the only means of evaluating leakage in the manuscript.

7. For the experiments in figure 4, it may be better to use a more traditional endothelial vessel marker such as CD31 or tomato lectin to compare localization with Cldn5. Using Aqp4-positive astrocyte endfeet to mark vasculature may not accurately represent the location of Cldn5. Furthermore, Cldn5 staining appears non-specific, making the colocalization evaluations unconvincing.

8. Data would be more representative if quantified by mouse, not field or cell number. Additional in vivo microglia data (increase n-numbers) would be beneficial as well, as the methods state as few as 3 microglia were evaluated per mouse.

9. What is the rationale for quantifying Pearson's coefficients (see e.g. figure 1b, 4b)? Why not evaluate the data directly?

Minor:

10. The supplemental movies should have all frames contrast adjusted to maintain consistent brightness across frames. In video 1, it is difficult to follow the movement of the microglia with so much variation in contrast. Arrows pointing out a couple notable examples would be helpful as well. Also, please slow the video down a bit. It is only 2 sec long presently, so it is over almost as soon as it starts.

11. It is not clear what video 2 is illustrating. The images are grayscale and the caption does not match the movie.

12. Authors should cite the original papers instead of reviews in the discussion section. For instance, reference 39.

13. It would be nice if the authors indicated somewhere that Aqp4 is just used as a vessel marker (except in figure 5). If it was intended to be more then that, this should be better emphasized in the text.

14. What is the co-localization correlation coefficient (top of the page 8)?

15. How was dextran administered?

16. Figure 1a would benefit from a magnified view to illustrate their data.

17. Figure 2d, 2e should include control animal examples and data for comparison.

18. What is the difference between two graphs below figure 2f containing data for vessel-associated microglia?

19. How many microglia cells were used for quantification for figure 3d and e?

20. Please include representative images for figure 4 panels a-d for WT mice.

21. CD68 (green) is not apparent in figure 4h examples.

22. What represents the purple color in figure 4l?

23. Need representative image for vehicle-treated group for figure 5c.

24. Table S2 may be easier to follow as a graph.

25. Please check phrasing for the usage of “leak.” In some places other variants may be more appropriate, e.g. leaky, leaked, leakage, etc.

26. Typo florescent? Page 8, row 1.

Reviewer #2 (Remarks to the Author):

The manuscript by Haruwaka and colleagues describes a biphasic response of CNS microglia to systemic inflammation: During the initial phase of systemic inflammation, CNS blood vessels appear to signal to parenchymal, tissue surveying microglia. These cells then become associated with the vasculature. By induction of tight junction molecules such as claudin-5, vessel-associated microglia appear beneficial in maintaining the integrity of the blood-brain barrier. During the second/chronic phase of inflammation, vessel-associated microglial cells switch to a more detrimental phenotype. By phagocytozing astroglial endfeet, they contribute to a partial opening of the BBB to molecules in the size range of about 10 kD.
This work addresses an interesting question in neuroscience: How sensitive is the immune-privileged brain to peripheral inflammatory insults. The authors observe microglial responses by in vivo multi-photon imaging in various genetic mouse models including the MRL/lpr mouse model of chronic inflammation, systemic LPS injections or selective diphtheria toxin-mediated microglia ablation.
The individual experiments are well performed and described. Furthermore, the authors provide a putative molecular mechanism.
Although the results appear in line with the conclusions of the authors, the light microscopic evidence for a molecular mechanism, i.e. claudin-5 expression and phagocytosis of AQP4-positive astroglial end

feet by microglia in figure 4, is not convincing. The authors provide some selected examples of orthogonal views to support their claims. However, immuno-EM is required to clearly demonstrate claudin-5 expression on microglia or on endothelial cells. Similarly, an ultrastructural analysis could more clearly demonstrate the phagocytosis of astroglial end feet as well as the junctions between microglia and endothelium (hypothesized on page 22) . Such experiments should be complemented by direct image recordings of the phagocytic event in vivo.

Minor points:
For a more complete description of data, the figure legends should contain information which figures/images were taken by confocal microscopy after immunostaining.
Figure 2, last sentences: replace 'point' by 'line'
Figure 3a: replace 'prenchymal' by 'parenchymal'. Left Y axis is 'Number of processes'.
Figure 4k: 'puncta' not 'pancta'

Reviewer #3 (Remarks to the Author):

This study by Haruwaka et al. focuses on an important topic, microglial interactions with the vasculature, in the context of systemic inflammation. Several limitations pertaining to the experimental design however reduce the impact of the findings, including sample size and statistical analyses. Suggestions to improve the manuscript are detailed below.

Major points

1. The work of Dimitrios Davalos on microglial interactions with the vasculature mediated through fibrinogen recognition should be cited and discussed.

2. The sample size "n" sometimes refers to fields, sometimes to microglial cells, sometimes to animals. It would be important to use n=animal, if not, microglia, across the analysis. Fields is not biologically relevant as a sample size.

3. The consequences of microglial ablation on astrocytes should be investigated considering that these glial cells are directly involved in the maintenance of the BBB.

4. In the Discussion, the authors mention "we speculate that microglia may directly form junctions with endothelial cells to seal the leaky BBB".
It would be important to determine experimentally whether this is the case. Super-resolution microscopy or electron microscopy could be performed to assess direct contacts between microglial processes and endothelial cells. Of note, the basal membrane should be positioned between the endothelial cells and microglia if the neurovascular unit is intact.

5. "By phagocytosing the astrocytic end-feet that ensheath the endothelial cells during more sustained systemic inflammation the integrity of the BBB would be compromised."
It would be important to also determine experimentally whether microglia can phagocytize or not astrocytic end-feets. Co-localization between microglia, astrocytes and lysosomal markers could be performed, as well as electron microscopy analyses.

6. The different size of Dextran able to leak inside the parenchyma was tested with the SLE model but not with the LPS model. Since these are two different models, the Dextran size should have been tested with both models.

7. For the minocycline experiment, a control (minocycline without LPS) should have been added to make sure the minocycline alone did not have an effect on the average microglial process length and

soma area.

8. Intranigral injections of LPS in rats were seen to cause expression of both the protein and mRNA of Aqp4 in “reactive” microglia (Tomas-Camardiel M, 2004). How do the authors know that the aqp4 puncta seen in vessel-associated microglia are phagocytosed astrocytic end-feets and not the microglia itself expressing aqp4 due to LPS? It would be important to validate this finding using other approaches (e.g. colocalisation with lysosomal marker, electron microscopy). Importantly, the spatial resolution of two-photon in vivo imaging does not allow to determine direct contacts between cells.

9. The authors used a MRL/lpr mice model to study the impact of chronic systemic inflammation on microglia in the motor cortex. These mice have been shown to exhibit early depression-like behavior and other behavioral alterations which could influence how microglia behave. The authors have not addressed the impact that the brain pathology has on microglia in the motor cortex and how the significant differences they see could be caused by something else than systemic inflammation.

10. For the morphology analysis, it is stated in the method sections that “at least three microglia were analyzed for each animal”. The range of microglial cells analyzed was not indicated in the methods. Moreover, a minimum of three microglial cells per animal is far too low to yield reliable and reproducible results. I would thus recommend to increase the number of microglial cells analyzed. The number of animals analyzed for the number of processes and length is also not the same as the soma in various conditions. Why did the authors use a different number of animals to analyze these parameters?

11. The infiltration of macrophages was not addressed in the MRL/lpr mouse model. The protein used to identify resident microglia is IBA1, which stains all myeloid cells, as acknowledged by the authors. I would either recommend to use TMEM119 (which is a microglia-specific antibody) for the experiments or determine the % of infiltrating cells found in this model and use caution with wording. In the CX3CR1-GFP model, the authors stated that they did not detect any systemic macrophages infiltrating the brain but have not provided the analysis or result for the analysis.

12. For the Sall-1 experiment, the authors have looked at double positive cells (sall-1+, IBA1+) to study resident microglial cells that originate from the yolk sac. They have stated that the density of the double positive cells does not change after 7 days of LPS “suggesting that macrophage do not contribute significantly to this vessel-associated microglia population”. This result means that the population of microglial cells does not change after the LPS injection in this model. It does not mean to my understanding that macrophages do not infiltrate. The authors should analyze the population of Sall-1-/IBA1+ cells (macrophage population) to make this claim. Moreover, they have used the colocalization of IBA1 and Aqp4 to determine the microglia-vessel contacts but did not mention if they only used only sall-1 positive cells in their analysis. If not, some of the cells could be macrophages since infiltration was not studied in this model.

13. The authors have used two different models to examine chronic (MRL/lpr) and sub-acute (LPS) systemic inflammation. The authors have not addressed if the inflammation in both models uses the same pathways.

Minor points

1. Although the manuscript was generally well written, several grammatical mistakes were identified across the figures and text. The manuscript should be revised professionally.

2. Throughout the manuscript, the terminology “activation” was used when referring to microglia. Please update the terminology as microglia adopt various phenotypes depending on the context.

3. The role of microglia in regulating cerebral blood flow should be discussed. For instance, see https://www.ncbi.nlm.nih.gov/pubmed/28559417.

4. Page 4: "Microglia do not directly ensheath endothelial cells".
Microglia were previously shown to make direct contacts with the vasculature with their processes sometimes ensheathing the basal lamina. This statement should be revised.

5. Page 7 : MRL should be defined.

6. I would recommend that the author evaluate microglial release of cytokine/ROS and MMP in both models (especially in the later stage of inflammation where the authors state that microglia become detrimental to the BBB permeability) to help strengthen their hypothesis.

7. The authors indicated that they have identified genes involved in various processes that were upregulated in the SLE mouse model but did not mention which genes nor did they show the data.

8. It was not indicated by the authors if the same animals were used for both the 2-photons and post-mortem experiments (IHC). How the effects of cranial windows on microglia and inflammation were assessed is also not mentioned.

9. The authors have stated in their methods that they have used both unpaired and paired t-tests for their experiments. However, I could not find experiments where a paired test was used. The authors have not stated in their methods if the mice used for each experiment were from the same litter.

10. The statistical software used for analysis should be mentioned.

Answer for the reviewer’s comments

> Reviewers' comments:
>
> Reviewer #1 (Remarks to the Author):
>
> Dual microglia effects on blood brain barrier permeability induced by systemic inflammation
> By Koichiro Haruwaka, Ako Ikegami, Yoshihisa Tachibana, Nobuhiko Ohno, Akari Hashimoto, Mami Matsumoto, Daisuke Kato, Riho Ono, Andrew J Moorhouse, Junichi Nabekura and Hiroaki Wake
>
> Here the authors study the microglia contribution to blood-brain barrier (BBB) breakdown in the systemic models of inflammation. They simultaneously followed dynamic changes of microglia activation and localization and BBB leakage in longitudinal two-photon microscopy experiments.
>
> First, in the MRL/lpr mouse model, they show increase in number of vessel-associated microglia compared with control animals, while the total number of microglia remained unchanged. They also confirmed that systemic inflammation leads to opening of BBB and leakage of 10 kDa dextran into the brain parenchyma. Next, they induced systemic inflammation with LPS in naive mice to study microglia activation and BBB breakdown simultaneously and they found that vessel-associated microglia accumulation precedes BBB breakdown. Interestingly, when they ablated microglia during LPS injections, they found increased BBB permeability during the early inflammatory phase. Besides the migration of microglia towards the vessels in systemic inflammation, they also studied morphological changes of microglia as they migrate and showed that the migrating microglia has shorter and fewer processes and is becoming activated. The two-photon in vivo imaging experiments are beautifully executed. Unfortunately,
> it is disappointing that the experimental attempt to determine some kind of mechanistic explanation, was lacking. This is just left to speculation in the Discussion on possible pathways instead, with no sense of whether one pathway may be more

likely then another. Additional experiments to gain some mechanistic insight into microglial function in this context, and clarification of the Cldn5 data, along with other points detailed below would greatly strengthen the manuscript.

>

> Major:

> 1. LPS is known to cause pericyte loss and thereby BBB leakage (Sci Rep. 2016 Feb 12;6:20931. doi: 10.1038/srep20931; Cell Mol Neurobiol. 2009 May;29(3):309-16. doi: 10.1007/s10571-008-9322-x). Pericytes are gate keepers of the BBB and are essential for BBB integrity (Neuron. 2010 Nov 4;68(3):409-27. doi: 10.1016/j.neuron.2010.09.043, Nat Med. 2019 Feb;25(2):270-276. doi: 10.1038/s41591-018-0297-y, J Cereb Blood Flow Metab. 2016 Jan;36(1):216-27, Neuron. 2015 Jan 21;85(2):296-302. doi: 10.1016/j.neuron.2014.12.032). Interestingly, pericytes are pluripotent cells (Curr Pharm Des. 2008;14(16):1581-93) that acquire multipotent activity under pathological conditions, such as stroke, and may thus be a novel source of microglia (J Neuroinflammation. 2016 Mar 7;13(1):57. doi: 10.1186/s12974-016-0523-9). The authors should investigate whether or not pericytes are altered in their model.

We thank the reviewer for raising this idea about pericytes as regulators of the BBB. In response to this point, we measured the number of pericytes in our two systemic inflammation models using immunohistochemistry for the pericyte marker, platelet-derived growth factor receptor beta (PDGFRβ). The number of pericytes in MRL/lpr mice was comparable to that in WT mice (Supplementary Fig. 1). Consistently, the number of pericytes in mice following both a single LPS injection, and after 7 days LPS injections is also comparable to the control mice without LPS (Supplementary Fig. 1). As indicated by the reviewer, previous studies have shown a decrease in pericyte number, or disrupted pericyte-vessel interaction, in response to LPS. The reason why our own studies seem to contradict this idea is unknown. One of the previous studies (Zeng et al., 2016) used 2mg/kg LPS i.p. but examined pericytes in peripheral tissues, while the other study (Nishioku et al., 2009) used a high dose of LPS (20mg/kg) and showed a concomitant activation of microglia and disrupted vascular permeability (i.e., consistent with our data). In other studies of brain disorders or traumas where reduced pericyte coverage of vessels is observed (eg, hypoperfusion injury, Liu et al., 2019), the increase in BBB permeability allows much larger molecules (70kDa or more) and may

be a different type of BBB disruption than we have reported (where only substances < 40kDa only may leak). In our study we saw no loss of pericytes or obvious disruption of pericytes relative to the endothelial cells, such as pericytes peeling or migrating away from vessels (Dore-Duffy et al., 2000). Hence, we appreciate this point of possible pericyte involvement, but feel the data does not provide any insights into how pericytes may, if at all, contribute to changes in vascular permeability or microglial numbers in our model. We have included the new data in Supplementary Fig. 1, and raised these points about pericytes in the main text (results and discussion).

>

> 2. A major weakness of this manuscript is the lack of attempt to determine mechanism. We are left wondering how microgla are attracted to the vasculature, and how they cause BBB changes? The authors should at least study some of the potential players (IFN and others) involved in this opposing microglial response. Furthermore, minocycline is rather general and established. The authors could have tested other drugs to narrow down the potential mechanism as well. This would greatly strengthen the paper.

We acknowledge that our original manuscript was not able to provide a complete mechanistic link for what causes microglia migration to vessels and for what factors may contribute to changes in vasculature permeability. We have now conducted additional experiments addressing these queries raised. To examine potential mediators causing microglial attraction, we used initially cultured endothelial cells, and incubated cells with either LPS or IFNα. We used IFNα is this was the most abundant cytokine detected in human blood sample in SLE patients and in human CSF of SLE psychosis patients. In these cultured endothelial cells, we detected elevation of CCL5 in the media after treatment with LPS and IFNα (Fig. 6a, b). This leads us to examine if CCL5 release *in vivo* may be attracting microglia and/or vessel interactions via activation of its CCR5 receptor. We did this via intra-ventricular administrated of the CCR5 antagonist DAPTA (0.4 mg/mice), injected 3 days before intraperitoneal injection of LPS or IFNα (for 4 days) with *in vivo* imaging of microglia as before. In addition, we isolated microglia using magnetic activated cell sorting (MACS) and subsequent RNA collection, to quantify Cldn5 expression. In mice treated with DAPTA, there was a significant reduction of both microglia migration to vessels (Fig. 6c-h), and a significant decrease in the extent of Cldn5 expression in these microglia (Fig. 6i). Consistently, the

BBB permeability in DAPTA treated mice increased on day 2 of LPS injection (Fig. 6f). In addition, intra-ventricular injection of IFNα promote the CD68 expression in microglia (Fig. 6j). Hence, our new data provide greater strength and clarity for our originally ideas about Cldn5, and reveal mechanisms about how microglia are attracted to vessels. In summary, our data shows that endothelial cells activated by systemic inflammation promote CCL5 release to attract microglia and to promote Cldn5 expression in microglia that can make tight junctions between microglia and endothelial cells to protect BBB permeability. As indicated below, we also have new electron microscopy evidence supporting this mechanism.

> 3. Authors should determine whether microglia can directly form junctions with ECs to seal the leaky BBB because authors speculate that microglial processes may directly interface with systemic circulation and that's what initiates microglial phenotypic changes. EM is one option to push forward this hypothesis. This would strengthen the manuscript as well.

We again acknowledge the reviewer's comment that direct high resolution images could help distinguish if microglia directly interact with other elements of the neurovascular unit. We have now conducted, as suggested, immuno-electron microscopy (immuno-EM), using the MRL/lpr model mice. Our new data does indeed directly show that microglial processes surround parts of the basal membrane and can infiltrate through the basal membrane and directly contact the endothelial cells. We saw discrete spots of Cldn5 immunoreactivity at some of these sites (Fig. 4e). This supports the conclusion that microglia form functional tight junctions with endothelial cells to help preserve BBB integrity.

> 4. The authors state on page 15 that they analyzed the gene profile of astrocytes and microglia, comparing WT and MLR/lpr mice, but no gene data seems to be presented. What were the results of the gene profile analyses? How was Cldn5 identified as potential gene of interest when Cldn5 is not expressed in microglia (see the next comment below)? The implication is that the gene data led to choosing Cldn5 and

CD68 for further study. The data and/or literature behind this rationale is missing. On a technical level, how were microglia and astrocytes isolated for these experiments?

> Cldn5 expression in microglia is per se nonexistent based on substantial literature, and confirmed recently with RNA seq data published from the Betsholtz lab database (Nature, 554, 475-480 2018 and http://betsholtzlab.org/VascularSingleCells/database.html <http://betsholtzlab.org/VascularSingleCells/database.html>) and Ben Barres lab database (https://web.stanford.edu/group/barres_lab/brain_rnaseq.html <https://web.stanford.edu/group/barres_lab/brain_rnaseq.html>) and recently reported in PMID: 30188322. It is well known that Cldn5 is only expressed in endothelial cells (ECs). The authors' second hypothesis is that Cldn5 may be upregulated in ECs to strengthen tight junctions in response to a signal from microglia. The authors should try to design another set of experiments to eventually show (1) Cldn5 upregulation in ECs (FISH or FACS) after LPS and in their SLE model, and (2) What is the microglial "signal" that can trigger this Cldn5 upregulation in both models? Is IFN one candidate? Intraparenchymal injection of recombinant IFN would tell whether microglia can turn into a phagocytic

> phenotype and whether this leads to an increased expression of TJs and Cldn5 in ECs, for example.

We appreciate the reviewer detailed and thoughtful comments, and suggestions. As suggested, we now show the microarray data as Supplementary Fig. 4. For this experiment, microglia and astrocytes were isolated from whole brain using magnetic activated cell sorting (MACS), using beads coated with CD11b and GLAST antibodies. The methodology has been now added to the text.

We appreciate this is not as expected, with Cldn5 typically associated with EC, and hence our secondary hypothesis. However, as also suggested by the reviewer, we have now specifically examined Cldn5 expression in EC cells using FACS sorting from dissected brains (Supplementary Fig. 6a). We couldn't detect any differences in the Cldn5 positive EC population after LPS injection, nor in MRL/lpr mice (Supplementary Fig. 6c). However, Cldn5 positive microglia population increased after LPS injection, and in MRL/lpr mice (Supplementary Fig. 6b). We assume that Cldn5 expression is switched on by a specific inflammation induced factor, and not detectable under basal or other conditions in microglia. Indeed, as described above, we have now identified

CCR5 signaling as (at least partly) that switches on Cldn5 expression in microglia (Fig. 6i). Finally, we also further examined what message may upregulate the phagocytic microglial marker CD68. Intraventricular injection of IFNα significantly increased CD68 expression in microglial cells, indicating this is an important part of the signal converting microglia to the phagocytic phenotype (Fig. 6j).

> 5. The sepsis LPS model is quite severe (1 mg/kg i.p. daily for 7 days). Mice are probably close to being lethargic. What is the rational to use such a high dose and so many days? Systemic inflammation is sufficient with a single dose as it sustains over several days.

We agree that both acute and chronic LPS can induce sepsis, lethargy and indeed toxicity if doses are high enough. Our protocol is, however, within the mid range of that previously used to induce sustained systemic inflammation (Hoogland et al., 2015) and was only very occasionally toxic, although some modest lethargy was seen towards the end of the week. Our rationale for this protocol was to produce a sustained and slowly escalating inflammation. A single dose protocol doesn’t consistently induce inflammation that lasts a week (Hoogland et al., 2015), although it may sustain for a few days as indicated by the reviewer. Nevertheless, we have now examined the effects of a single LPS dose on microglia migration and BBB permeability. This single LPS dose (1 mg/kg, *i.p.*) did induce microglia migration (Supplementary Fig. 2a), but did not increase BBB permeability (Supplementary Fig. 2b). Consistent with the data from the weekly protocol, we suggest that the expression of Cldn5 increases in microglia during the early stages of inflammation. The new single dose data is reported in Supplementary Fig. 2.

> 6. It is unclear what “leakage” is shown in the examples of dextran leakage. The color scheme used in figures such as 1d make it appear that mainly the vessels themselves are getting brighter. Furthermore, the methodology of how this was done should be more detailed. For instance, was any normalization to fluorescence intensity at time = 0/time of injection performed? Were only parenchymal areas evaluated, or intensity of vasculature included? It is difficult to evaluate this data without these details, but critical to the author’s claims since this is the only means of evaluating leakage in the manuscript.

We acknowledge that the original Figure 1 may not have been as clear as we had intended, and appreciate the reviewer pointing out some specific areas to clarify. We have now changed the representative images in Fig. 1d, 2d, 2h, also using a different color scheme to help make the BBB dextran leakage clearer. Using fluorescent dextran beads we aim to show differences in dextran outside the vessels to represent “leakage”. We have now provided more detailed methodology in the revised text including the specific queries raised. To specifically respond to the reviewer’s queries: i) yes, the fluorescence intensity was normalized to that of the intensity in first day; and ii) the quantified fluorescence was that in the parenchyma only – the vessels were traced in Image J and these vessel regions excluded from the subsequent image analysis. In addition, we used same laser power and the same fluorescence detection settings for each mouse across the imaging sessions.

> 7. For the experiments in figure 4, it may be better to use a more traditional endothelial vessel marker such as CD31 or tomato lectin to compare localization with Cldn5. Using Aqp4-positive astrocyte end feet to mark vasculature may not accurately represent the location of Cldn5. Furthermore, Cldn5 staining appears non-specific, making the colocalization evaluations unconvincing.

As indicated above, we have conducted immuno-EM experiments to further confirm the increase in Cldn5 in microglia (Fig. 4e). We have also used FACS cell sorting to confirm Cldn5 in microglial cells (Supplementary Fig. 6). As suggested, we have also conducted additional experiments using CD31 immunohistochemistry as the marker for endothelial cells, with similar results as seen with Aqp4. This CD31 data is shown in Supplementary Fig. 5.

> 8. Data would be more representative if quantified by mouse, not field or cell number. Additional in vivo microglia data (increase n-numbers) would be beneficial as well, as the methods state as few as 3 microglia were evaluated per mouse.

As suggested by the reviewer, and also as suggested by reviewer 3, we now indicated both microglia and mice for all data, and used number of mice in statistical analysis. We have also added more data to our *in vivo* microglia experiments.

> 9.	What is the rationale for quantifying Pearson's coefficients (see e.g. Fig. 1b, 4b)? Why not evaluate the data directly?

We appreciate for the reviewer' comments. We did also count the number of the microglia associated with vessels, expressing this as a proportion of the total microglia. Pearson's co-efficients are routinely used in immunofluorescent analysis as it gives an objective quantification of the extent of overlap of fluorescence at a pixel by pixel basis for two different fluorophores imaged from two different channels.

> Minor:
>
> 10.	The supplemental movies should have all frames contrast adjusted to maintain consistent brightness across frames. In video 1, it is difficult to follow the movement of the microglia with so much variation in contrast. Arrows pointing out a couple notable examples would be helpful as well. Also, please slow the video down a bit. It is only 2 sec long presently, so it is over almost as soon as it starts.

We appreciate this feedback, and have now adjusted the contrast to be consistent throughout, slowed the movie down, and also marked some examples of microglia on the end of the movie in Supplementary Video 1.

> 11.	It is not clear what video 2 is illustrating. The images are grayscale and the caption does not match the movie.

Supplementary Video 2 was illustrating permeability of the BBB to the 10kDa dextran fluorophore. We have improved the quality of the Supplementary Videos by adjusting the contrast, and have corrected the caption.

> 12.	Authors should cite the original papers instead of reviews in the discussion section. For instance, reference 39.

We have read and added some original papers showing BBB leak and minocycline induced decreases in leak in hypoxia, ischemia and AD models.

> 13. It would be nice if the authors indicated somewhere that Aqp4 is just used as a vessel marker (except in figure 5). If it was intended to be more then that, this should be better emphasized in the text.

Aqp4 was only intended to be used as a vessel marker (except in Fig. 5 as indicated for the astrocyte end feet). We have emphasized this in main text.

> 14. What is the co-localization correlation coefficient (top of the page 8)?

The co-localization co-efficient is a measure of the merged pixel within the image. We quantified this by Pearson's coefficients. We have clarified this in both top of page 8, and in the Figure 1 legend where we 1st refer to the correlation co-efficient.

> 15. How was dextran administered?

Dextran was injected into the tail vein as a bolus infusion (2 mg/ml, 50μl) just prior to the image acquisition. Once the mouse was secured into the headframe and the microglia and vessels could be imaged, we would inject the Dextran. This additional detail has now been added to the methods section.

> 16. Figure 1a would benefit from a magnified view to illustrate their data.

Thanks for the suggestion, we have added a higher magnification panel to our images in Fig. 1a.

> 17. Figure 2d, 2e should include control animal examples and data for comparison.

Thanks, we have now added the data from control animal experiments for comparison.

> 18. What is the difference between two graphs below figure 2f containing data for vessel-associated microglia?

Thanks for pointing out this redundancy. Both Figures showed dominant reduction of vessel associated microglia in Iba1-DTA mice. As they both basically indicated the same result, we have deleted the one of the graph in the revised manuscript.

> 19. How many microglia cells were used for quantification for figure 3d and e?

In Fig. 3d, 3e that are currently in Fig. 3c, we analyzed 5 microglia in each of five mice, giving a total of 25 microglia. For the data in Fig. 3a, we analyzed 5 microglia in each of six mice, giving a total of 30 microglia. This is now indicated in the legend.

> 20. Please include representative images for figure 4 panels a-d for WT mice.

We have now included representative images for each of the panels shown in a-d for WT mice, as suggested.

>

> 21. CD68 (green) is not apparent in Fig. 4h examples.

Thanks for the feedback, we have changed to a color intensity to make CD 68 fluorescence more apparent or use different data example where the CD68 is more apparent.

> 22. What represents the purple color in figure 4l?

The purple line represents the trajectory of migrating microglial processes. We have added a description in the figure legend. Now it is in Fig. 5i

> 23. Need representative image for vehicle-treated group for figure 5c.

We have added a representative image for this group as suggested.

>

> 24. Table S2 may be easier to follow as a graph.

As Table S2 is a summary of the graph shown as Fig. 3a, we prefer to leave it as a Table so as to complement but not replicate Fig. 3a.

>

> 25. Please check phrasing for the usage of "leak." In some places other variants may be more appropriate, e.g. leaky, leaked, leakage, etc.

We have been through the manuscript as part of our thorough review and believe all tenses of "leak" are appropriate. We have now largely used "leak"

>

> 26. Typo florescent? Page 8, row 1.

Thanks, now corrected.

>

>

>

> Reviewer #2 (Remarks to the Author):

>

> The manuscript by Haruwaka and colleagues describes a biphasic response of CNS microglia to systemic inflammation: During the initial phase of systemic inflammation, CNS blood vessels appear to signal to parenchymal, tissue surveying microglia. These cells then become associated with the vasculature. By induction of tight junction molecules such as claudin-5, vessel-associated microglia appear beneficial in maintaining the integrity of the blood-brain barrier. During the second/chronic phase of inflammation, vessel-associated microglial cells switch to a more detrimental phenotype. By phagocytozing astroglial endfeet, they contribute to a partial opening of the BBB to molecules in the size range of about 10 kD.

> This work addresses an interesting question in neuroscience: How sensitive is the immune-privileged brain to peripheral inflammatory insults. The authors observe microglial responses by in vivo multi-photon imaging in various genetic mouse models including the MRL/lpr mouse model of chronic inflammation, systemic LPS injections or selective diphtheria toxin-mediated microglia ablation.

> The individual experiments are well performed and described. Furthermore, the authors provide a putative molecular mechanism.

1. Although the results appear in line with the conclusions of the authors, the light microscopic evidence for a molecular mechanism, i.e. claudin-5 expression and phagocytosis of AQP4-positive astroglial end feet by microglia in figure 4, is not convincing. The authors provide some selected examples of orthogonal views to support their claims. However, immuno-EM is required to clearly demonstrate claudin-5 expression on microglia or on endothelial cells. Similarly, an ultrastructural analysis could more clearly demonstrate the phagocytosis of astroglial end feet as well as the junctions between microglia and endothelium (hypothesized on page 22) . Such experiments should be complemented by direct image recordings of the phagocytic event in vivo.

We appreciate the positive comments on our manuscript and findings, and the constructive critical suggestions for providing stronger evidence for our conclusions. As also suggested by reviewer#1, we have now performed immuno-EM in the MRL/lpr mice. Our data shows that microglial processes invade in the parts of the basal membrane and directly contact the endothelial cells at discrete spots where the Cldn5 expressed (Fig. 4e). Coupled with the upregulation of Cldn5 in microglia, this supports the proposal that they form tight junctions with endothelial cells to help preserve BBB integrity.

It is very difficult for us to directly observe the phagocytic event and to identify the underlying components (e.g., the astrocytic end feet) using *in vivo* imaging. We don't have the spatial resolution *in vivo*. However, we used our immune-EM approach in MRL/lpr mice to address what has been phagocytosed. Microglia were closely associated with vessels at the ultrastructural level, and there were examples of where Aqp4 immunoreactivity was included within a microglial phagosome (Fig. 5h), supporting the idea that microglia phagocytose the astrocyte end feet.

> Minor points:

2. For a more complete description of data, the figure legends should contain information which figures/images were taken by confocal microscopy after immunostaining.

We have added this specific information to the legend where appropriate.

3. Figure 2, last sentences: replace 'point' by 'line'

Thanks, we have corrected this typo error.

4. Figure 3a: replace 'prenchymal' by 'parenchymal'. Left Y axis is 'Number of processes'.

Thanks, we have now fixed these.

5. Figure 4k: 'puncta' not 'pancta'

Thanks, we have corrected this typo error.

> Reviewer #3 (Remarks to the Author):
>
> This study by Haruwaka et al. focuses on an important topic, microglial interactions with the vasculature, in the context of systemic inflammation. Several limitations pertaining to the experimental design however reduce the impact of the findings, including sample size and statistical analyses. Suggestions to improve the manuscript are detailed below.
>
> Major points
>
> 1. The work of Dimitrios Davalos on microglial interactions with the vasculature mediated through fibrinogen recognition should be cited and discussed.

We appreciate the advice and agree that this work indicating a role for fibrinogen released from leaky vessels to attract microglia and elicit phenotypic changes shows that other signaling mechanisms may exist in different conditions. We have acknowledged this in the revised discussion, referring to the work of Davalos.

> 2. The sample size "n" sometimes refers to fields, sometimes to microglial cells, sometimes to animals. It would be important to use n=animal, if not, microglia, across the analysis. Fields is not biologically relevant as a sample size.

As also raised by reviewer#1. We have now indicated both microglia and mice for all data, and used number of mice in statistical analysis. We have also added more data to our *in vivo* microglia experiments.

> 3. The consequences of microglial ablation on astrocytes should be investigated considering that these glial cells are directly involved in the maintenance of the BBB.

We appreciate the role of astrocytes in BBB integrity, and the interactions they play with microglia. As an initial but relevant investigation, we have investigated how partial microglia ablation effects microglia and astrocyte numbers, concurrently with blood brain integrity (Supplementary Fig. 3). Using our conditional microglia knock-out

mouse, removal of doxycycline reduced microglia cell counts by 20-30%, and simultaneously increased GFAP mean intensity by 20-30%. This indicates a modest astrocyte activation or proliferation upon microglia depletion, however BBB integrity was unaffected. The data is now included in Supplementary Fig. 3.

> 4. In the Discussion, the authors mention "we speculate that upon microglia may directly form junctions with endothelial cells to seal the leaky BBB".
> It would be important to determine experimentally whether this is the case. Super-resolution microscopy or electron microscopy could be performed to assess direct contacts between microglial processes and endothelial cells. Of note, the basal membrane should be positioned between the endothelial cells and microglia if the neurovascular unit is intact.

We agree this was an important speculation to provide further evidence for or against, and have now done these experiments, as also indicated above in response to similar comments from reviewers 1 and 2. We performed immuno-EM in MRL/lpr mice and showed that the microglial processes surround the basal membrane invade into and through the basal membrane to make direct contacts with endothelial cells, and Cldn5 immunoreactivity can be seen at these discrete locations. Yes, the basal membrane was between microglia and endothelial cells, but there were examples where microglia were to the sides of the basal membrane, or surrounding the basal membrane. The new data is shown in Fig. 4e.

> 5. "By phagocytosing the astrocytic end-feet that ensheath the endothelial cells during more sustained systemic inflammation the integrity of the BBB would be compromised."
> It would be important to also determine experimentally whether microglia can phagocytize or not astrocytic end-feets. Co-localization between microglia, astrocytes and lysosomal markers could be performed, as well as electron microscopy analyses.

We appreciate the suggestions for how to strengthen this conclusion, and have now conducted immuno-EM experiments on the MRL/lpr mice. Our data showed that microglial processes could be directly adjacent to astrocytes around vessels, and also found Aqp4 inclusions within microglial phagosomes. Our new data is shown in Fig. 5h,

and provides more direct evidence to support the idea that microglia phagocytose the astrocyte end feet.

> 6. The different size of Dextran able to leak inside the parenchyma was tested with the SLE model but not with the LPS model. Since these are two different models, the Dextran size should have been tested with both models.

We agree this is a fair comment, although we had examined LPS effects on vessel permeability with 10 kDa Dextra. We have now tested the three different sized Dextran beads in the LPS model, and the new data shown in Fig. 2f. As was seen for the MRL mice, the increase BBB permeability was only seen for the 10kDa dextran, 40kDa and 70 kDa remained restricted to the vessels.

> 7. For the minocycline experiment, a control (minocycline without LPS) should have been added to make sure the minocycline alone did not have an effect on the average microglial process length and soma area.

As suggested, we have now conducted this control experiment, examining the effects of minocycline alone on microglia parameters. The new data is shown in Supplementary Fig. 7. Minocycline by itself did not effect the averaged length of microglial processes, nor their soma area. Microglia migration to vessels or changes in BBB permeability were not stimulated by minocycline alone (Supplementary Fig. 7).

> 8. Intranigral injections of LPS in rats were seen to cause expression of both the protein and mRNA of Aqp4 in “reactive” microglia (Tomas-Camardiel M, 2004). How do the authors know that the aqp4 puncta seen in vessel-associated microglia are phagocytosed astrocytic end-feets and not the microglia itself expressing aqp4 due to LPS? It would be important to validate this finding using other approaches (e.g. colocalisation with lysosomal marker, electron microscopy). Importantly, the spatial resolution of two-photon in vivo imaging does not allow to determine direct contacts between cells.

We appreciate the reviewer raising this interesting possibility. As mentioned above (e.g., comment 5), we performed additional immuno-EM experiments using MRL/lpr mice and directly observed astrocyte reactivity in microglial phagosomes, and observed microglial processes in contact with astrocytes (Fig. 5h).

49. ミクログリアが Aqp4 を発現することもあるので、共局在だけでは貪食を証明できない

→追加実験なし:CD68-Aqp4 共局在率を検証し、internalization を明らかにする。（これまでの画像から microglia に ROI をとり、CD68 と Aqp4 の共局在率を出す。N=3）

> 9. The authors used a MRL/lpr mice model to study the impact of chronic systemic inflammation on microglia in the motor cortex. These mice have been shown to exhibit early depression-like behavior and other behavioral alterations which could influence how microglia behave. The authors have not addressed the impact that the brain pathology has on microglia in the motor cortex and how the significant differences they see could be caused by something else than systemic inflammation.

The reviewer raises an interesting possibility, and we cant rule out definitively other mechanisms beyond systemic inflammation. However, the consistency between the sequence of events in response to LPS and in the MRL, and the consistency of experimental manipulations, strongly support a high degree of commonality. Systemic inflammation initiating microglial responses and then changes in BBB permeability as we have concluded seems to us the most parsimonious explanation.

We do, however, recognize the cognitive abnormalities in MRL mice and have actually undertaken experiments to quantify motor learning. We trained MRL/lpr and control mice in a motor learning task in which mice must pull a lever and hold it down for over 600ms to get a water. Mice performed this task for one hour per day for 13 days, quantifying the proportion of trials in which the water reward was achieved. There was no significant difference in the success rate at day 1, but the success rate at days 10-13 (late phase) was lower in MRl/lpr mice as compared with those of control mice. This indicates some deficit in motor learning, which we would speculate is a consequence of impaired BBB permeability and microglia reactivity, as opposed to a cause of this. The data has been included in Supplementary Fig. 8.

> 10. For the morphology analysis, it is stated in the method sections that “at least three microglia were analyzed for each animal”. The range of microglial cells analyzed was not indicated in the methods. Moreover, a minimum of three microglial cells per animal is far too low to yield reliable and reproducible results. I would thus recommend to increase the number of microglial cells analyzed. The number of animals analyzed for the number of processes and length is also not the same as the soma in various conditions. Why did the authors use a different number of animals to analyze these parameters?

We have increased the number of samples. Specifically, we analyzed 5 microglia per mouse for each of 6 mice, so that a total of 30 microglia were measured in each condition.

Initially, we were not always able to measure processes and soma for each microglia (in the same planes), hence different numbers were initially reported. However, by re-examining the images we were able to more carefully identify suitable (additional) microglia to analyze and thereby keep consistent the number of microglia analyzed for the cell soma and process.

> 11. The infiltration of macrophages was not addressed in the MRL/lpr mouse model. The protein used to identify resident microglia is IBA1, which stains all myeloid cells, as acknowledged by the authors. I would either recommend to use TMEM119 (which is a microglia-specific antibody) for the experiments or determine the % of infiltrating cells found in this model and use caution with wording. In the CX3CR1-GFP model, the authors stated that they did not detect any systemic macrophages infiltrating the brain but have not provided the analysis or result for the analysis.

We appreciate this comment about possible macrophage or other myeloid cell infiltration, and the use of TMEM119. We did do the immunostaining for TMEM119 in MRL/lpr mice and found, at least in the visible fields, 100% of IBA1+’ve identified microglia also expressed TMEM119, even for microglia associate with vessels. Since

we saw 100% of microglia expressing TMEM119, we have stated but not showed the quantification. We added a representative image as Fig. 3d.

> 12. For the Sall-1 experiment, the authors have looked at double positive cells (sall-1+, IBA1+) to study resident microglial cells that originate from the yolk sac. They have stated that the density of the double positive cells does not change after 7 days of LPS “suggesting that macrophage do not contribute significantly to this vessel-associated microglia population”. This result means that the population of microglial cells does not change after the LPS injection in this model. It does not mean to my understanding that macrophages do not infiltrate. The authors should analyze the population of Sall-1-/IBA1+ cells (macrophage population) to make this claim. Moreover, they have used the colocalization of IBA1 and Aqp4 to determine the microglia-vessel contacts but did not mention if they only used only sall-1 positive cells in their analysis. If not, some of the cells could be macrophages since infiltration was not studied in this model.

We acknowledge the point being made and have therefore now also quantified the number of Sall-1-/IBA1+ cells. which was very close to zero in saline control mice. This number did not significantly increase in LPS mice. This data has now been added as a new column in Fig. 3e.

> 13. The authors have used two different models to examine chronic (MRL/lpr) and sub-acute (LPS) systemic inflammation. The authors have not addressed if the inflammation in both models uses the same pathways.

In the revised manuscript we have increased the number of experimental manipulations common to both the LPS and MLR models. In both models microglia are associated with vessels and a concurrent increase in BBB permeability, and both models are associated with similar microglia morphologies (reduced branches and soma) and the same permeability cut-off (10 kDa Dextran). In terms of signaling pathways, both models are associated with increases in Cldn5 and/or CD68 in microglia - for LPS the degree differing relative to the cumulative total LPS injections, for MLR mice the difference relative to the extent of putative reactivity. We have now performed

additional experiments to elucidate the signaling molecules for the LPS model, and this also shows similarities to MLR.

In the new experiments, we initially cultured endothelial cells and treated them with both LPS and IFNα. We used IFNα is this was the most abundant cytokine detected in human blood sample in SLE patients and in human CSF of SLE psychosis patients, and thereby we partially mimicked the condition of MLR combing relevant cytokines and inflammation. We performed a cytokine array and detected CCL5 elevation in the culture medium in cells treated with LPS and IFNα (Fig. 6a, b). We then administrated the CCR5 antagonist (DAPTA 0.4mg/mice) via intraventricular injection, 3 days before the start of a 4 day daily LPS i.p. injection or intravenous IFNα injection combined with *in vivo* imaging and subsequent microglia isolation and RNA analysis for Cldn5 expression (Fig. 6). Mice treated with DAPTA had significantly less microglia migration towards vessels in response to LPS or IFNα injection (Fig. 6c-h), and significantly reduced Cldn5 expression 4 days after LPS injection (Fig. 6i). Hence the IFNα appears to be part of the triggering signaling pathway for the sequence of events that we propose are common to MLR and LPS. However, we recognize we have only shown IFNα/CCR5 as part of the LPS response, and mention this in the discussion.

>

> Minor points

>

> 14. Although the manuscript was generally well written, several grammatical mistakes were identified across the figures and text. The manuscript should be revised professionally.

We have thoroughly checked the manuscript, assisted by our English-speaking co-authors.

> 15. Throughout the manuscript, the terminology “activation” was used when referring to microglia. Please update the terminology as microglia adopt various phenotypes depending on the context.

We have replaced the broad use of “activated” throughout the manuscript, being more specific with the microglia phenotype where possible,.

>16. The role of microglia in regulating cerebral blood flow should be discussed. For instance, see https://www.ncbi.nlm.nih.gov/pubmed/28559417.
<https://www.ncbi.nlm.nih.gov/pubmed/28559417.>

Thank you. We have consulted this paper. Although we found this very timely and interesting, we could not see how the conclusions could be directly linked to our results without opening up a new area of discussion slightly outside our direct results.

17. Page 4: "Microglia do not directly ensheath endothelial cells".

> Microglia were previously shown to make direct contacts with the vasculature with their processes sometimes ensheathing the basal lamina. This statement should be revised.

Thanks, We revised this statement. Our new EM data (Figs. 4 and 5) also shows that microglial processes can surround or ensheath the basal membrane, by extension suggesting they may also ensheath the EC, so we removed this statement.

18. Page 7 : MRL should be defined.

Thanks. We have defined MRL on 1st use (MRL = Murphy Roths Large).

19. I would recommend that the author evaluate microglial release of cytokine/ROS and MMP in both models (especially in the later stage of inflammation where the authors state that microglia become detrimental to the BBB permeability) to help strengthen their hypothesis.

We agree with the reviewer to fully examine the signaling molecules mediating the increased BBB permeability with more sustained or sever inflammation. Our data suggests upregulation of CD68 and microglia induced phagocytosis of astrocytic end feet as part of the mechanism. We further agree that microglial release of MMPs, ROS and cytokines as likely candidates. We did undertake additional experiments as described above, and focused on the more novel initial stages – the signal that attracts microglia to vessels and how microglia may preserve BBB integrity. As mentioned above, we used cultured endothelial cells treated with LPS and IFNα to identify CCL5 release and then used a CCR5 antagonist to implicate this signaling pathway in microglia migration and Cldn5 induced preservation of BBB integrity in earlier stages

of LPS induced inflammation. Similarly elucidating the signaling pathways in the loss of BBB integrity is interesting, but would not be possible in the timeframe of this paper.

20. The authors indicated that they have identified genes involved in various processes that were upregulated in the SLE mouse model but did not mention which genes nor did they show the data.

We acknowledge this lack of detail, as also commented by other reviewers. We now include this microarray data as Supplementary Fig. 4.

21. It was not indicated by the authors if the same animals were used for both the 2-photons and post-mortem experiments (IHC). How the effects of cranial windows on microglia and inflammation were assessed is also not mentioned.

We used different mice for the *in vivo* imaging data and for the IHC data, and have now indicated this in the methods.

In regards to the effects of the cranial window on the microglia phenotype and subsequent responses, we agree this is an important consideration. We have experience in this in our previous papers where, for example, we used a thinned skull imaging to avoid any microglial reactivity due to the cranial window (Wake et al., 2009). In the present experiments we ensured a careful cranial window surgery and waited three weeks after surgery to ensure sufficient recovery and avoid any basal inflammation due to surgery. Our preliminary data evaluated that this recovery period was sufficient to avoid surgery induced inflammatory responses, and this is supported by the lack of any BBB leakage in control mice. We have now indicated this important point in the methods.

22. The authors have stated in their methods that they have used both unpaired and paired t-tests for their experiments. However, I could not find experiments where a paired test was used. The authors have not stated in their methods if the mice used for each experiment were from the same litter.

We thank the reviewer for raising this point. We did not use the paired t-test in any of our comparisons and have now deleted this. Where possible, we used mice from the

same litter, although this was not specifically matched to have even controls and tests from the same litter so we have not specified this.

> 10. The statistical software used for analysis should be mentioned.

We used Prism8 (GraphPad), and have now specified this in the methods.

Reviewers' comments:

Reviewer #1 (Remarks to the Author):

Although the authors addressed most of the concerns (17 out of 26), several points remain to be clarified and/or changed:

Point 1. The authors have now looked at the number of pericytes and did not find any changes after LPS challenge. The authors should consider looking at pericyte coverage (see Nikolakopoulou et al., Plos One 2017 for details) which could still be affected. The authors should clarify which brain region is shown in Supp Fig 1. Looking at several region-of-interests such as cortex, hippocampus, and thalamus would be important. Also, the numbers of pericytes should be expressed as the number of PDGFRb+ DAPI+ double positive cells. For coverage, CD13 is probably more reliable marker (see Armulik et al., Nature 2010; Bell et al., Nature 2012).

Point 3: The authors suggest that their EM data show that Cldn5 from microglia contact BM. However, the origin of claudin 5 in their study is unclear, and it is well established that microglia have negligible expression of Cldn5 and that Cldn5 mRNA is only expressed in brain endothelial cells but not in microglia. See for example plots of relative expression on Cldn5 mRNA transcript from two landmark studies frequently cited by many people working in the field – i.e., Betsholtz (Vanlandewijck et al., Nature 2018, 554, 475-480; http://betsholtzlab.org/VascularSingleCells/database.html) and Barres (Zhang et al., J Neurosci 2014, 34.36, 11929-11947; https://web.stanford.edu/group/barres_lab/cgi-bin/igv_cgi_2.py?lname=Cldn5) databases.

Therefore, what the authors see on their EM photomicrographs is claudin 5 that is likely secreted from brain endothelial cells. Moreover, to seal the barrier, it would be necessary that the Cldn5+ protrusions are between brain endothelial cells, and not between endothelial cells and microglia. Most likely microglia is patching pericyte uncovered BBB sites to compensate for the leak. This issue would be critical to resolve by additional carefully performed studies.

Point 4: The authors have added an important piece of data. It is still not clear why Cldn5 and CD68 were chosen? Why the whole dataset from astrocytes was collected but not really discussed or used. Based on the data, the astrocytes gain a very inflammatory phenotype including signatures of leukocyte tethering and rolling. Please discuss.

Point 6: Fig 1d,e is still not very convincing. Authors should use a vascular maker such as Lectin or CD31 along with the different Dextrans. It is confusing that the authors injected the 3 different tracers in the same mouse, one Dextran per animal would be ideal. Also, the color channels need to be split for better clarity. The current method used is not appropriate to study BBB integrity in a rigorous way. Moreover, 70 kDa Dextran shows a nice trend toward leakiness whereas 40 kDa does not, it does not make sense at all. A rigorous regional analysis would be important too.

Point 7: Supplementary Fig 5c, Cldn5 staining actually looks nicer than in the main figures that has a lot of non-specific spots. Please explain and modify the representative images to be of similar quality throughout the whole manuscript.

Point 10 and 11: Videos are still not convincing. The authors should perhaps consider finding other representative videos. Furthermore, over the time course in the representative videos, there might be some photobleaching or damage happening in the imaging field which is causing inconsistency. Please clarify.

Point 21: CD68 expression is surprisingly low still. Please clarify.

Point 25: There are still some problems with terminology related to BBB permeability: Row 520 "the

extravasation of the BBB”.

Additional comments:

If Aqp4 positive end feet are being phagocyted by microglia (as reported herein), then there is very little point in using it for vascular staining. This should be clarified.

Original research showing the anti-inflammatory effects of minocycline was not cited Yrjanheikki et al. PNAS 1999, especially rows 489-490. Please add it and discuss.

Horng et al. JCI 2017 shows how astrocytes can form the tight junctions (Cldn1 and 4). This article should be discussed.

Reviewer #2 (Remarks to the Author):

In my eyes, the group of Hiroaki Wake provides a comprehensive series of experiments describing the dual mode of microglia actions at the BBB during inflammation. During the revision process, the authors added more data, in particular those requested by the reviewers. In addition, they have been more precise in respect to statistics.
The observations are very interesting by providing novel aspects of molecular communication pathways of microglia and the components of the BBB. This work will probably stimulate several others in the field to continue in the suggested directions.
Therefore, this work appears to me ready for publication.

Reviewer #3 (Remarks to the Author):

The extensive revision of the manuscript has addressed most of my concerns.

My main criticism at this stage pertains to the identification of the IBA1+ cell shown in Figure 4e as a microglia, considering that it is located inside the basement membrane. Caution with wording should be used. The cell might be a perivascular macrophage instead of parenchymal microglia.

Reviewer # 1

Point 1. The authors have now looked at the number of pericytes and did not find any changes after LPS challenge. The authors should consider looking at pericyte coverage (see Nikolakopoulou et al., Plos One 2017 for details) which could still be affected. The authors should clarify which brain region is shown in Supp Fig 1. Looking at several region-of-interests such as cortex, hippocampus, and thalamus would be important. Also, the numbers of pericytes should be expressed as the number of PDGFRb+ DAPI+ double positive cells. For coverage, CD13 is probably more reliable marker (see Armulik et al., Nature 2010; Bell et al., Nature 2012).

We believe we have done the experiment initially asked, which showed no change in pericyte numbers in the same areas of the cortex (M1) where we measured BBB permeability, and in both the MRL/lpr and LPS models. Furthermore, we described possible reasons why our results differed from previous studies (namely different LPS doses and different severities of BBB "leak"). We feel to now look at the distribution of pericytes across different brain regions in well beyond the scope of our current report and would also require examination of BBB permeability across these other areas. Imaging pericytes alongside BBB permeability at this level of resolution in hippocampus and thalamus and many other regions is not feasible without significantly more complex surgery and its associated neuronal damage. We have clarified in the legend to Supplementary Figure 1 that we examined pericyte numbers in primary motor cortex. We respectively suggest these additional comments are not justified nor relevant

Point 3: The authors suggest that their EM data show that Cldn5 from microglia contact BM. However, the origin of claudin 5 in their study is unclear, and it is well established that microglia have negligible expression of Cldn5 and that Cldn5 mRNA is only expressed in brain endothelial cells but not in microglia. See for example plots of relative expression on Cldn5 mRNA transcript from two landmark studies frequently cited by many people working in the field – i.e., Betsholtz (Vanlandewijck et al., Nature 2018, 554, 475-480; http://betsholtzlab.org/VascularSingleCells/database.html) and Barres (Zhang et al., J Neurosci 2014, 34.36, 11929-11947; https://web.stanford.edu/group/barres_lab/cgi-bin/igv_cgi_2.py?lname=

Cldn5) databases.

We appreciate that this is a novel (and unexpected) finding that a proportion of microglia express Cldn5 but we feel we have shown good data for this *in vivo* expression of Cldn5 in vessel-associated microglia under these specific conditions. To further evaluate this proposal, we have now performed additional experiments using microglial cultures, and show this data in the revised Supplemental Figure 6. Our new data was performed on both neonatal and adult mouse cultures. Microglia cultures from neonatal mouse brains (P0) expressed neither Cldn5 nor Ccr5, and we could not induce any Cldn5 with application of the CCL5 agonist. In contrast, microglial cultured from adult (9 week) mouse brains did express some basal Cldn5 and this was further enhanced with application of CCR5. We verified the purity of our microglial cultures with FACS (≈95% pure and < 1% contamination with CD31 positive cells). Hence our new data support our proposed hypothesis that microglial cells can express Cldn5 under certain conditions and also form tight junctions to reduce BBB permeability. We are aware of the lovely Stanford RNA seq databases, but cannot specifically identify why Cldn5 RNA was not detected in microglia after LPS injection (Bennett et al., 2016, PNAS; http://www.brainrnaseq.org/), but note that Cldn5 was only associated with a subset (vessel-associated) of microglia, and the dependence on development and/or isolation conditions (new Supplementary Fig 6). We further note that the Stanford database does report Cldn5 in microglia at certain developmental time points (embryonic) and the most recent single cell RNA seq definitively shows Cldn5 expression in three of the six subtypes of microglia they identified from healthy brains from developing and adult mice (Li et al., Neuron, 019 Jan 16;101(2):207-223.e10. doi: 10.1016/j.neuron.2018.12.006; https://myeloidsc.appspot.com/). We have made some additional comments in the discussion.

Therefore, what the authors see on their EM photomicrographs is claudin 5 that is likely secreted from brain endothelial cells. Moreover, to seal the barrier, it would be necessary that the Cldn5+ protrusions are between brain endothelial cells, and not between endothelial cells and microglia. Most likely microglia is patching pericyte uncovered BBB sites to compensate for the leak. This issue would be critical to resolve

by additional carefully performed studies.

We believe a new form of tight junction is made by Cldn5 expressing microglia and Cldn5 expressing ECs, and have EM evidence that microglia make these direct contacts. This may result at sites were pericytes disperse, although we have no evidence for any gross pericyte disruption or loss. We welcome future studies to test this hypothesis further, and recognize in the revised text that we don't as yet have direct functional studies about these MG-EC junctions, although strong supportive data.

Point 4: The authors have added an important piece of data. It is still not clear why Cldn5 and CD68 were chosen? Why the whole dataset from astrocytes was collected but not really discussed or used. Based on the data, the astrocytes gain a very inflammatory phenotype including signatures of leukocyte tethering and rolling. Please discuss.

We chose Cldn5 and CD68 as these are markers of tight junctions and phagocytic phenotypes. We used the dataset to select possible mediators which we then followed up. We appreciate that astrocytes / microglia change their phenotype. In this report, we have focused on the dual role of microglia in BBB permeability changes in inflammation, and the underlying mechanisms, but agree that further detailed studies on how astrocytes may change in inflammation and the physiological consequences would be interesting (but well beyond our scope here).

Point 6: Fig 1d,e is still not very convincing. Authors should use a vascular maker such as Lectin or CD31 along with the different Dextrans. It is confusing that the authors injected the 3 different tracers in the same mouse, one Dextran per animal would be ideal. Also, the color channels need to be split for better clarity. The current method used is not appropriate to study BBB integrity in a rigorous way. Moreover, 70 kDa Dextran shows a nice trend toward leakiness whereas 40 kDa does not, it does not make sense at all. A rigorous regional analysis would be important too.

The leak we report is restricted to small molecules and hence hard to detect. It is different from the large loss of integrity seen in other diseases, as we pointed out on pp4-5. We used the (impermeant) 70kDa dextran as a vessel marker, with the leak

shown by presence of smaller 10kDa fluorescence outside the vessels. We have added a more precise description in the legend to Fig 1d, e. The 70kDa data are not different between control and inflammation mice, our conclusions are based on significant differences, not “trends” within the noise.

Point 7: Supplementary Fig 5c, Cldn5 staining actually looks nicer than in the main figures that has a lot of non-specific spots. Please explain and modify the representative images to be of similar quality throughout the whole manuscript.

This is just due to the merged images. We can put in separate images but just because of the space problem we did this way.

Point 10 and 11: Videos are still not convincing. The authors should perhaps consider finding other representative videos. Furthermore, over the time course in the representative videos, there might be some photobleaching or damage happening in the imaging field which is causing inconsistency. Please clarify.

We can look at the videos again and modify or change. We appreciate any suggestions from the editor.

Regarding the photodamage, we also did imaging in control mice without LPS, as suggested by the reviewer. We did not see any changes due to photobleaching or other off-target damage.

Point 21: CD68 expression is surprisingly low still. Please clarify.

To define CD68 +’ve microglia, The number of CD68 puncta were counted in the microglia. More than two of the CD68 puncta in microglia were defined as CD68 +’ve microglia. That may be the reason that reviewer think CD68 expression is low. However, we are not sure what is low.

Point 25: There are still some problems with terminology related to BBB permeability: Row 520 “the extravasation of the BBB”.

We have changed the phrasing in this sentence.

Additional comments:

If Aqp4 positive end feet are being phagocyted by microglia (as reported herein), then there is very little point in using it for vascular staining. This should be clarified.

We hope the readers consider the use of AQP4 in context, it is widely used as a marker to define the neurovascular unit, as we do here. In later stages we also use as a marker of astrocytic endocytosis.

Original research showing the anti-inflammatory effects of minocycline was not cited Yrjanheikki et al. PNAS 1999, especially rows 489-490. Please add it and discuss.

We have now referred to this reference. Thanks.

Horng et al. JCI 2017 shows how astrocytes can form the tight junctions (Cldn1 and 4). This article should be discussed.

We have now referred to and discussed this reference. Thanks.

Reviewer #2 (Remarks to the Author):

In my eyes, the group of Hiroaki Wake provides a comprehensive series of experiments describing the dual mode of microglia actions at the BBB during inflammation. During the revision process, the authors added more data, in particular those requested by the reviewers. In addition, they have been more precise in respect to statistics.

The observations are very interesting by providing novel aspects of molecular communication pathways of microglia and the components of the BBB. This work will probably stimulate several others in the field to continue in the suggested directions. Therefore, this work appears to me ready for publication.

Reviewer #3 (Remarks to the Author):

The extensive revision of the manuscript has addressed most of my concerns.

My main criticism at this stage pertains to the identification of the IBA1+ cell shown in Figure 4e as a microglia, considering that it is located inside the basement membrane. Caution with wording should be used. The cell might be a perivascular macrophage instead of parenchymal microglia.

I would like to thank for the supportive comment

We first identified a microglia whose cell body was associated with a vessels, and could readily define this as a microglia based on morphology and specific histological criteria associated with microglia in EM (and as described in methods). Since this EM data is 3d SEM, we were then able to trace the processes which extended from this identified cell body and found that the process invaded into the basement membrane as shown and stated in the text. The cell body location was in the parenchyma, and we never saw perivascular macrophages invading into parenchyma. We have added this description into the material and methods.

Reviewers' comments:

Reviewer #1 (Remarks to the Author):

While the authors have addressed some of the comments, several comments are still not adequately addressed and need to be more carefully considered by the authors:

Previous Point 1. The original comment was "The authors should investigate whether or not pericytes are altered in their model". The authors have looked at pericyte number which is good but not sufficient to conclude that they are not altered. Pericytes start losing their processes before complete disappearance, that is why pericyte coverage would be important to investigate otherwise the authors cannot conclude. In other words, reduced pericyte coverage can lead to a breach in BBB integrity without apparent reduced number of cell bodies per se. Without this addition, authors need to remove their statement on page 11: "However, we neither observed any changes in pericyte density of the MRL/lpr mice at baseline nor after LPS injections (Supplementary Fig. 1)". Also, number of pericytes needs to be expressed as PDGFRb+ DAPI+ double positive cells, density does not inform about number of soma nor processes.

Point 3. The authors have partially addressed this concern and added some comments in the discussion as well. Nevertheless, the authors claimed that they do not have evidence of gross pericyte loss which brings us again to point #1 where careful quantification of both number of cells bodies (PDGFRb+ DAPI+ double-positive cells) and pericyte coverage (PDGFRb+ and lectin or CD31, see Nikolakopoulou et al., Plos One 2017; Bell et al. Nature 2010; for details) have to be performed. This additional set of stainings will close up the gap that is currently missing in the manuscript and strengthen the microglia>Cldn5>BBB protection route.

Previous Point 4. The authors were asked to "please discuss" the information collected in this study regarding astrocytes and the potential of their inflammatory phenotype in relation to their study. In what way is the request to add a discussion point "well beyond" the scope of the manuscript?

Previous Point 6. The authors have not adequately addressed this concern. First, the authors injected 3 different size tracers, two of which were tagged with the same fluorophore (40kDa dextran, and 10kDa dextran). How did the authors differentiate between the 40 and 10kDa dextrans? Also, to make the point more clear regarding the request to study BBB integrity in a more rigorous way, the authors should perform staining and quantification of extravasated molecules such as IgG and/or fibrin/fibrinogen to quantify the extent of BBB breakdown in these animals, and recovery after microglial manipulations. Since , this is a key finding in the manuscript to support the authors conclusions these analysis are essential to support claims.

Previous Point 7. The authors wre advised to show individual channels or add images of the individual channels to a supplemental figure for clarity.

Previous Points 10&11: Since the authors also performed imaging in mice without LPS, this information should also be included as supplementary videos to have a comparison to the data presented in supp. Videos 1 and 2.

Finally, supp. movie 2 is a single channel black and white data even though the caption reads that it should be two-channel showing BBB leakage. Please upload the correct movie.

Answer for the Reviewer comments

Thanks very much for your efforts in processing and reviewing our manuscript

Below we describe our response to all the reviewer's comments in further detail.

Reviewers' comments:

Reviewer #1 (Remarks to the Author):

While the authors have addressed some of the comments, several comments are still not adequately addressed and need to be more carefully considered by the authors:

Previous Point 1. The original comment was "The authors should investigate whether or not pericytes are altered in their model". The authors have looked at pericyte number which is good but not sufficient to conclude that they are not altered. Pericytes start losing their processes before complete disappearance, that is why pericyte coverage would be important to investigate otherwise the authors cannot conclude. In other words, reduced pericyte coverage can lead to a breach in BBB integrity without apparent reduced number of cell bodies per se. Without this addition, authors need to remove their statement on page 11: "However, we neither observed any changes in pericyte density of the MRL/lpr mice at baseline nor after LPS injections (Supplementary Fig. 1)". Also, number of pericytes needs to be expressed as PDGFRb+ DAPI+ double positive cells, density does not inform about number of soma nor processes.

We thank the reviewer for raising the distinction around pericyte processes or coverage and pericyte cell numbers. We have now extended the analysis of pericytes from that presented in the previous version, as requested. We measured the number of PDGFRβ and DAPI double positive cells to quantify pericyte number, and used the proportion of PDGFRβ+'ve immunofluorescence co-localized with Lectin +'ve vessels to quantify pericyte coverage. We imaged motor cortex from 5 mice in each condition (WT vs MLR/lpr: Pre, day 1, day 7 of LPS). The new data is shown in Supplementary Figure 3 (panels c-e). We modified the Y axis in Supp Fig 3b to reflect that PDGFRβ+'ve immunofluorescence also reflects pericyte processes and not necessarily cells. We have added the reference to Nikolakopoulou et al (2017) and Bell et al (2010), (and used a more relevant reference for LPS induced loss of pericyte coverage), added the relevant

detail to the methods and modified slightly our description of Supp Fig 3 in the text. All of these changes are highlighted in the text. Our new data support the original conclusions, with no detectable change in pericyte coverage or density. Although it was a feasible proposal that pericyte retraction and cell loss may expose endothelial cells to interact with microglia, we could not detect this in our model. It appears that the subtle changes in BBB permeability we have observed are not associated with pericyte changes, and different from those seen in, for example, to Nikolakopoulou et al (2017) (as also suggested by the lack of fibrinogen leak – see below point#6).

Point 3. The authors have partially addressed this concern and added some comments in the discussion as well. Nevertheless, the authors claimed that they do not have evidence of gross pericyte loss which brings us again to point #1 where careful quantification of both number of cells bodies (PDGFRb+ DAPI+ double-positive cells) and pericyte coverage (PDGFRb+ and lectin or CD31, see Nikolakopoulou et al., Plos One 2017; Bell et al. Nature 2010; for details) have to be performed. This additional set of stainings will close up the gap that is currently missing in the manuscript and strengthen the microglia>Cldn5>BBB protection route.
Please see the response above to point#1, and the new data in Supplementary Figure 3

Previous Point 4. The authors were asked to "please discuss" the information collected in this study regarding astrocytes and the potential of their inflammatory phenotype in relation to their study. In what way is the request to add a discussion point "well beyond" the scope of the manuscript?
We are sorry if our previous response was a bit terse, and agree this is an important area for future work. Along with revisions in the previous version (where we discuss astrocytes upregulating Cldn1, Cldn4 in inflammatory diseases), we have now raised the points that our microarray data indicate possible astrocyte reactivity and that this may also regulate BBB permeability. We have also referred readers to a recent review (McConnell et al., 2019) where the role of astrogliosis in BBB regulation in disease is discussed in more detail. See highlighted text in the revised discussion.

Previous Point 6. The authors have not adequately addressed this concern. First, the authors injected 3 different size tracers, two of which were tagged with the same fluorophore (40kDa dextran, and 10kDa dextran). How did the authors differentiate between the 40 and 10kDa dextrans? Also, to make the point more clear regarding the request to study BBB integrity in a more rigorous way, the authors should perform staining and quantification of extravasated molecules such as IgG and/or fibrin/fibrinogen to quantify the extent of BBB breakdown in these animals, and recovery after microglial manipulations. Since, this is a key finding in the manuscript to support the authors conclusions these analysis are essential to support claims.

We appreciate the reviewer raising this point and appreciate we needed to explain the imaging methods more clearly. We have done this by adding a schematic methods Figure as new Supplementary Figure 1 (referred to in the relevant methods section), and have added more detail in the legend to Figure 1 (highlighted). Briefly, we injected three different fluorescent dextrans of increasing size (10, 40, 70 kDa) with each dextran emitting fluorescence at different wavelengths, corresponding to green and yellow and red. We detected these three colors with different filter sets and in sperate acquisition channels. In the images, we used pseudo coloring to transform both 40 and 70 kDa (yellow and red) to red, so as to contrast the 70kDa green and to enable a merge of both colors to be the distinct yellow. As each dextran was imaged with a different acquisition channel, it was easy to analyze 10 and 40 kDa (and 70kDa) leak out of vessels into the perivascular space.

Regarding using other dyes or molecules to confirm the leak, we don't think other dyes have the same sensitivity and range of defined sizes as the conjugated dextrans – many agents used to test BBB leak in ischemia, trauma or in marked neuroinflammatory conditions are too large (> 100 kDa, including if bound to albumin) for our purposes. Indeed, using a range of dextran-conjugated fluorophores has been considered in the field to be the best approach to quantify BBB permeability (see e.g., *Natarajan, R., Northrop, N., and Yamamoto, B. 2017. Fluorescein isothiocyanate (FITC)-dextran extravasation as a measure of blood-brain barrier permeability. Curr. Protoc. Neurosci. 79:9.58.1-9.58.15. doi: 10.1002/cpns.25*). Nevertheless, we stained the fixed tissue from WT, and MRL/lpr mice, and in mice before and after (7 days) LPS as requested (and also because we wished to evaluate if the BBB permeability we observed was akin to that seen with pericyte disruption). Circulating fibrinogen is about

340 kDa. We did not detect any in the perivascular space under any conditions, and this result has been included as Supplementary Fig 2 and mentioned in the text.

Previous Point 7. The authors were advised to show individual channels or add images of the individual channels to a supplemental figure for clarity.
Thank you for clarifying the point relating to the co-localization of CLDN5 in microglia as shown by immunofluorescence (Fig 4c). We have now added a series of panels in Supplemental Figure 7 illustrating the fluorescence of each individual protein / fluorescence channel, alongside the merged images.

Previous Points 10&11: Since the authors also performed imaging in mice without LPS, this information should also be included as supplementary videos to have a comparison to the data presented in supp. Videos 1 and 2.
We apologize not to have addressed this earlier and thanks for the clarification. We have now added a new Supplemental Video (Video 2) taken over 7 days of saline injections, which serves as a control for Videos 1 and 3.

Finally, supp. movie 2 is a single channel black and white data even though the caption reads that it should be two-channel showing BBB leakage. Please upload the correct movie.
We apologize not to have addressed this earlier. We have now more clearly described the color scheme in the legend (microglia colored as green and 10kDa dextran as red). We have furthermore added an additional Video (Supplemental Video 4) where the 10 kDa dextran in Video 3 was pseudo-colored as gray to more clearly visualize its leakage from blood vessels.

REVIEWERS' COMMENTS:

Reviewer #1 (Remarks to the Author):

The authors have addressed all the concerns.
